# Identifying the causes and consequences of assembly gaps using a multiplatform genome assembly of a bird‐of‐paradise

**DOI:** 10.1111/1755-0998.13252

**Published:** 2020-10-10

**Authors:** Valentina Peona, Mozes P. K. Blom, Luohao Xu, Reto Burri, Shawn Sullivan, Ignas Bunikis, Ivan Liachko, Tri Haryoko, Knud A. Jønsson, Qi Zhou, Martin Irestedt, Alexander Suh

**Affiliations:** ^1^ Department of Ecology and Genetics—Evolutionary Biology Science for Life Laboratories Uppsala University Uppsala Sweden; ^2^ Department of Organismal Biology—Systematic Biology Science for Life Laboratories Uppsala University Uppsala Sweden; ^3^ Department of Bioinformatics and Genetics Swedish Museum of Natural History Stockholm Sweden; ^4^ Museum für Naturkunde Leibniz Institut für Evolutions‐ und Biodiversitätsforschung Berlin Germany; ^5^ Department of Neurosciences and Developmental Biology University of Vienna Vienna Austria; ^6^ Department of Population Ecology Institute of Ecology and Evolution Friedrich‐Schiller‐University Jena Jena Germany; ^7^ Phase Genomics Seattle WA USA; ^8^ Department of Immunology, Genetics and Pathology Science for Life Laboratory Uppsala Genome Center Uppsala University Uppsala Sweden; ^9^ Research Centre for Biology Museum Zoologicum Bogoriense Indonesian Institute of Sciences (LIPI) Cibinong Indonesia; ^10^ Natural History Museum of Denmark University of Copenhagen Copenhagen Denmark; ^11^ MOE Laboratory of Biosystems Homeostasis & Protection Life Sciences Institute Zhejiang University Hangzhou China; ^12^ Center for Reproductive Medicine The 2nd Affiliated Hospital School of Medicine Zhejiang University Hangzhou China; ^13^ School of Biological Sciences—Organisms and the Environment University of East Anglia Norwich UK

**Keywords:** chromosome‐level assembly, GC content, genome assembly, Hi‐C, long reads, satellite repeat, transposable element

## Abstract

Genome assemblies are currently being produced at an impressive rate by consortia and individual laboratories. The low costs and increasing efficiency of sequencing technologies now enable assembling genomes at unprecedented quality and contiguity. However, the difficulty in assembling repeat‐rich and GC‐rich regions (genomic “dark matter”) limits insights into the evolution of genome structure and regulatory networks. Here, we compare the efficiency of currently available sequencing technologies (short/linked/long reads and proximity ligation maps) and combinations thereof in assembling genomic dark matter. By adopting different de novo assembly strategies, we compare individual draft assemblies to a curated multiplatform reference assembly and identify the genomic features that cause gaps within each assembly. We show that a multiplatform assembly implementing long‐read, linked‐read and proximity sequencing technologies performs best at recovering transposable elements, multicopy MHC genes, GC‐rich microchromosomes and the repeat‐rich W chromosome. Telomere‐to‐telomere assemblies are not a reality yet for most organisms, but by leveraging technology choice it is now possible to minimize genome assembly gaps for downstream analysis. We provide a roadmap to tailor sequencing projects for optimized completeness of both the coding and noncoding parts of nonmodel genomes.

## 1 INTRODUCTION

With the advent of next generation sequencing (NGS) technologies, the field of genomics has grown exponentially and during the last 10 years the genomes of almost 10,000 species of prokaryotes and eukaryotes have been sequenced (from NCBI Assembly database, O'Leary et al., 2015). Traditional NGS technologies rely on DNA amplification and generation of millions of short reads (few hundreds of bp long) that subsequently have to be assembled into contiguous sequences (contigs; Goodwin et al., 2016). Although the technique has been revolutionary, the short read length together with difficulties in sequencing regions with extreme base composition poses serious limitations to genome assembly (Chaisson et al., 2015; Peona et al., 2018). Technological biases are therefore impeding the complete reconstruction of genomes and substantial regions are systematically missing from genome assemblies. These missing “unassemblable” regions are often referred to as the genomic “dark matter” (Sedlazeck et al., 2018; Weissensteiner & Suh, 2019). It is key now for the genomics field to overcome these limitations and investigate this dark matter.

Repetitive elements represent an important and prevalent part of the genomic dark matter of many genomes, given that their abundance and repetitive nature makes it difficult to fully and confidently assemble their sequences. This is particularly problematic when the read length is significantly shorter than the repetitive element, in which case it is difficult to anchor the reads to unique genomic regions. To what extent repeats can hamper genome assemblies depends on whether they are interspersed or arranged in tandem. Highly similar interspersed repeats, for example transposable elements (TEs), may introduce ambiguity in the assembly process and cause assembly (contig) fragmentation. On the other hand, tandem repeats are repetitive sequences which are arranged head‐to‐tail or head‐to‐head, such as microsatellites and some multicopy genes (e.g., rRNA genes and genes of the major histocompatibility complex [MHC]). Reads shorter than the tandem repeat array will not resolve the exact number of the repeat unit, resulting in the collapse of the region into fewer copies. Some particular genomic regions enriched for repeats tend to be systematically missing or underrepresented in traditional genome assemblies. These regions include: (a) telomeres at the chromosome ends that are usually composed of microsatellites (Meyne, Ratliff, & Moyzis, 1989); (b) centromeres, essential for chromosome segregation often specified by satellites that can be arranged in higher‐order structures like the alpha satellite in humans (Willard & Waye, 1987) or by transposable elements in flies (Chang et al., 2019); (c) multicopy genes like MHC genes (Shiina et al., 2009); (d) nonrecombining and highly heterochromatic chromosomes like the Y and W sex chromosomes (Chalopin et al., 2015; Hobza et al., 2017; Smeds et al., 2015). As these regions play an essential role in the functioning and evolution of genomes, the need to successfully assemble them is a pressing matter.

The other main limitation of traditional NGS methods is the shortcoming in reading regions with extreme base composition (an enrichment of either A + T or G + C nucleotides), thus representing another source of genomic dark matter. Extreme base composition mainly affects the last step of the standard library preparation for Illumina sequencers that involves PCR (polymerase chain reaction) amplification (Aird et al., 2011; Dohm et al., 2008). GC‐rich regions tend to have higher melting temperatures than the rest of the genome and are thus not as accessible with standard PCR protocols. On the other side of the spectrum, AT‐rich regions are also challenging for amplification with standard PCR conditions and polymerases (Oyola et al., 2012) because they require lower melting and extension temperatures (Su et al., 1996). Several protocols have been developed to help minimize the phenomenon of GC‐skewed coverage (uneven representation of GC‐rich regions), including PCR‐free library preparation (Kozarewa et al., 2009) and enrichment of the GC‐rich genomic fraction prior to sequencing (Tilak et al., 2018). Nonetheless, there is no single method that entirely solves base composition biases of short‐read sequencing and gives a homogeneous representation of the genome (Tilak et al., 2018). As a result, some extremely GC‐rich or AT‐rich regions may not be assembled at all.

It is essential to be aware of technological biases and genome assembly incompleteness during project design. A growing body of evidence suggests that these biases and assembly gaps can affect downstream analyses and mislead biological interpretations (Domanska et al., 2018; Peona et al., 2018; Thomma et al., 2016; Weissensteiner et al., 2017). For example, GC‐skewed coverage is particularly important in birds, where ~15% of genes are so GC‐rich that they are often not represented in Illumina‐based genome assemblies (Botero‐Castro et al., 2017; Hron et al., 2015). Whether these genes are mostly hiding due to technological limitations or truly missing remains of debate (Lovell et al., 2014, Botero‐Castro et al., 2017). However, the “missing gene paradox” in birds is a clear example of how technological biases can shape our view of genome evolution. Furthermore, some GC‐rich sequences can form non‐B DNA structures, that is, alternative DNA conformations to the canonical double helix such as G‐quadruplexes (G4) that can induce strand‐specific sequencing errors and therefore erroneous assembly of the involved regions (Guiblet et al., 2018). G4 structures are four‐stranded DNA/RNA topologies that seem to be involved in numerous cellular processes, such as regulation of gene expression (Du, Zhao, & Li, 2008, 2009; Raiber et al., 2011), genetic and epigenetic stability (Schiavone et al., 2014), and telomere maintenance (Biffi et al., 2012). On the repetitive element side, for example, transposable elements are a major target of epigenetic silencing (Law & Jacobsen, 2010) that may influence the epigenetic regulation of nearby genes (Chuong et al., 2016; Cowley & Oakey, 2013; Tanaka et al., 2019). The epigenetic effect of individual TE insertions may be beneficial or deleterious, but in either case it is important to acknowledge their overall effect on the evolution of gene expression (Lerat et al., 2019). More generally, repetitive elements can play important roles in many molecular and cellular mechanisms, and as a source of genetic variability (Bourque et al., 2018). They have contributed to evolutionary novelty in many organismal groups, by giving rise to important evolutionary features such as the mammalian placenta (Emera & Wagner, 2012), the vertebrate adaptive immune system (Kapitonov & Koonin, 2015; Zhang et al., 2019) and other telomere repair systems (Levis et al., 1993; McGurk et al., 2019). Thus, having genome assemblies that are as complete as possible facilitates research into a multitude of molecular phenomena (Slotkin, 2018).

To achieve more complete genomes, we need new technologies. Recently, long‐read single‐molecule sequencing technologies with virtually no systematic error profile (Eid et al., 2009) have led to more complete and contiguous assemblies (English et al., 2012; Loomis et al., 2013; Pettersson et al., 2019; Smith et al., 2019). To date, two sequencing strategies have been developed that produce very long reads from single molecules: (a) Pacific Biosciences (PacBio) SMRT sequencing, where DNA polymerases incorporate fluorescently labelled nucleotides and the luminous signals are captured in real time by a camera (Eid et al., 2009); and (b) Oxford Nanopore Technologies, which records the electrical changes caused by the passage of the different nucleotides through voltage‐sensitive synthetic pores (Deamer et al., 2016). These new sequencing techniques have already yielded numerous highly contiguous de novo assemblies (Bickhart et al., 2017; Faino et al., 2015; Gordon et al., 2016; Michael et al., 2018; Seo et al., 2016; Weissensteiner et al., 2017; Yoshimura et al., 2019) and helped to improve the completeness of existing ones (Chaisson et al., 2014; Jain et al., 2018), as well as characterize complex genomic regions like the human Y centromere and MHC gene clusters (Jain et al., 2018; Rhoads & Au, 2015; Sedlazeck et al., 2018; Westbrook et al., 2015).

Nevertheless, resolving entire chromosomes remains a difficult endeavour even with single‐molecule sequencing (except for small fungal and bacterial genomes; Ribeiro et al., 2012; Thomma et al., 2016). Even though no single technology is currently able to yield telomere‐to‐telomere assemblies, it is still possible to bridge separate contigs into scaffolds using long‐range physical data and obtain chromosome‐level assemblies (Miga et al., 2020). Such scaffolding technologies are becoming more and more commonly used (Belser et al., 2018; Deschamps et al., 2018; Dudchenko et al., 2017; Li et al., 2019; Rhie et al., 2020; Wallberg et al., 2019). The two most common ones are linked reads (Weisenfeld et al., 2017) and proximity ligation techniques (reviewed in Sedlazeck et al., 2018). Linked‐read libraries are based on a system of labelling short reads belonging to a single input DNA molecule with the same barcode (Weisenfeld et al., 2017). In this way, using high‐molecular‐weight DNA allows us to connect different genomic portions (contigs) that may be distantly located but physically part of the same molecule. High‐throughput proximity ligation techniques such as Hi‐C and CHiCAGO are able to span very distant DNA regions by sequencing the extremities of chromatin loops that can be megabases apart (for more details see Lieberman‐Aiden et al., 2009). While Hi‐C is applied directly on intact nuclei, the CHiCAGO protocol reconstructs chromatin loops in vitro from extracted DNA. All these libraries are then sequenced on the Illumina short‐read platform. As linked reads and proximity ligation techniques are becoming more and more popular, we also implement and test them in the present study.

Although a plethora of new sequencing technologies and assembly methods are currently being successfully implemented, it remains unclear how they complement each other in the assembly process. Here we address these assembly and knowledge gaps using a bird as a model. Bird genomes represent a promising target to investigate because their genomic features make it relatively easy to assemble most parts with the exception of few complex, difficult to assemble regions per chromosome. In fact, the typical avian genome is characterized by a small genome size (mean of ~1 Gb; Gregory, 2019; Kapusta & Suh, 2017) and low overall repeat content (about 10% overall (Kapusta & Suh, 2017). In contrast to the average repeat and GC content of avian genomes, some regions are difficult to assemble, especially the gene‐rich and GC‐rich microchromosomes (Burt, 2002; Griffin & Burt, 2014; Miller & Taylor, 2016) as well as the nonrecombining W chromosome (Bellott et al., 2017; Smeds et al., 2015; Zhou et al., 2014).

To understand which genomic sequences are missing in regular draft genome assemblies with respect to a high‐quality and curated assembly, here we generated several draft de novo genomes and a reference genome for the same sample of the paradise crow (*Lycocorax pyrrhopterus*, “lycPyr”). The paradise crow is a member of the birds‐of‐paradise family (Paradisaeidae), one of the most prominent examples of an extreme phenotypic radiation driven by strong sexual selection, and as such, a valuable system for the study of speciation, hybridization, phenotypic evolution and sexual selection (Irestedt et al., 2009; Ligon et al., 2018; Prost et al., 2019; Shedlock et al., 2004; Xu et al., 2019). We sequenced one female paradise crow individual with all the technologies that worked with a DNA sample of mean 50 kb molecule length. We combined short, linked and long reads together with Hi‐C and CHiCAGO proximity ligation maps into a multiplatform reference assembly. Using the curated assembly, we: (a) demonstrate the feasibility of obtaining a high‐quality assembly of a nonmodel organism with limited sample amount and nonoptimal sample quality (a situation that empiricists commonly face); (b) identify which genomic regions are actually gained from combining technologies compared to draft assemblies of each individual technology; (c) assess the strengths and weaknesses of the implemented technologies regarding the efficiency of assembling difficult repeats and GC‐rich regions; and (d) quantify how technologies can widen or limit the study of specific genomic features (e.g., TEs, satellite repeats, MHC genes, non‐B DNA structures). Finally, we provide a general roadmap for how to investigate previously hidden genomic features.

## 2 MATERIALS AND METHODS

### 2.1 Samples

We used pectoral muscle samples from three vouchered specimens of *Lycocorax pyrrhopterus* subsp. *obiensis* collected on Obi Island (Moluccas, Indonesia) in 2013, from the Museum Zoologicum Bogoriense (MZB) in Bogor, Indonesia, temporarily on loan at the Natural History Museum of Denmark. The individuals were wild‐caught in tropical conditions, fixed at tropical room temperature, and deep frozen only after a couple of weeks. One female (voucher: MZB 34.073) sample (~1 cm^3^) preserved in dimethyl sulphoxide (DMSO; 0.1–0.15 g of tissue stored in 1.5 ml of 8%–9% DMSO solution) was used for PacBio, Illumina and 10X Genomics sequencing and for the Dovetail CHiCAGO library, one female sample (voucher: MZB 34.070) preserved in RNAlater was used for the Hi‐C library with Phase Genomics, and one male sample preserved in DMSO (voucher: MZB 34.075) was used for Illumina sequencing.

### 2.2 Sequencing technologies and de novo assemblies

We sequenced the female sample MZB 34.073 using (a) PacBio RSII C6‐P4 (mean of 11 kb and N50 of 16 kb for read length) for a total coverage of 72×; (b) 10X Genomics with a HiSeqX Illumina machine (24 kb mean molecule length, 280 bp library insert size, 150 bp read length, 750 million reads, net coverage 39.7 × estimated by Supernova); and (c) 10X Genomics with HiSeqX Illumina machine (26.1 kb mean molecule length, 280 bp library insert size, 150 bp read length, 480 million reads, net coverage 37.9 × estimated by Supernova). DNA was extracted with the Kingfisher Duo robot using the KingFisher Cell and Tissue DNA Kit following the manufacturer's recommendation and eluted in 100 µl elution buffer. DNA concentration was then measured with a Qubit fluorometer (ThermoFisher) and finally a few microlitres of DNA was run on a 0.6% agarose gel in 1 × TAE (prepared to separate long DNA fragments) along with a GeneRuler High Range DNA Ladder (ThermoFisher). Agarose gel electrophoresis (Figure S1) indicates that the DNA was slightly degraded and estimated mean molecule lengths (e.g., 24 and 26 kb estimated by Supernova for the 10X Genomics libraries lycPyrSN1 and lycPyrSN2 respectively) much lower than recommended for linked‐read sequencing (i.e., 100 kb, Weisenfeld et al., 2017). DNA of library (c) was extracted with agarose gel plugs as in Weissensteiner et al. (2017). We further used male linked‐read data from Peona et al. (2020). In addition to these libraries, we also used the Illumina libraries and assembly produced by Prost et al. (2019) for the same female sample: Illumina HiSeq 2500 TruSeq paired‐end libraries (180 and 550 bp insert sizes) and Nextera mate pair libraries (5 and 8 kb insert sizes) for a total coverage of 90×. Furthermore, two paired‐end libraries (125 bp read length) of chromatin interactions from CHiCAGO and Hi‐C techniques were produced using a HiSeq 2500 by Dovetail Genomics (insertion size between 1 and 50 kb; Putnam et al., 2016) and Phase Genomics (more details in Text S1), respectively. More details of the libraries produced are given in Table S1. In addition, we used the RNA‐sequencing (RNA‐seq) library from the MZB 34.070 female sample from Peona et al. (2020). Finally, we generated a paired‐end library with insert size of 650 bp on an Illumina HiSeqX machine for the male sample.

For each library/technology (namely Illumina, 10X Genomics and PacBio) we made independent de novo assemblies. Prost et al. (2019) used allpaths‐lg (Butler et al., 2008) for Illumina (“lycPyrIL”) data while we used falcon (Chin et al., 2016) for PacBio data (“lycPyrPB”) and supernova2 (Weisenfeld et al., 2017) for 10X Genomics data (Table 1). The pseudohaploid versions of the assemblies of supernova2 were used throughout the study (“lycPyrSN1,” “lycPyrSN2”). All the basic genome statistics of the assemblies (Table S2) were calculated using the Perl script *assemblathon_stats.pl* from https://github.com/KorfLab/Assemblathon/blob/master/assemblathon_stats.pl including contig/scaffold N50 for which no minimum‐length thresholds were applied.

**Table 1:** Draft and multiplatform assemblies generated for the paradise crow; for each assembly the sequencing technology and software used to produce them are shown together with contig N50, scaffold N50 and the number of gaps

Assembly	Technology	Software	Contig N50 (bp)	No. of contigs	Scaffold N50 (bp)	No. of scaffolds	No. of gaps^a^	Missing assembly^b^ (%)
lycPyrIL	Illumina HiSeq2500 (PE + MP)	allpaths‐lg	620,719	10,766	4,227,710	3,216	14,573	3.82
lycPyrPB	PacBio RSII C6‐P4	falcon	6,644,420	3,422	—	—	—	0.45
lycPyrSN1	10X Genomics Chromium HiSeqX	supernova2	144,856	29,791	4,360,585	13,934	21,550	4.53
lycPyrSN2	10X Genomics Chromium HiSeqX	supernova2	149,640	27,366	4,748,626	14,217	20,131	2.62
lycPyrHiC	PacBio + Phase Genomics Hi‐C	proximo	6,644,420	3,422	70,588,898	2,927	533	0.45
lycPyrILPB	lycPyrIL + gap‐filling with PacBio	pbjelly	1,982,606	6,895	4,229,628	3,216	10,422	3.03
lycPyr2	PacBio + Dovetail CHiCAGO	hirise	6,294,665	3,463	6,644,037	3,227	282	0.45
lycPyr3	lycPyr2 + 10X Genomics	arcs + links	6,294,665	3,463	8,009,555	3,121	345	0.27
lycPyr4	lycPyr3 + Phase Genomics Hi‐C	proximo	6,294,665	3,463	69,071,023	1,713	1,791	0.27
lycPyr5	lycPyr4 + manual curation with alignments + gap filling	pbjelly	7,540,011	3,269	74,173,823	1,700	1,631	0.001
lycPyr6	lycPyr5 + manual curation with Hi‐C	juicer	7,540,011	3,271	74,173,823	1,700	1,635	—

Abbreviations: MP, mate pair reads; PE, paired end reads.

^a^ The number of gaps is estimated as the count of stretches of *N* nucleotides within a scaffold.

^b^ The percentage of incompleteness is relative to the final version of the multiplatform assembly: (1 − (assembly size/final assembly size)) × 100. The *N* nucleotides are excluded from the calculation.

### 2.3 Identification of sex‐linked contigs and PAR

Given the conservation of the Z chromosomes across songbirds (Xu et al., 2019), we used the Z‐chromosome sequence of great tit (*Parus major*) as a query to search for homologous Z‐linked contigs in paradise crow. The aligner nucmer (Kurtz et al., 2004) was used to perform the one‐to‐one alignment of the great tit genome and lycPyrPB. Contigs with more than 60% of their sequence aligned to the great tit Z chromosome were identified as putatively Z‐linked. We further calculated the sequencing coverage using the female Illumina paired‐end (180 bp insert size) library to confirm the half‐coverage pattern of candidate Z‐linked contigs relative to autosomal contigs. We used bwa‐mem (Li & Durbin, 2010) to map the reads and the samtools (Li et al., 2009) depth function to estimate contig coverage. To identify candidate W‐linked contigs, we calculated the resequencing coverage of the male individual, because W‐linked contigs are female‐specific and are not expected to be mapped by male reads while the coverage of female reads should be half of that of autosomes. We used the known pseudoautosomal region (PAR) sequences of collared flycatcher (*Ficedula albicollis*; Smeds et al., 2014) to identify the homologous PAR contigs in paradise crow while considering male/female coverage ratios. As expected, the PAR contigs were found to show similar resequencing coverage in both the male and the female as on the autosomes (Figures S2 and S3).

### 2.4 Multiplatform approach

We created three types of multiplatform assemblies, one that combines only Illumina and PacBio data (“lycPyrILPB,” see Table 1), a second one combining PacBio and Hi‐C data, and a third more comprehensive one that combines three types of sequencing data and two types of proximity ligation data (“lycPyr6”).

For the first type of assembly (lycPyrILPB), we used the Illumina assembly lycPyrIL (Prost et al., 2019) as genomic backbone and gap‐filled it with PacBio long reads using the software pbjelly (pbsuite version 15.8.24) maintaining all the default options except ‐min 10 to consider only gaps of at least 10 bp in length. The second multiplatform assembly, “lycPyrHiC,” was built by scaffolding the PacBio primary assembly (lycPyrPB) with Hi‐C data.

**FIGURE 1:**
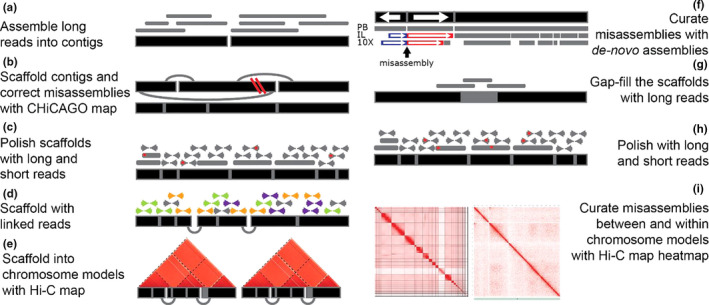
Overview of the multiplatform assembly process. (a) Long reads were assembled into contigs. (b) The primary assembly was corrected and scaffolded using long‐range information provided by the CHiCAGO proximity ligation map. (c) The assembly was then polished from base‐calling errors with both short and long reads and (d) further scaffolded with linked reads. (e) The scaffolds were ordered and oriented into chromosome models according to the Hi‐C proximity ligation map. (f) The chromosome models were aligned to the draft de novo assemblies based only on one single technology and then manually inspected to correct misassemblies following the majority rule (more details in Figure 2 and Methods). PB: PacBio long‐read assembly; IL: Illumina short‐read assembly; 10X: 10X Genomics linked‐read assemblies. (g) Long reads were used to gap‐fill the assembly and (h) to polish the final version together with short reads. (i) Hi‐C heatmaps were used to identify and correct misassemblies between and within chromosome models. This multiplatform approach is similar to those of large‐scale sequencing projects such as the Bat1K Initiative (Teeling et al., 2018) and the Vertebrate Genomes Project (Rhie et al., 2020) [Colour figure can be viewed at wileyonlinelibrary.com]

For the most comprehensive assembly (lycPyr6), we combined PacBio, Illumina, 10X Genomics, CHiCAGO and Hi‐C data (Figure 1). We used the PacBio primary assembly (lycPyrPB) as the genomic backbone of our assembly (Figure 1a). Then we proceeded to correct and scaffold the contigs with the CHiCAGO map (Figure 1b) and polished sequencing errors (Figure 1c; lycPyr2) with long reads and short reads. Next, we scaffolded the assembly further with linked reads (Figure 1d; lycPyr3) and the HiC map to obtain a chromosome‐level assembly (Figure 1e; lycPyr4). The assembly was manually curated by comparing it to the draft assemblies and outgroup species (Figure 1f), gap‐filled (Figure 1g), and polished from sequencing errors with long and short reads (Figure 1h; lycPyr5). Finally, we manually inspected the Hi‐C interaction map to solve ordering and orientation issues within the chromosome models (Figure 1i; lycPyr6). All details of the assembly protocol can be found in the Text S1.

The completeness of the assemblies was assessed with busco version 3 for avian genomes (Table S3) using the online platform gVolante (Nishimura et al., 2017), a gene presence/absence analysis using a newly produced gene annotation for paradise crow (Table S4), the percentage of properly mapped paired‐end reads (using the lycPyrSN1 library and samtools stats) and with the LTR Assembly Index (LAI; Ou et al., 2018; Table S5). The differences in scaffolding, fragmentation and identity between assembly versions were assessed and visualized by using the online tool d‐genies (Cabanettes & Klopp, 2018) (Figure S4).

The mitochondrial genome was identified as a single PacBio contig by aligning the mitochondrial DNA (mtDNA) of *Corvus corax* (GenBank accession no. KX245138.1) to lycPyrPB. It was annotated using dogma (Wyman et al., 2004) and trnascan‐se 1.3.1 (Lowe & Eddy, 1997; Table S6).

### 2.5 Chromosome nomenclature

The chicken genome is the best avian genome assembled so far with reliable chromosome information (Warren et al., 2017), and therefore we named and oriented our chromosome models according to homology with galGal5 (RefSeq accession no. GCF_000002315.6), except for chromosomes 1 + 1A and 4 + 4A which were named according to the zebra finch (Warren et al., 2010). In the case that our chromosome models were not completely collinear with chicken, we oriented them following the orientation of the majority of the model with respect to chicken. Finally, if the chromosome models did not share any homology with chicken, their orientation was not changed.

### 2.6 Gene annotation and gene presence/absence analysis

To measure how many genes were absent in the draft assemblies with respect to the final version and to have an alternative overview of completeness based on genes, we first annotated the genes on the sex chromosomes and then aligned the protein sequences to all the paradise crow assemblies.

We annotated the genes on the sex chromosomes of lycPyr6 through maker (Cantarel et al., 2008) using both an evidence‐based and an ab initio approach. For the evidence‐based annotation, we used pectoral muscle RNA‐seq data from Peona et al. (2020) of the same individual used here for Hi‐C data. The transcriptome was assembled with trinity (Haas et al., 2013) and stringtie (Pertea et al., 2015) together with the protein sequences of chicken downloaded from Uniprot. The ab initio annotation was done using augustus (Stanke et al., 2006) and snap (Korf, 2004) trained on the avian gene models from busco (Simão et al., 2015). The two annotations were combined, classified using interproscan (Quevillon et al., 2005) and annie (Ooi et al., 2009) and can be found in Table S4.

Next, we used the protein‐coding gene sequences identified on lycPyr6 as query for the splice‐aware aligner exonerate (Slater & Birney, 2005). Genes were considered: (a) “complete” if their protein sequences aligned for at least 95% of their lengths with a similarity higher than 90%; (b) “partial” if the protein sequences aligned but for less than 95% of their lengths; and (c) “absent” if no alignment could be found (Table S7).

### 2.7 Repeat library

We produced a de novo repeat library for paradise crow by running the repeatmasker 4.0.7 and repeatmodeler 1.0.8 software on the PacBio de novo assembly. We hard‐masked lycPyrPB with the Aves repeat library from Repbase (version 20170127; Bao et al., 2015) together with the consensus sequences from Prost et al. (2019), then ran repeatmodeler. Each new consensus sequence generated by repeatmodeler was aligned back to the genome assembly; the 20 best blastn 2.7.1 + results were collected, extended by 2 kb on both sides and aligned to one another with mafft 7.4.07. The alignments were manually curated applying the majority rule and the superfamily of repeats assessed following the Wicker et al. (2007) classification.

All the new consensus sequences were masked in censor (http://www.girinst.org/censor/index.php) and named according to homology to known repeats in the Repbase database. Sequences with high similarity to known repeats for their entire lengths (80%; Wicker et al., 2007) were given the name of the known repeat + suffix “_lycPyr”; repeats with partial homology have been named with the suffix “‐L_lycPyr” where “L” stands for “like” (Suh et al., 2018). Repeats with no homology with known ones have been considered as new families and named with the prefix “lycPyr” followed by the name of their superfamilies.

The final repeat library also contains the manually curated version of the consensus sequences previously generated on two other birds‐of‐paradise, namely *Astrapia rothschildi* “astRot,” and *Ptiloris paradiseus* “ptiPar” (Prost et al., 2019), curated repeats from *Corvus cornix* (Weissensteiner et al., 2019), *Uraeginthus cyanocephalus* (Boman et al., 2019) and *Ficedula albicollis*, and all the avian repeats available on Repbase (mostly from chicken and zebra finch).

### 2.8 G4 motif identification

The de novo assemblies and the final version were scanned for G‐quadruplex (G4) motifs with the software quadron (Sahakyan et al., 2017). Only nonoverlapping hits with a score greater than 19 were used for subsequent analysis as suggested by Sahakyan et al. (2017). The density of such motifs per chromosome model was calculated using bedtools coverage (bedtools 2.27.1; Quinlan, 2014).

### 2.9 MHC class IIB analysis

To infer how highly duplicated genes are assembled with different input data and assembly strategies, we investigated the distribution of major histocompatibility class IIB (MHCIIB) sequence hits in seven assemblies: lycPyrIL, lycPyrPB, lycPyrSN1, lycPyrSN2, lycPyrILPB, lycPyr2 and lycPyr6. The results for the intermediate assemblies lycPyr3–5 are not shown here because the MHC content did not change in these relative to lycPyr2. We performed blast (Altschul et al., 1990) searches both with sequences of the highly variable exon 2 that encodes the peptide binding region, and with the much more conserved exon 3 (Hughes & Yeager, 1998), as the disparate levels of polymorphism within these regions may provide insights into different aspects of challenges with genome assembly. We conducted tblastn (blast 2.7.1+) searches using alignments available from Goebel et al. (2017) that include sequences from across the entire avian phylogeny. We chose this strategy to ensure the identification of MHCIIB sequences, because single‐species blast searches might miss highly divergent sequences as they are often present in the MHC, where within‐species diversity of MHC genes often equals between‐species divergence. From the available alignments, we exclusively retained sequences spanning the entire 270 bp of exon 2 and sequences covering 220 bp of exon 3. This left query alignments including 233 sequences from 22 bird orders/families for exon 2, and 314 sequences from 26 bird orders/families for exon 3. Overlapping blast hit intervals were merged. To ensure that these intervals contained sequences corresponding to MHCIIB, we first blast searched them back against the GenBank database using blastn queries, and retained only intervals producing hits with MHCIIB. We then aligned the remaining sequences using the mafft alignment server with the ‐‐add option and default settings, and manually screened the alignments to identify non‐MHCIIB sequences. Finally, we determined the alignment lengths of blast hit intervals after removing insertions relative to the query alignment. We report only hits longer than 240 bp for exon 2 and longer than 195 bp for exon 3, corresponding to ~90% of the respective query alignment lengths. Finally, we mapped the PacBio reads to every scaffold and contig with identified MHC loci to assess the quality of the assembled regions by inspecting their coverage pattern.

### 2.10 Gap analysis

For each assembly produced, we investigated: (a) the causes of assembly gaps by analysing the nature of the sequences adjacent to the gaps, and (b) the content of the gaps of draft assemblies by lifting over the gap coordinates onto the final assembly and analysing the sequences within the lifted‐over gaps.

We estimated the number of gaps caused by repeats by intersecting the gap and repeat coordinates using bedtools window (Quinlan, 2014) with a window size of 100 bp (Figure 4a). Only gaps longer than 10 bp were taken into consideration. This filter is particularly important for lycPyrIL because there are many small gaps of 1–5 N nucleotides that are probably caused by sequencing or base‐calling errors.

We estimated what is missing in the draft assemblies with respect to the final multiplatform assembly lycPyr6 by aligning the flanking regions to the gaps onto the final version. We then assessed the presence of annotated repeats on lycPyr6 between the aligned flanking regions to the draft assembly gaps. To make these pairwise alignments, we extracted 500 bp of flanking regions from the intrascaffold gaps of lycPyrIL, lycPyrSN1, lycPyrSN2, lycPyrPB and lycPyrILPB, and blastn searched the sequences to lycPyr6 with blast 2.7.1+. The alignments were filtered to retain only unambiguously orthologous positions on lycPyr6, namely there was only one alignment (98% identity, 90% coverage, 1e‐20 e‐value cutoff) of both flanks on the same lycPyr6 scaffold. The coordinates of the draft genome gaps projected onto lycPyr6 were then intersected with the repeatmasker annotation using bedtools intersect. Draft genome gaps containing only one type of repeat on lycPyr6 were classified according to the type of repeat. If draft genome gaps corresponded to a region containing more than one type of repeat, the gaps were classified as “complex”. Finally, in cases where draft genome gaps could not be mapped unambiguously (i.e., no homology, only one flank aligned, or the two flanking regions mapped to different scaffolds) or mapped to gaps on lycPyr6, they were classified as “not scorable gaps” (Figure 4a).

We also compared how many repeats were assembled in the draft assemblies compared to lycPyr6 (Figure 4b) by calculating the proportion of repeat base pairs present in the draft assemblies relative to the total base pairs in lycPyr6. This was done for each major repeat group using the repeatmasker table (.tbl) files; more details are given in Table S8.

## 3 RESULTS

We utilized the power of data generated from multiple sequencing approaches for the same sample of paradise crow to generate a high‐quality assembly and to assess limitations of regular draft genomes based on any single technology. Briefly, we combined short, linked and long reads with proximity ligation data to obtain a high‐quality assembly despite the limitations of a nonmodel organism such as limited sample amount and nonoptimal quality. For each sequencing technology, we produced an independent de novo assembly. These assemblies were compared using majority‐rule decisions by manually curating the final assembly. Finally, the multiplatform assembly was compared to each de novo version to assess the amount of repeats and other complex regions previously missing from the individual assemblies. We then evaluated the completeness of each assembly using a variety of different metrics, including established scores such as busco, contig/scaffold N50, LAI and new metrics such as overall repeat content, number of MHCIIB exons, GC and G4 content, as well as the number and nature of gaps.

### 3.1 Draft de‐novo assemblies

To compare the efficiency of short, linked and long reads, we produced independent draft assemblies for each of the different sequence libraries. One draft genome assembly of paradise crow based on short reads (Illumina) is already available from Prost et al. (2019) (“lycPyrIL”; Table 1). For the present study, we produced two linked‐read libraries (10X Genomics Chromium) from which we assembled two draft genomes (“lycPyrSN1” and "lycPyrSN2”; where “SN” stands for Supernova) and a PacBio library from the same sample to generate the primary assembly “lycPyrPB” (Table 1 and see Methods section). In total, four independent de novo assemblies were generated.

We first evaluated the completeness of these assemblies by assessing their fragmentation, contig and scaffold N50, and by counting the number of core genes present with busco (Nishimura et al., 2017; Waterhouse et al., 2017). In terms of fragmentation, the PacBio primary assembly (“lycPyrPB”) consisted of about 3,000 contigs, while lycPyrIL had ~10,000 contigs (~3,000 scaffolds), and the 10X Genomics assemblies had ~29,000 contigs (~14,000 scaffolds; Table 1). Similarly, lycPyrIL had a contig N50 of 620 kb, lycPyrSN1 and lycPyrSN2 had a contig N50 of ~145–150 kb, while lycPyrPB had a contig N50 of 6 Mb (Table 1, Table S2). Notably, there is a 10‐fold higher contig N50 in lycPyrPB relative to the lycPyrIL assembly, indicating significant improvement in assembly contiguity in the PacBio versus Illumina assembly. Next, we used the busco tool (Nishimura et al., 2017) to identify correctly assembled core genes (percentage of only single‐copy and complete genes): lycPyrIL 93.8%, lycPyrSN1 92.5%, lycPyrSN2 91.5%, lycPyrPB 84.8%, the last before any assembly polishing (Table S3). Similarly, we generated an in‐depth gene annotation of the sex chromosomes of lycPyr6 to test how many genes were missing in the other assembly versions. Using paradise crow RNA‐seq data from Peona et al. (2020), the maker pipeline identified 719 protein‐coding genes on the Z and W sex chromosome models. We found that up to five genes were completely missing from the draft assemblies, while between 22 (lycPyrILPB) and 122 genes (lycPyrSN2) were fragmented in short‐read or linked‐read assemblies, and only three to 21 genes were fragmented in long‐read assemblies (Table S7). In addition, we checked for the percentage of properly mapped paired‐end reads on all the assembly versions (Table S9). Although the percentage of properly mapped reads over the total number of mapped paired‐end reads is nearly identical across assemblies (99%), the absolute number of properly mapped reads is higher in lycPyrSN2, lycPyr5, lycPyr6 and lycPyrSN1 with respect to lycPyrIL. Interestingly, gap filling of lycPyrILPB increased the number of properly mapped reads to the level of lycPyrSN1. Finally, we estimated genome completeness and quality of the intergenic and repetitive sequences with the LAI (Ou et al., 2018). This index is calculated as the proportion of full‐length LTR retrotransposons over the total length of full‐length LTR retrotransposons plus their fragments. LAI could only be calculated for lycPyrPB because the other de novo assemblies did not have enough full‐length LTR retrotransposons for the algorithm to work. lycPyrPB has an LAI score of 11.89, which is typical of a reference‐quality assembly (Ou et al., 2018), and higher than chicken (galGal5, RefSeq accession no. GCF_000002315.6; Bellott et al., 2017) with an LAI score of 7.54. We cannot exclude that the higher score in paradise crow is caused by biological differences in LTR load between the species. More details of the LAI score distribution across chromosomes and genomes are given in Table S5, and Figures S5 and S6.

### 3.2 The multiplatform reference assembly

To generate a high‐quality genome assembly, we combined five technologies (short, linked and long reads in addition to a CHiCAGO and Hi‐C proximity ligation maps) into one multiplatform assembly. This process was divided into nine steps (Figure 1), described in further detail in the Methods section and the Text S1.

First, we assembled the PacBio long reads into the primary assembly (lycPyrPB; 3,442 contigs) and scaffolded and corrected for misassemblies with the Dovetail CHiCAGO map (“lycPyr2”; Figure 1a,b). The scaffolding software hirise introduced 98 breaks and made 293 joins of scaffolds (gaps of 100 bp were introduced at this stage), as well as closed 11 gaps between contigs, resulting in an assembly of 3,227 scaffolds (Table 1; Table S2). Subsequently, we polished the assembly with long reads (two rounds of arrow; Chin et al., 2016) and short reads (two rounds of pilon; Walker et al., 2014; Figure 1c). According to Schmidt et al. (2017), polishing with pilon already reaches a plateau at the second or third iteration, so we reasoned that two rounds of polishing with long and short reads would be sufficient at this stage. Later in the assembly process, further rounds of polishing were applied. The assembly polishing with arrow resulted in one correction per 415 bp in the first round and one correction per 6,735 bp in the second round. The polishing with pilon (focusing only on indels) corrected ~300,000 indels in the first round and ~180,000 in the second.

We then continued to scaffold lycPyr2 with two types of long‐range information in order to get a chromosome‐level assembly. First, we used 10X Genomics linked reads (SN1 library; 24 kb mean molecule length; Figure 1d) that encode medium‐range spatial information that placed 235 contigs into 131 new scaffolds (gaps of 10 bp were introduced at this stage). Of these new scaffolds we kept only 88 and discarded potential chimeric scaffolds, which were identified by being composed of putatively sex‐linked contigs and autosomal contigs (based on male/female short‐read coverage; see Methods). We confirmed the chimeric nature of such discard scaffolds by constructing an additional assembly based on scaffolding lycPyrPB with the Hi‐C map (lycPyrHiC; Table 1). Phase Genomics Hi‐C (i.e., 3D chromatin conformation data) can bridge sequences that are megabases apart (Burton et al., 2013), and theoretically reconstruct entire chromosomes (Hi‐C super‐scaffolds). In this way, lycPyrHiC represented an independent verification of the synteny or chimeric nature of the contigs. Accordingly, we checked whether the contigs resided on different Hi‐C super‐scaffolds. Once we split the chimeric scaffolds, we obtained lycPyr3 which contained a total of 3,121 scaffolds. Second, we scaffolded lycPyr3 with Phase Genomics Hi‐C and obtained 38 super‐scaffolds (lycPyr4; Figure 4e) that harboured 1,446 contigs/scaffolds and accounted for 97% of the assembly length, while 1,675 contigs/scaffolds remained unplaced (3% of the assembled genome). As most of these super‐scaffolds (32 out of 38) correspond to entire chromosomes of other avian species (e.g., zebra finch and chicken), we consider them as “chromosome models.” Examining the post‐scaffolding Hi‐C heatmap, we found that chromosomes 1 and 2 were split into two Hi‐C super‐scaffolds, respectively. Therefore, following the high level of Hi‐C interaction between these super‐scaffold pairs in the heatmap (Figure S7), we manually combined the respective super‐scaffold pair into one chromosome model (see Methods); the assembly thus resulted in 36 chromosome models.

**FIGURE 2:**
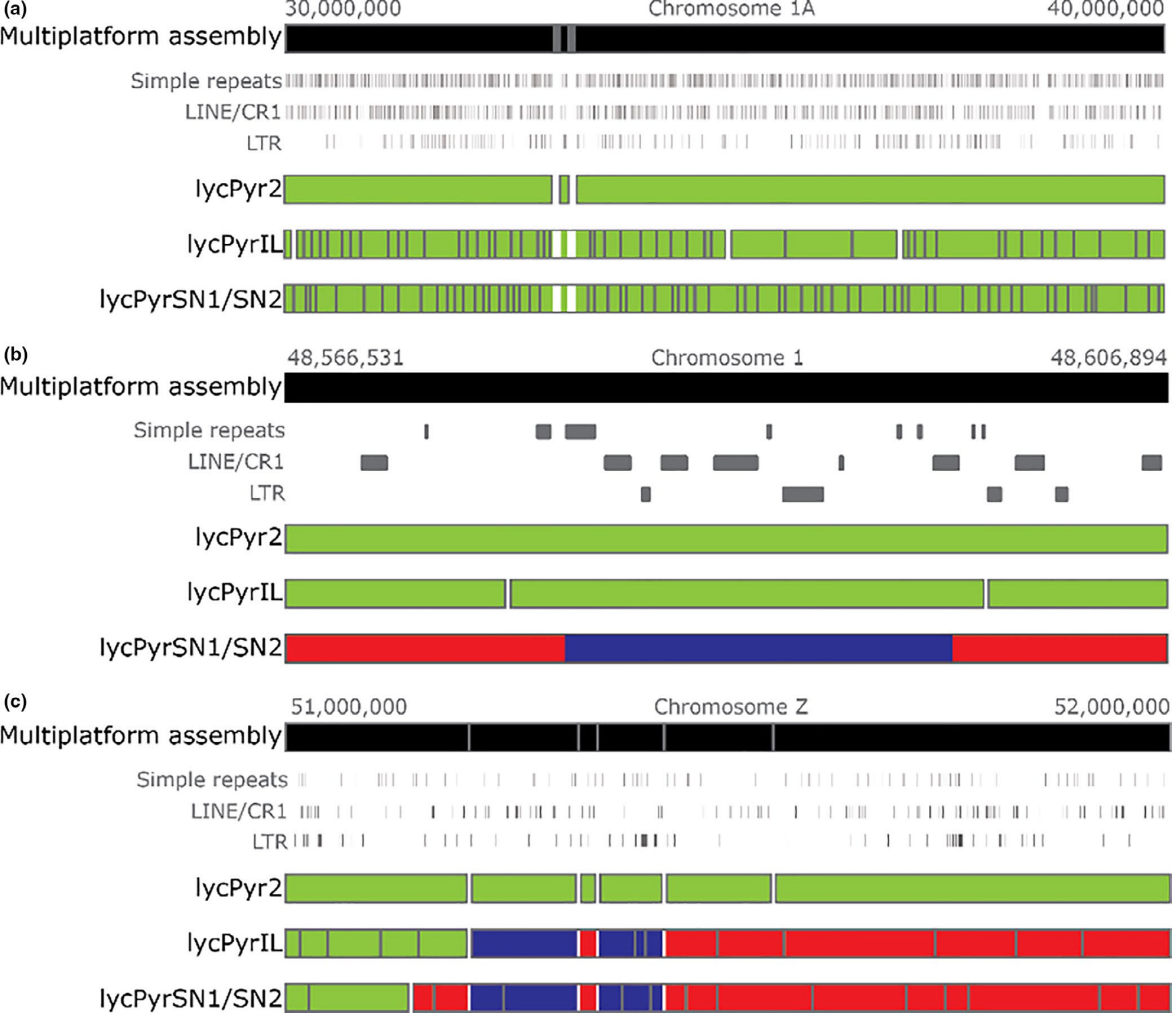
Examples for the manual curation step of the assembly process (step f in Figure 1). The multiplatform assembly is aligned to the draft de novo assemblies. The grey lines within the assemblies represent gaps between different contigs of the same scaffold while the white lines represent gaps between scaffolds. Green indicates contigs/scaffolds that align to the reference in the same orientation for their entire length, while red and blue highlight contigs/scaffolds that partially align in the forward (red) and reverse (blue) direction relative to the multiplatform assembly. (a) Here 10 Mb of chromosome 1A are shown that are in accordance with all de novo assemblies. Note the much more fragmented scaffolds in the assemblies based on short reads. (b) Example of a scaffold orientation misassembly in the 10X Genomics assembly. The other two assemblies span the inverted region and both agree with the multiplatform assembly. (c) Example of a discordance suggesting contigs to be re‐oriented and re‐ordered in the final assembly. In lycPyrIL, lycPyrSN1 and lycPyrSN2, some scaffolds span the misoriented (blue) region and bridge it to contigs that show concordant orientation with the multiplatform assembly [Colour figure can be viewed at wileyonlinelibrary.com]

We proceeded to further manually curate the chromosome models by looking for misassemblies (Figure 1f) and used long reads for gap‐filling (Figure 1g). We corrected fine‐scale orientation issues of contigs within scaffolds through whole‐genome alignments (see Figure 2 and Methods) and corrected more orientation, order issues and erroneous chromosomal translocations through the inspection of Hi‐C heatmaps (see Figure 1i and Methods). We first corrected 43 misassemblies by aligning the draft genomes and three outgroups to lycPyr4 (see Figure 2 and Methods; Tables S10 and S11, Figure S8). Next, we extended contig ends and filled scaffold gaps with long reads using pbjelly (lycPyr5). pbjelly filled 106 gaps, extended 56 gaps on both ends and extended only one end of 292 gaps (Table S12). Finally, we further checked for misassemblies with the help of the Hi‐C data. We generated a Hi‐C heatmap of lycPyr5 with juicer (Durand et al., 2016) and detected misassemblies though the visual inspection of such a map with juicebox (Dudchenko et al., 2018) following the recommendations given by Lajoie et al. (2015) and Dudchenko et al. (2018). The Hi‐C heatmap showed mostly orientation and ordering problems within lycPyr5 (Figure S9) that can be identified from ribbon‐like and checkered patterns in the interaction map (Dudchenko et al., 2018). Finally, the map highlighted the misplacement of two contigs between chromosome models (Figure S9). In total, 76 misassemblies were corrected in this step to generate the final assembly (lycPyr6) with a super‐scaffold N50 of ~75 Mb (Table 1).

In parallel with the assembly of lycPyr6, we also generated a simpler multiplatform assembly by gap‐filling the Illumina primary assembly (lycPyrIL) with PacBio reads (lycPyrILPB). pbjelly was used to gap‐fill the Illumina assembly and successfully closed 4,151 gaps, reducing the total number of gaps from 14,573 to 10,422. It also double‐extended 418 gaps and single‐extended 2,597 gaps (Table S12). The numbers of scaffolds and scaffold N50 did not significantly change from lycPyrIL (Table 1).

### 3.3 Chromosome models: Macrochromosomes, microchromosomes and sex chromosomes

We obtained 36 chromosome models comprising 16 macrochromosome models, 18 microchromosome models and two sex chromosome models. All the macrochromosome models showed homology to chicken galGal6a chromosomes and were named after their homologous counterparts. The same applies for 12 of 18 microchromosomes, while the remaining six showed no homology with chicken chromosomes and therefore were tentatively named as unknown chromosomes “chrUN1–6.” The chromosomes homologous to chicken are mostly syntenic with respect to chicken with a few exceptions. In fact, chicken chromosomes 1 and 4 are split in two in Passeriformes and correspond, respectively, to chromosome 1 and 1A, and chromosome 4 and 4A (Kapusta & Suh, 2017; Warren et al., 2010).

**FIGURE 3:**
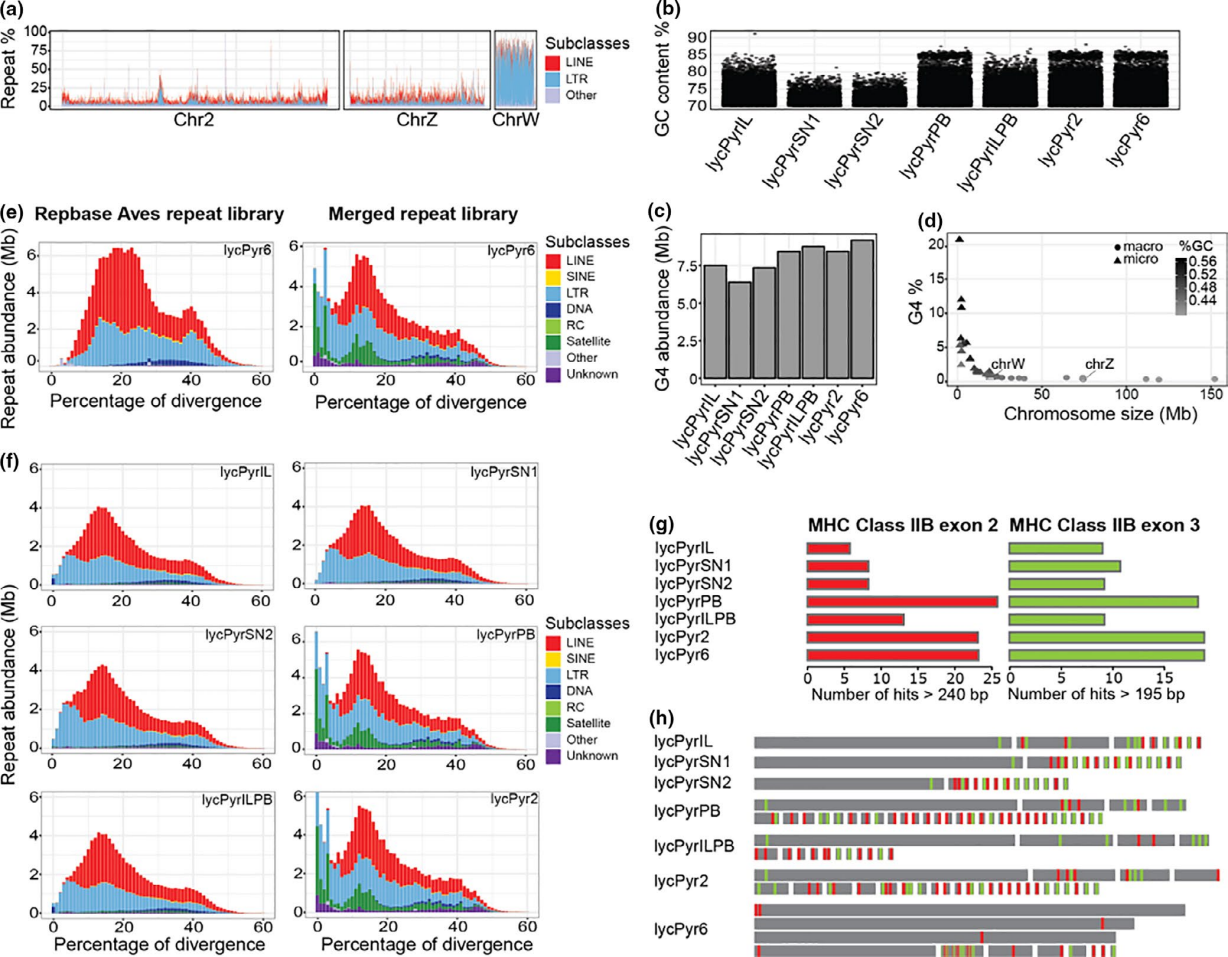
(a) Comparison of the repeat content across autosomes (chromosome 2 as a representative) and the Z and W sex chromosomes. Percentage of repeat‐derived base pairs shown per window of 50 kb. Here LINE and LTR are shown as major components of the repetitive element repertoire and all the other types of repeats are merged into the “Other” category. (b) Distribution of GC‐rich windows across different assemblies. Shown are 1‐kb windows with a GC content >70%. (c) Abundance of G4 motifs in megabases across different assemblies. (d) Percentage of G4 motif base pairs across chromosome models of the final assembly. Chromosomes are arranged by size; macrochromosomes are coloured in light grey while microchromosomes (<20 Mb) are shown in dark grey corresponding to higher GC content. The percentage of G4 in micro‐ and macrochromosomes is statistically different (*t* test, *p*: 0.01). (e) Repeat landscape of lycPyr6 masked with the Repbase Aves repeat library (on the left) and masked with the custom library produced in this study which also included the Repbase Aves library (on the right). (f) Repeat landscapes of the different assemblies masked with the custom repeat library. (g) Abundance of paralogues of MHC class IIB exon 2 and exon 3 in the different assemblies. (h) Schematic visualization of the paralogues of MHC class IIB exon 2 (red) and 3 (green) present in the different assemblies. Each grey rectangle represents a different contig or scaffold separated by white lines [Colour figure can be viewed at wileyonlinelibrary.com]

The Z and W sex chromosome models had an assembled size of 73.5 and 21.4 Mb, respectively, and were comparable to chicken (82 and 7 Mb, galGal6a, RefSeq accession no. GCF_000002315.6; Bellott et al., 2017). Z and W chromosome models were also largely consistent with the sex‐linked contigs we identified using male/female coverage comparisons (Table S13, Figures S2 and S3 and Methods); only 3.11 Mb of the W and 3.99 Mb of the Z were contigs not identified as sex‐linked based on coverage ratios. The gene annotation made on these two chromosome models confirms their classification given the presence of the respective gametologues (based on chicken genes, Table S4). Finally, the PAR seemed to be fragmented into two parts. We identified two contigs that are homologous to the PAR of flycatcher; one of them was placed by Hi‐C onto the Z chromosome while the other was placed onto the W chromosome model (Table S13). While the Z chromosome showed a repetitive content similar to the autosomes (~10%), the W was extremely repeat‐rich (~70%, Figure 3a; Table S14). Dotplots of the alignments of the paradise crow sex chromosomes with the chicken sex chromosomes (Figures S10 and S11) showed that the two Z chromosomes had a high level of synteny and collinearity while the repetitiveness of the two W chromosomes made it difficult to identify shared single‐copy regions other than very short ones. The sex chromosomes were also easily identified in the post‐clustering Hi‐C heatmap (Figure S9), as their hemizygosity can be expected to result in roughly half of the amount of Hi‐C interactions (calculated as the frequency of shared paired‐end reads between contigs/scaffolds) within each chromosome model and with the other chromosome models.

Finally, we estimated the assembly quality of each chromosome model separately using the LAI (Ou et al., 2018). This index is calculated as the ratio of identified base pairs of full‐length LTR retrotransposons over the base pairs occupied by fragmented LTRs. The LTR retrotransposons are known to be difficult to assemble because of their length (several kb) and identical terminal repeats in the same orientation. Their structure makes it likely to have full‐length elements assembled only in high‐quality assemblies. The LAI quantifies the proportion of intact LTR elements and can be a proxy of the quality of the intergenic and repetitive sequence space (Ou et al., 2018). The LAI calculation yielded high scores (min. 0 on chromosome 10, mean 13.14, max. 21.41 on chromosome W) that have been suggested to indicate reference and gold‐quality assemblies (Ou et al., 2018, Figure S5 and Table S5).

### 3.4 GC content and G4 motif prediction

GC‐rich regions are commonly underrepresented in traditional NGS assemblies because of the aforementioned GC‐skewed coverage phenomenon (see Introduction). Comparing the different de novo assemblies, we noted that lycPyrPB indeed showed more GC‐rich regions (54,532 windows of 1 kb size with GC > 58.8%) with respect to lycPyrIL, SN1 and SN2 (45,966, 45,720 and 52,080 such windows, Figure 3b; Table S15, Figure S12).

Because GC‐rich regions may form G‐quadruplex motifs and structures (G4), we expected the depletion of GC‐rich short reads to limit the representation of G4 motifs in short‐read assemblies. Conversely, we expected G4 motifs to be more abundant in long‐read assemblies, as these have been suggested to be virtually free from sequence‐based biases (Eid et al., 2009). To test this, we predicted the presence of G4 motifs using quadron (Sahakyan et al., 2017) in all the different assemblies. All the de novo Illumina‐based assemblies had fewer predicted G4 motifs than the PacBio assemblies (Figure 3c; Table S16). lycPyrSN2 and lycPyrIL had 7.3 and 7.5 Mb (169,214 and 166,602 motifs) occupied by G4 sequences and about 1.6 Mb or 24,000 motifs fewer than lycPyr6 (9.1 Mb, 193,248 motifs). lycPyrSN1 was the assembly with the fewest G4 motifs predicted (6.5 Mb, 149,275 motifs). The PacBio primary assembly lycPyrPB had 8.42 Mb of predicted G4, while lycPyr2, after correction with Dovetail CHiCAGO, had 8.43 Mb (Figure 3c; Table S16). In the final assembly lycPyr6, G4 motifs were more present on microchromosomes than on macrochromosomes (Figure 3d).

### 3.5 Repeat library

To obtain an in‐depth annotation of interspersed and tandem repeats, de novo characterization of repetitive elements and manual curation thereof are essential (Platt et al., 2016). We manually curated a total of 183 consensus repeat sequences generated from lycPyrIL and lycPyrPB to give an optimal repeat characterization. In Prost et al. (2019), a total of 112 raw consensus sequences were produced using repeatmodeler on three Illumina‐based birds‐of‐paradise (*Astrapia rothschildii*, *L. pyrrhopterus* and *Ptiloris paradiseus*; including lycPyrIL) but only the 37 most abundant from lycPyrIL were manually curated. We then curated the remaining 75 and added 71 more de novo consensus sequences based on curated raw consensus sequences from repeatmodeler run on lycPyrPB. Our new bird‐of‐paradise‐specific repeat library is now composed of the following numbers of consensus sequences: 56 ERVK, 56 ERVL, 37 ERV1, five CR1, four LTR, nine satellites, one SINE/MIR and 13 unknown repeats. All the consensus sequences curated for the three species of birds‐of‐paradise (*L. pyrrhopterus*, *A. rothschildii*, *P. paradiseus*) are given in Table S17. Next, we merged birds‐of‐paradise consensus sequences together with the Repbase Aves library and libraries from flycatcher (Suh et al., 2018), blue‐capped cordon bleu (Boman et al., 2019) and hooded crow (Weissensteiner et al., 2019).

**FIGURE 4:**
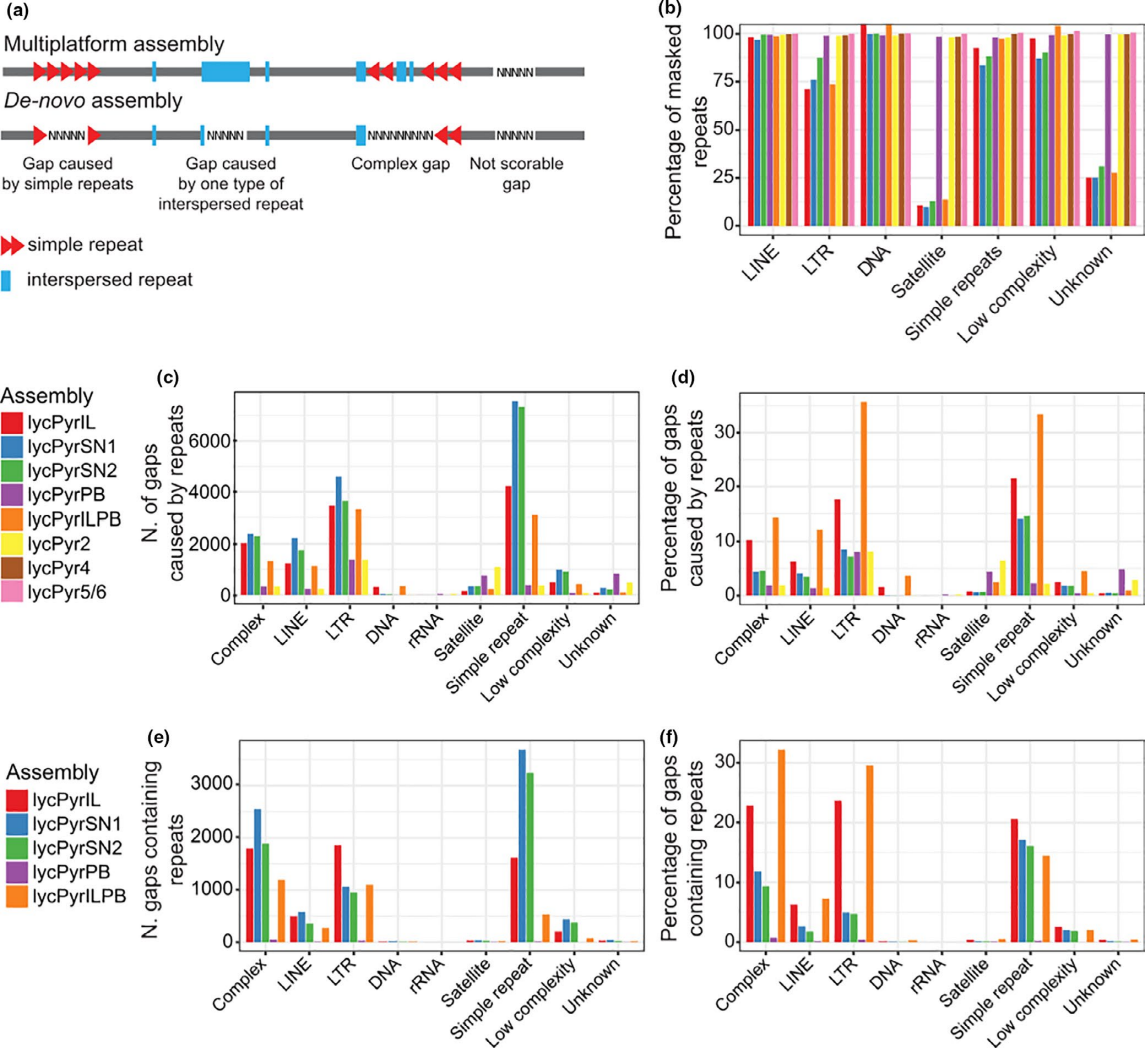
The causes of gaps in different assemblies compared to the final multiplatform assembly (lycPyr6). (a) Schematic representation of how gaps were categorized based on the flanking regions and content relative to lycPyr6. (b) Proportion of repeats present in each assembly version with respect to lycPyr6. (c) Number of gaps caused by the major repeat groups. (d) Proportion of gaps caused by the major repeat groups. (e) Number of gaps that correspond to repeats in lycPyr6. (f) Proportion of gaps that correspond to repeats in lycPyr6 [Colour figure can be viewed at wileyonlinelibrary.com]

Custom and de novo repeat libraries substantially improve the identification and masking of repeats in genome assemblies (Platt et al., 2016). To quantify this effect for our assemblies, we compared a general avian repeat library with our curated one. The custom library resulted in masking a higher fraction of the genome in every assembly (Figure 3e,f). When comparing the masked fraction with the custom library to the fraction masked with the Repbase library, the assemblies lycPyrIL, lycPyrILPB and lycPyrSN1 have 20% more masked repeats (an increase from 78 to 94 Mb), while lycPyrSN2 has 21.68% (from 83 to 101 Mb), lycPyrPB 38% (from 87 to 120 Mb), and lycPyr6 38% (from 88 to 122 Mb; see Figure 3e; Table S8). Notably, with the new library we were able to identify 9.4 Mb of satellites in the PacBio‐based assemblies, while the standard Repbase avian library identified only 1 Mb (Figure 3e,f; Table S8). Relative to multiplatform assembly lycPyr6, most of the satellites and unknown repeats remain unassembled in the short‐read and linked‐read assemblies (Figures 3f and 4b).

### 3.6 MHC class IIB analysis

In birds, the multicopy gene family of the MHC is arranged as a megabase‐long tandem repeat array (Miller & Taylor, 2016). Because such arrays are expected to be even more difficult to correctly assemble than the aforementioned interspersed repeats (O’Connor et al., 2019), we consider it a prime candidate region for measuring the quality of an assembly.

We used the presence of entire copies of the second (most variable) and third (more conserved) exons of the MHC class IIB as proxies of assembly quality (Hughes & Yeager, 1998). Overall, we found that short‐read assemblies had fewer MHC gene copies than long‐read assemblies (Figure 3g,h), while linked‐read assemblies performed better than Illumina alone. Regarding exon 2 (Figure 3g), lycPyrPB had 26 copies while Illumina and 10X Genomics assemblies had only six to eight. However, it is worth noting that the correction of lycPyrPB with the Dovetail CHiCAGO map led to the absence of three copies in downstream multiplatform assemblies. The results were similar for exon 3 (Figure 3g): lycPyrPB contained 18 copies while the other technologies had only none to 11 copies. In this case the molecule input length of the 10X Genomics library had an effect on the assembly of these genes, where shorter molecule length corresponded to more copies compared to the assembly based on longer molecules (11 vs. 9 exon 2 copies; Figure 3g,h). On the other hand, while Dovetail CHiCAGO decreased the number of exon 2 copies, it increased the number of assembled exon 3 copies to 19. The contigs where the MHC loci were identified often show coverage levels similar to the surrounding regions, but some had an extremely high or low coverage (Figure S13).

### 3.7 Gap analysis

The process of scaffolding links together contigs without adding any information about the missing DNA between them, but it is possible to use long reads to fill those gaps. For this we utilized pbjelly (English et al., 2012) to extend and bridge contigs in the assembly by locally assembling PacBio reads to the contig extremities. Once the software finds reads aligned to the contig extremities, the extremities can be: (a) extended on one or both sides to reduce the gap length, (b) extended and bridged to fill the entire gap, and (c) extended over the length of the gap without ultimately being bridged (overfilled). pbjelly extended the extremities of 348 gaps, closed 116 gaps and overfilled 236 gaps (Table S12). This gap‐filling step added a total of 2.96 Mb to the assembly. All the sequences that were extended or gap‐filled were more GC‐rich (40%–89%, mean 58%) than the average GC content of 40%, and 2,865 G4 motifs were added for a total of 171 kb. Only 800 kb of the 2.96 Mb added correspond to annotated repetitive elements; specifically, ~400 kb of LTR retrotransposons were added, 120 kb of LINE retrotransposons, 142 kb of satellites, and 90 kb of simple and low‐complexity repeats (Table S12).

Furthermore, we investigated the causes of assembly fragmentation in different assemblies by analysing the immediate adjacency of repetitive elements to the gaps (lower part of Figure 4a). Due to alignment or assembly issues (described in the Methods section), part of the gaps could not be analysed (“Not scorable gaps” in Figure 4a). We found that simple repeats were the major fragmentation cause in Illumina and 10X Genomics assemblies, followed by LTR and LINE retrotransposons (Figure 4c,d). In contrast, PacBio gaps (lycPyrPB and lycPyr2) seemed to be mainly caused by LTR retrotransposons and secondarily by satellites (Figure 4c,d).

Finally, we quantitatively and qualitatively assessed which repeats in the final multiplatform assembly lycPyr6 were collapsed as gaps in the draft assemblies (Figure 4e,f). Many gaps in the Illumina and 10X Genomics draft assemblies corresponded to complex regions consisting of multiple types of repetitive elements (Figure 4e,f). Among draft assembly gaps containing only a single type of repeat in lycPyr6, most were caused by simple repeats, LTR retrotransposons and LINE retrotransposons in short‐read and linked‐read assemblies (Figure 4e,f). Notably, this pattern was also visible in the Illumina assembly lycPyrILPB despite the long reads used for gap filling (Figure 4e,f).

## 4 DISCUSSION

Assembling complete eukaryotic genomes is a complex and demanding endeavour often limited by technological biases and assembly algorithms (Alkan et al., 2010; Sedlazeck et al., 2018). In the last decade, NGS technologies have defined the standard of genome assemblies. Although they provide an unprecedented view on the structure and evolution of many coding regions (Zhang et al., 2014), short reads hardly inform on the entire complexity of a genome (Thomma et al., 2016). Indeed, the systematic absence from genome assemblies and the difficulty in characterizing the nature of many such genomic regions (e.g., centromeres, telomeres, other repeats and highly heterochromatic regions) gave these “unassemblable” sequences the evocative name of genomic “dark matter” (Johnson et al., 2005; Weissensteiner & Suh, 2019).

In this study, we have demonstrated that a combined effort involving multiple state‐of‐the‐art methods for long‐read sequencing and scaffolding yielded a high‐quality reference for a nonmodel organism. We showed that a multiplatform approach was highly successful in resolving elevated quantities of genomic dark matter with respect to single‐technology assemblies (regular draft assemblies) and thus resulted in a much more complete assembly. To assess genome completeness, we focused mostly on the quantification and characterization of previously inaccessible regions within genomic dark matter, such as large transposable elements, GC‐rich regions and the high‐copy MHC locus.

We generated a de novo multiplatform assembly of a female bird‐of‐paradise genome by combining the cutting‐edge technologies that are now being implemented in many assembly projects (Bickhart et al., 2017; Faino et al., 2015; Gordon et al., 2016; Michael et al., 2018; Rhie et al. 2020; Seo et al., 2016; Teeling et al., 2018; Weissensteiner et al., 2017; Yoshimura et al., 2019), namely Illumina short reads, 10X Genomics linked reads, PacBio long reads and two proximity ligation maps with Dovetail CHiCAGO and Phase Genomics Hi‐C. The choice of using a bird‐of‐paradise is manifold. First, avian genomes are small among amniotes and have an overall repeat content of 10%, which make most genomic regions relatively “easy” to assemble. This has made it possible to focus on regions that are challenging to assemble in eukaryotic genomes of any size and complexity, like the repeat‐rich W sex chromosome, and the GC‐rich microchromosomes. Second, birds‐of‐paradise represent a highly promising system for the study of speciation, hybridization and sexual selection (Irestedt et al., 2009; Prost et al., 2019; Xu et al., 2019). A gold standard genome for this family will consequently expose new possibilities for more in‐depth studies of the genomic evolution behind the spectacular radiation of birds‐of‐paradise.

By employing a multiplatform approach, we (a) could assemble a chromosome‐level genome which includes the W chromosome and several previously inaccessible microchromosomes (i.e., comparable to the chicken genome, so far the best avian genome available); (b) report that a substantial proportion (up to 90%) of repeat categories such as satellites and LTR retrotransposons are missing from most types of de novo assemblies (Figures 3e,f and 4b); and (c) identify simple repeats and LTR retrotransposons as the major causes of assembly fragmentation (Figure 4c,d).

### 4.1 A chromosome‐level assembly for a nonmodel organism

Our final assembly comprises 36 chromosome models. This assembled chromosome number is similar to the known karyotype of another bird‐of‐paradise species, *Ptiloris intercedens* (36–38 chromosome pairs; L. Christidis, personal communication). Among these models, there are 16 macrochromosomes, 12 microchromosomes, and the Z and W sex chromosomes showing homology to chicken chromosomes (galGal6a). The remaining six models do not share homology with known chicken chromosomes (galGal6a) and might be putatively uncharacterized microchromosomes. Microchromosomes are known to be very GC‐rich (Burt, 2002) and indeed this trend is also present in our data (Figure 3d). Base composition can create biases during the sequencing process especially when a PCR step is required for library preparation (Aird et al., 2011; Dohm et al., 2008), thus limiting the representation of GC‐rich and AT‐rich reads in the data. Although long‐read sequencing technologies such as PacBio have reduced amplification‐based biases to a minimum (Schadt et al., 2010 but see Guiblet et al., 2018), we could not assemble contiguous sequences for all microchromosomes. Among the unknown or unassembled chromosomes, chromosome 16 is one of the most complex avian chromosomes and also contains the MHC (Miller & Taylor, 2016). The fragmentation of these microchromosomes is probably linked to their very high GC content and density of G4 motifs relative to the other chromosomes (Figure 3d). Given that the DNA polymerase tends to introduce sequencing errors in the presence of G4 structures (Guiblet et al., 2018), it is tempting to think that the depletion of the smallest microchromosomes from assemblies is not only due to GC content per se but also due to the potential presence of non‐B structures (e.g., G4) with which elevated GC content appears to correlate. Nonetheless, even with the extensive use of cytogenetics, the last chicken assembly (galGal5; Warren et al., 2017) completely lacks the five smallest microchromosomes. It thus seems plausible that these chromosomes need particular efforts to be recovered. For the paradise crow, we lack estimates of its C‐value but related species of Corvidae have C‐values of 1.24 (~1.22 Gb, Christidis, 1990). As our assembly size is similar to that of related species, similar amounts of DNA are likely to be missing (Peona et al., 2018).

One of the most surprising outcomes of this multiplatform approach is the successful assembly of the highly repetitive W chromosome, which turned out to be larger (assembly size 21 Mb) and more repetitive than the chicken equivalent (assembly size 7 Mb; Bellott et al., 2017). In both species, it is likely that the assembled sequences cover the euchromatic portions of the W. Birds have a ZW sex chromosome system in which the female is the heterogametic sex and the female‐specific W is analogous to the mammalian male‐specific Y chromosome. Comparable to the mammalian Y (Charlesworth et al., 2000), the W chromosome is highly repetitive and difficult to assemble (Weissensteiner & Suh, 2019). Previous studies focusing on the repetitive content of the avian W in chicken (Bellott et al., 2017) and collared flycatcher (Smeds et al., 2015) showed in both cases a repeat density of about 50%. In our assembly of the paradise crow, we found the W chromosome to be even more repetitive, with a repeat density of ~70% and strong enrichment for LTR retrotransposons (Figure 3a; Table S14). Having well‐assembled chromosomes is key to improving any genomic analysis, but studies on sex chromosome evolution in birds have so far been heavily biased towards Z (Xu et al., 2019; Yazdi & Ellegren, 2018; Zhou et al., 2014). With genome assemblies such as here, it will be possible to improve reconstructions on how the two sex chromosomes diverged. We can already see that the W chromosome evolves rapidly (Figure S10) via accumulation of transposable elements and only a few regions appear syntenic and collinear between paradise crow and chicken W.

### 4.2 How complete are genome assemblies?

Previous studies (e.g., Etherington et al., 2019; Paajanen et al., 2019) have assessed the efficiency of available sequencing technologies in genome assembly and genome completeness mainly through summary statistics such as scaffold N50 and BUSCO values. Scaffold N50 indicates the minimum scaffold size among the largest scaffolds making up half of the assembly, while BUSCO values measure the number of complete/incomplete/missing core genes in the assembly. However, genome completeness goes beyond scaffold N50 and gene presence (Domanska et al., 2018; Sedlazeck et al., 2018; Thomma et al., 2016). Genes usually occupy a small fraction of genomes and new sequencing technologies commonly yield high N50 values. Therefore, these statistics have a very limited scope given what the new sequencing technologies can achieve.

Although often used as a proxy for assembly quality, scaffold N50 is hardly meaningful in this regard because it does not inform about the completeness and correctness of the assembled sequences. If we order the scaffolds by decreasing size, scaffold N50 value can only reflect the fragmentation level of the first half of the assembly regardless of whether the second half is made up of shorter sequences. Finally, contig N50 should be used as a measure of contiguity, rather than scaffold N50, as contig length measures sequences not interrupted by gaps.

Most of the currently available avian genomes score more than 95% of BUSCO gene completeness (Peñalba et al., 2019) with various degrees of fragmentation, suggesting that it has become straightforward to generate short‐read assemblies with high BUSCO values. On the other hand, BUSCO seems to be limited by the sequencing errors introduced by PacBio in the identification of gene models (Watson & Warr, 2019). Even with multiple rounds of error correction, BUSCO fails to recognize genes that are actually present, at least partially, in the assembly (Watson & Warr, 2019). This tendency is also evident from our results; for example, after gap‐filling lycPyrIL with long reads, 10 BUSCO genes were no longer detectable in the resulting assembly lycPyrILPB (Table S3). A similar dynamic was observed during the assembly process of the superb fairy‐wren *Malarus cyaneus* (Peñalba et al., 2019) where BUSCO values dropped with long‐read gap‐filling but were restored after sequence polishing. Therefore, the polishing step of long‐read assemblies is necessary to get a good gene annotation (and BUSCO score). Moreover, BUSCO seems to be trained and based on a set of core genes identified from Sanger and Illumina assemblies. Although these set of gene models are accurate, they are just a subset of less difficult‐to‐assemble core genes. Especially in birds, hundreds of core genes are missing (“hidden genes”) from short‐read assemblies because of their GC content (Botero‐Castro et al., 2017), and would therefore be missing from BUSCO gene sets. This means that, theoretically with assembly polishing, short‐read and long‐read assemblies can reach the same high BUSCO scores, but the score would inevitably ignore the presence and quality of difficult‐to‐assemble core genes. In organisms such as birds where even short‐read assemblies routinely yield high BUSCO scores of >95% (Peñalba et al., 2019), additional gene‐based approaches are needed to quantify gene presence and completeness (e.g., using “hidden genes”). As a complementary approach to BUSCO, we aligned the gene models of chromosomes Z and W to all the assembly versions and identified complete/partial/absent genes. This approach showed that despite high BUSCO values, the short‐read and linked‐read based assemblies contain many fragmented gene models. This indicates that when genes from challenging regions of the genome are taken into consideration, the difference in gene content between short‐read, linked‐read, and long‐read assemblies is highlighted.

Long reads have the potential to assemble very repetitive regions (e.g., MHC) and elusive chromosomes (e.g., W and microchromosomes). For this reason, quality assessment should rely upon measuring the efficiency in assembling difficult regions and not on those regions that we have already obtained with previous technologies. We therefore decided to measure genome completeness and quality by characterizing and quantifying these difficult regions.

Long reads were instrumental, not only to find and mask more repeats, but also to assemble and discover previously overlooked repetitive sequences. In fact, by adding PacBio sequence data we were able to significantly increase the number of predicted repeat subfamilies compared to the repeat library previously built on Illumina assemblies from three birds‐of‐paradise species (from 112 to 183 consensus sequences; Prost et al., 2019). These 71 new consensus sequences were only predicted by repeatmodeler using the PacBio assembly, probably because the respective repeats were too fragmented or assembled in too few copies in Illumina assemblies. A clear example is given by the satellite repeats that are severely depleted from both the lycPyrIL assembly (Figures 3e,f and 4b) and from the previous repeat library. With our new repeat library, we could increase the base pairs masked by repeatmasker by up to 38% within the same assembly (lycPyr6). This indicates that while longer read lengths are important for assembling repeats, only with a comprehensive and curated repeat library can we quantify the actual efficiency of different technologies.

Repetitive elements are not only made up of transposable elements and satellite repeats, but also of multicopy genes. One of the most repetitive gene families is the MHC involved in the adaptive immune response. In birds, MHC genes are prevalently located on one of the most difficult chromosomes to assemble, namely chromosome 16 (Miller & Taylor, 2016). We recovered several scaffolds from this chromosome for which the only, albeit fragmented, assembly exists from chicken (Warren et al., 2017). We counted how many MHCIIB copies we could retrieve in the different assemblies, using blast hits to exon 2 and 3 sequences as proxy. We found the maximum number of copies in lycPyrPB (Figure 3g,h) followed by lycPyr6, suggesting that the misassembly correction with the CHiCAGO map affected the MHC genes, with the number of hits for exon 2 decreasing and for exon 3 increasing. Short‐read assemblies harbour fewer MHCIIB exon copies but we note that linked reads could assemble two more copies compared to standard Illumina short reads. Although throughout this study we use lycPyr6 as the assembly closest to the “true genome,” this does not mean it is perfectly assembled. Indeed, the genomic regions containing the MHC genes have variable coverage values indicating possible assembly issues. For such complex regions, the use of ultralong reads or bacterial artificial chromosome (BAC) sequencing seems necessary to resolve the number of gene copies and their structure.

As a further use of repetitive elements as quality measures, we tested the LAI (Ou et al., 2018) that assesses the quality of an assembly from the completeness of the LTR retrotransposons present. It was not possible to obtain values for the Illumina and 10X Genomics assemblies because the tool requires a certain baseline quantity of the full‐length LTR assembled to run as initial requirements. Nonetheless, both lycPyrPB and lycPyr6 show LAI scores (respectively 11.89 and 13.59; Table S5, Figure S5) typical for high‐quality reference genomes (as indicated in Ou et al., 2018) and higher than those of chicken (Figure S6). The increase in LAI value from lycPyrPB and lycPyr6 indicates that the assembly curation process, mostly gap filling and polishing, improved the quality of the multiplatform assembly with regard to the representation of large LTR retrotransposons.

In addition to repetitive elements, base composition is the other main factor that limits completing genome assemblies. We thus assessed the GC content per window for each assembly (Figure 3b; Figure S12) and, as expected, found more GC‐rich windows in lycPyrPB compared to the other de novo assemblies (Figure S12). High GC content is often associated with non‐B DNA structures such as G4 that have been shown to introduce sequencing errors during polymerization (Guiblet et al., 2018). We found that Illumina and 10X Genomics assemblies have about 1.6–2.6 Mb less of G4 motifs compared to lycPyrPB (Figure 3c), and in this case linked reads did not help to gain a more complete overview of this genomic feature with respect to regular Illumina libraries. On the other hand, the overall curation from lycPyrPB to lycPyr6 improved G4 prediction. G4 motifs influence various molecular mechanisms such as alternative splicing and recombination (Maizels & Gray, 2013), and therefore more complete assemblies make these regions accessible for comparative genomic analysis.

### 4.3 Strengths and limitations of sequencing technologies

Nowadays, we have a plethora of sequencing technologies to choose from, each with its own advantages and limitations. Additionally, the large number of assembly tools available and hundreds of parameters to tweak makes it inevitable to produce numerous different assembly versions. For example, we generated 15 different assemblies only for the parameter optimization of the linked‐read scaffolding (Figure 1d) and there are studies generating even 400 assemblies in total (Montoliu‐Nerin et al., 2020). In such a situation, it might seem difficult to decide how to choose the “best” assembly among dozens. Here we present what we learned from the different technologies and how they help to resolve the genomic regions that are most difficult to assemble.

We used two types of de novo assemblies based on Illumina sequencing. The first, lycPyrIL, is an Illumina assembly made from multiple insert size libraries of paired‐end and mate‐pair reads (Prost et al., 2019); the second is on 10X Genomics linked reads (lycPyrSN1 and SN2). It is notable that lycPyrIL is much more contiguous than lycPyrSN1 and lycPyrSN2 (contig N50 of 620 kb vs. 145–150 kb; Table 1) and has much fewer gaps. Although lycPyrIL is a less fragmented assembly, lycPyrSN2 has a better resolution for repeats because 7 Mb more repeats are masked and a larger number of MHCIIB exons are present (Figure 3g,h) as well as G4 motifs (Figure 3g). Nonetheless, the contiguity reached in lycPyrPB for the same sample at the contig level (contig N50 of 6 Mb) is 10‐fold higher than in lycPyrIL and even outscores the lycPyrIL scaffold N50 of 4 Mb.

10X Genomics linked reads provide long‐range information through the barcode system that is useful for local phasing, detection of structural variations (Marks et al., 2019; Zheng et al., 2016), scaffolding (Yeo et al., 2017) and construction of recombination maps (Dréau et al., 2019; Sun et al., 2019). We used the barcode information to scaffold the PacBio assembly (lycPyr3, Table 1) without obtaining many new scaffolds but this could be due to the already high contiguity of the input lycPyrPB assembly. Finally, we note that the molecule input lengths for the 10X Genomics libraries have different effects on the assembly and BUSCO scores. That is, lycPyrSN1 (24 kb mean molecule length library) outscores lycPyrSN2 (26.1 kb mean molecule length library) in the number of complete BUSCO genes (Table S3). Even though 10X Genomics linked reads consist of Illumina short reads, both lycPyrSN1 and lycPyrSN2 have more missing genes compared to lycPyrIL (Table S3). The quality of linked reads and associated draft assemblies (as well as long reads) relies heavily upon the quality of the extracted DNA and high‐molecular‐weight DNA is necessary to wholly take advantage of these sequencing technologies.

Long reads together with proximity ligation maps are game changers in genomics. Their combination yielded a very high‐quality assembly for a nonmodel bird with suboptimal sample quality (see mean molecule lengths for 10X Genomics assemblies above). The PacBio assembly is by far the most contiguous and a suitable genomic backbone to obtain chromosome models including the W chromosome and several microchromosomes. The main weakness linked to PacBio is the introduction of sequencing errors (mostly short indels) that must be corrected with accurate short reads. As mentioned before, the sequencing errors hinder gene model identification (BUSCO) and protein prediction (Watson & Warr, 2019). However, the recent availability of PacBio HiFi reads should increase accuracy up to 99.8%, matching the accuracy of short reads (Wenger et al., 2019). Moreover, the PacBio assembly is probably not free of misassemblies (e.g., chimeric contigs). Thus, a second type of independent data is necessary to detect such errors; for example, ~100 potential misassemblies were identified by the CHiCAGO proximity map. The CHiCAGO map was very useful to correct the assembly and make a first scaffolding, but neither alone nor with 10X Genomics scaffolding did it yield a chromosome‐level assembly. The only type of data implemented here that allowed the generation of chromosome models was the Hi‐C map. The latter does not rely on extracted DNA quality or library insert size, but instead on in situ proximity within the nuclei of the fixed sample. Although high‐density linkage maps and cytogenetics are the best source for assessing chromosome models and their quality (Deakin et al., 2019), Hi‐C can be an effective alternative until such data are available especially for nonmodel organisms.

A direct way to identify the limits of sequencing data is to investigate where assemblers fail to resolve sequences (i.e., where contig fragmentation occurs). Therefore, we characterized what causes contig fragmentation in each assembly by analysing sequences directly adjacent to gaps and inferring the gap content of draft assemblies by aligning their flanks to the final multiplatform version, lycPyr6 (Figure 4a). In general, we found that long and/or homogeneous repeats such as LTR retrotransposons, satellites and simple repeats are the main causes of fragmentation in every assembly, although the prominence of the specific repeat type changed with the technology. Short‐read and linked‐read contigs mostly break at simple repeats. Even though the percentage of simple repeats assembled in lycPyrIL, lycPyrSN1 and lycPyrSN2 ranges between 80% and 90% relative to lycPyr6 (Figure 4b), simple repeats also caused most of the assembly gaps, indicating that short reads with different insert size or linked‐read barcoding are not sufficient to unambiguously resolve those regions (Figure 4c,d). At the same time, the gaps of these three draft assemblies, when compared to the final multiplatform assembly, mainly contain LTR retrotransposons, simple repeats and complex repeats (defined as arrays of different types of repeats; Figure 4e,f).

LTR retrotransposons are the second most abundant retrotransposons in the paradise crow assembly and are often several kilobases long. These features make LTR retrotransposons the major cause of fragmentation in the PacBio assembly and the second in the other draft assemblies. This partially unexpected trend is probably because LTR retrotransposons are underrepresented in lycPyrIL, lycPyr SN1 and lycPyrSN2 (as indicated by their lack of part of the recent LTR activity; Figure 3e,f). The same pattern can be observed for the multicopy rRNA genes: the only assemblies showing gaps caused by rRNA genes are the PacBio‐based ones and this is probably because PacBio was the only technology able to (partially) resolve those repeats and their flanks (Figure 4c,d). It is also interesting that linked reads appear to better assemble long repeats such as LTR retrotransposons than short‐read libraries based on different insert sizes (lycPyrSN vs. lycPyrIL; Figure 4b).

The satellite repeat portion of the genome was significantly more represented with PacBio long reads (~9 Mb in lycPyrPB), while neither multiple Illumina libraries nor linked reads could assemble more than 1 Mb of satellites. This is probably due to the highly homogeneous nature of long stretches of satellites that make satellite arrays collapse during assembly (Hartley & O’Neill, 2019). Similar to LTR retrotransposons and rRNA genes, satellites and their flanks are barely assembled in lycPyrIL, lycPyrSN1 and lycPyrSN2. Therefore, satellites are not the most prominent cause of contig fragmentation in Illumina‐based assemblies.

Most copies of LINEs are usually short due to 5′ truncation during integration (Levin & Moran, 2011) and in the paradise crow and other songbirds they seem to be mostly present in old copies (Figure 3e; Suh et al., 2018; Weissensteiner et al., 2019). Therefore, they probably are less homogeneous and often short repeats with more diagnostic mutations, and hence easier to assemble. In fact, both Illumina and 10X Genomics assemblies have 96%–98% of LINEs assembled relative to lycPyr6, and LINEs represent only the fourth causative factor of fragmentation. Finally, we noted a higher number of DNA transposons annotated in the Illumina assemblies (lycPyrIL and lycPyrILPB) compared to the other assemblies. This phenomenon might be explained by annotation issues linked to the fragmentation of those regions or by the presence of unsolved haplotypes. DNA transposons have been inactive in songbirds for even longer than LINEs (Kapusta & Suh, 2017) and should thus be rather straightforward to assemble.

## 5 CONCLUSIONS

Thanks to a manually curated multiplatform assembly and three de novo draft assemblies for the same sample, we were able to characterize and measure genome completeness across different sequencing technologies. As expected, long‐read assemblies are more complete than short‐read assemblies but completeness has been usually measured with statistics that are optimized for short reads rather than for long reads. Scaffold N50 and BUSCO values do not reflect the full potential and strengths of new sequencing technologies, and therefore we measured completeness focusing on the most difficult‐to‐assemble genomic regions. By doing so, we traced the essential steps for generating a high‐quality assembly for a nonmodel organism while optimizing costs and efforts.

**FIGURE 5:**
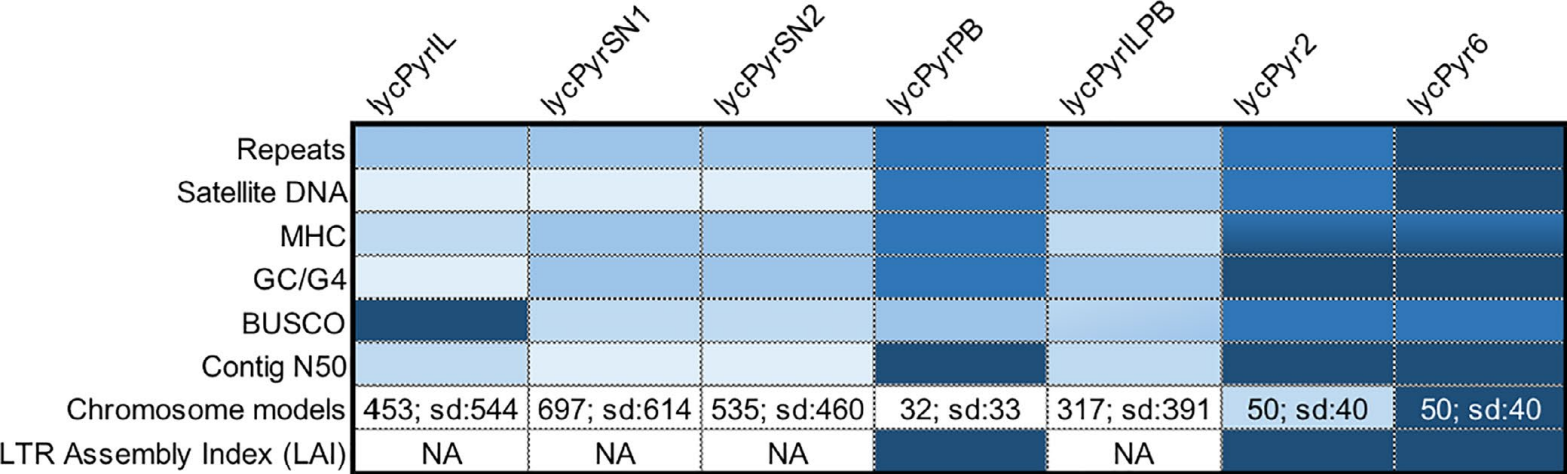
Summary of the relative efficiency of the different technologies using quality and completeness parameters. Dark blue: most effective; light blue: least effective. In the chromosome models row, the mean and standard deviation (*SD*) of the number of contigs corresponding to each chromosome model of the final assembly (lycPyr6) are indicated. More details of the number of contigs for each chromosome models are given in Figure S14. The colour gradient is subjective [Colour figure can be viewed at wileyonlinelibrary.com]

Based on our assembly comparisons, the essential elements to make a chromosome‐level assembly are a contiguous primary assembly based on long reads, an independent set of data for correcting misassemblies (CHiCAGO map or linked reads) and polishing sequencing errors (short and long reads), and a Hi‐C map for chromosome‐level scaffolding. PacBio needs error correction both at the nucleotide level (base calling errors and short indels) and at the assembly level (e.g., chimeric contigs). In our assembly process, approximately one base calling error every 415 bases and ~300,000 short indels were corrected in a single polishing round. Illumina libraries can be used for correcting both nucleotide and assembly errors but a note of caution is due with regard to the polishing step. When polishing the assembly for base calling errors and short indels, short reads might over‐homogenize repetitive sequences and thus it would be advisable to correct only outside repeats. In addition, 10X Genomics linked reads can also be used to correct both sequencing errors and misassemblies (e.g., tigmint, Jackman et al., 2018) and to scaffold the genome (arcs, Yeo et al., 2017, arks, Coombe et al., 2018, fragscaff, Adey et al., 2014). In general, the long‐range information brought by linked reads seems to be very versatile (e.g., assembly correction, scaffolding, structural variation inference, haplotype phasing) and able to better avoid over‐collapsing of repetitive elements and genes (Figures 3 and 4). Therefore, if budgets and sample material are limited, linked reads may be more suitable than short reads alone in obtaining a genomic overview. Nevertheless, long reads provide the most detailed look into difficult‐to‐assemble genomic regions. We summarized the strengths and limitations of the implemented technologies in Figure 5, which can be used as a guide for choosing technologies and ranking assemblies.

We have shown that recent technological developments have led to enormous improvements in assembly quality and completeness, paving the way to more complete comparative genomic analyses, including regions that were previously inaccessible within genomic dark matter. Indeed, important efforts in assembling telomere‐to‐telomere chromosomes are underway thanks to the Telomere‐to‐Telomere (T2T) consortium that recently managed to complete the sequence of a human X chromosome (Miga et al., 2020). At the same time, awareness of technological strengths and weaknesses in resolving repeat‐rich and GC‐rich regions is fundamental in choosing the most suitable technology when designing sequencing projects, and will help in a dilemma many genome scientists now face: choosing the best assembly among many.

## CONFLICT OF INTEREST

Shawn Sullivan and Ivan Liachko are employed at Phase Genomics.

## AUTHORS’ CONTRIBUTIONS

V.P. and A.S. designed the study with input from M.I. and M.P.K.B. V.P. wrote the first draft of the manuscript. V.P. and A.S. wrote subsequent drafts of the manuscript with input from all authors. V.P. assembled and analysed the genome assemblies with input from S.S., I.B. and I.L. L.X. identified the sex‐linked contigs. R.B. analysed the MHC genes. T.H., K.A.J., Q.Z. and M.I. provided samples and data. All authors contributed critically to the drafts and gave final approval for publication.

## Supporting information

Figures S1‐S14

Figure S4

Figure S8

Figure S13

Table S1‐S18

Text S1

## Data Availability

All the raw reads and assemblies can be found on NCBI; accession no.: PRJNA604967. Custom scripts used for generating the results are available on GitHub (https://github.com/ValentinaBoP/lycPyr).

## References

[men13252-bib-0001] Adey, A. , Kitzman, J. O. , Burton, J. N. , Daza, R. , Kumar, A. , Christiansen, L. , Ronaghi, M. , Amini, S. , L. Gunderson, K. , Steemers, F. J. , & Shendure, J. (2014). In vitro, long‐range sequence information for de novo genome assembly via transposase contiguity. Genome Research, 24(12), 2041–2049. 10.1101/gr.178319.114 25327137 PMC4248320

[men13252-bib-0002] Aird, D. , Ross, M. G. , Chen, W.‐S. , Danielsson, M. , Fennell, T. , Russ, C. , Jaffe, D. B. , Nusbaum, C. , & Gnirke, A. (2011). Analyzing and minimizing PCR amplification bias in Illumina sequencing libraries. Genome Biology, 12, 10.1186/gb-2011-12-2-r18 PMC3188800 21338519

[men13252-bib-0003] Alkan, C. , Sajjadian, S. , & Eichler, E. E. (2010). Limitations of next‐generation genome sequence assembly. Nature Methods, 8, 61 10.1038/nmeth.1527 21102452 PMC3115693

[men13252-bib-0004] Altschul, S. F. , Gish, W. , Miller, W. , Myers, E. W. , & Lipman, D. J. (1990). Basic local alignment search tool. Journal of Molecular Biology, 215(3), 403–410. 10.1016/S0022-2836(05)80360-2 2231712

[men13252-bib-0005] Bao, W. , Kojima, K. K. , & Kohany, O. (2015). Repbase update, a database of repetitive elements in eukaryotic genomes. Mobile DNA, 6(1), 11 10.1186/s13100-015-0041-9 26045719 PMC4455052

[men13252-bib-0006] Bellott, D. W. , Skaletsky, H. , Cho, T.‐J. , Brown, L. , Locke, D. , Chen, N. , Galkina, S. , Pyntikova, T. , Koutseva, N. , Graves, T. , Kremitzki, C. , Warren, W. C. , Clark, A. G. , Gaginskaya, E. , Wilson, R. K. , & Page, D. C. (2017). Avian W and mammalian Y chromosomes convergently retained dosage‐sensitive regulators. Nature Genetics, 49, 387–394. 10.1038/ng.3778 28135246 PMC5359078

[men13252-bib-0007] Belser, C. , Istace, B. , Denis, E. , Dubarry, M. , Baurens, F.‐C. , Falentin, C. , Genete, M. , Berrabah, W. , Chèvre, A.‐M. , Delourme, R. , Deniot, G. , Denoeud, F. , Duffé, P. , Engelen, S. , Lemainque, A. , Manzanares‐Dauleux, M. , Martin, G. , Morice, J. , Noel, B. , … Aury, J.‐M. (2018). Chromosome‐scale assemblies of plant genomes using nanopore long reads and optical maps. Nature Plants, 4(11), 879–887. 10.1038/s41477-018-0289-4 30390080

[men13252-bib-0008] Bickhart, D. M. , Rosen, B. D. , Koren, S. , Sayre, B. L. , Hastie, A. R. , Chan, S. , Lee, J. , Lam, E. T. , Liachko, I. , Sullivan, S. T. , Burton, J. N. , Huson, H. J. , Nystrom, J. C. , Kelley, C. M. , Hutchison, J. L. , Zhou, Y. , Sun, J. , Crisà, A. , Ponce de León, F. A. , … Smith, T. P. L. (2017). Single‐molecule sequencing and chromatin conformation capture enable de novo reference assembly of the domestic goat genome. Nature Genetics, 49, 643 10.1038/ng.3802 28263316 PMC5909822

[men13252-bib-0009] Biffi, G. , Tannahill, D. , & Balasubramanian, S. (2012). An Intramolecular G‐Quadruplex structure is required for binding of telomeric repeat‐containing RNA to the telomeric protein TRF2. Journal of the American Chemical Society, 134(29), 11974–11976. 10.1021/ja305734x 22780456 PMC3528108

[men13252-bib-0010] Boman, J. , Frankl‐Vilches, C. , da Silva dos Santos, M. , de Oliveira, E. H. C. , Gahr, M. , & Suh, A. (2019). The Genome of Blue‐Capped Cordon‐Bleu uncovers hidden diversity of LTR retrotransposons in zebra finch. Genes, 10(4), 301 10.3390/genes10040301 PMC6523648 31013951

[men13252-bib-0011] Botero‐Castro, F. , Figuet, E. , Tilak, M.‐K. , Nabholz, B. , & Galtier, N. (2017). Avian genomes revisited: Hidden genes uncovered and the rates versus traits paradox in birds. Molecular Biology and Evolution, 34(12), 3123–3131. 10.1093/molbev/msx236 28962031

[men13252-bib-0012] Bourque, G. , Burns, K. H. , Gehring, M. , Gorbunova, V. , Seluanov, A. , Hammell, M. , Imbeault, M. , Izsvák, Z. , Levin, H. L. , Macfarlan, T. S. , Mager, D. L. , & Feschotte, C. (2018). Ten things you should know about transposable elements. Genome Biology, 19(1), 199 10.1186/s13059-018-1577-z 30454069 PMC6240941

[men13252-bib-0013] Burt, D. W. (2002). Origin and evolution of avian microchromosomes. Cytogenetic and Genome Research, 96(1–4), 97–112. 10.1159/000063018 12438785

[men13252-bib-0014] Burton, J. N. , Adey, A. , Patwardhan, R. P. , Qiu, R. , Kitzman, J. O. , & Shendure, J. (2013). Chromosome‐scale scaffolding of de novo genome assemblies based on chromatin interactions. Nature Biotechnology, 31, 1119 10.1038/nbt.2727 PMC4117202 24185095

[men13252-bib-0015] Butler, J. , MacCallum, I. , Kleber, M. , Shlyakhter, I. A. , Belmonte, M. K. , Lander, E. S. , Nusbaum, C. , & Jaffe, D. B. (2008). allpaths: De novo assembly of whole‐genome shotgun microreads. Genome Research, 18(5), 810–820. 10.1101/gr.7337908 18340039 PMC2336810

[men13252-bib-0016] Cabanettes, F. , & Klopp, C. (2018). d‐genies: Dot plot large genomes in an interactive, efficient and simple way. PeerJ, 6, e4958 10.7717/peerj.4958 29888139 PMC5991294

[men13252-bib-0017] Cantarel, B. L. , Korf, I. , Robb, S. M. , Parra, G. , Ross, E. , Moore, B. , Holt, C. , Sanchez Alvarado, A. , & Yandell, M. (2008). maker: An easy‐to‐use annotation pipeline designed for emerging model organism genomes. Genome Research, 18(1), 188–196. 10.1101/gr.6743907 18025269 PMC2134774

[men13252-bib-0018] Chaisson, M. J. P. , Huddleston, J. , Dennis, M. Y. , Sudmant, P. H. , Malig, M. , Hormozdiari, F. , Antonacci, F. , Surti, U. , Sandstrom, R. , Boitano, M. , Landolin, J. M. , Stamatoyannopoulos, J. A. , Hunkapiller, M. W. , Korlach, J. , & Eichler, E. E. (2014). Resolving the complexity of the human genome using single‐molecule sequencing. Nature, 517, 608 10.1038/nature13907 25383537 PMC4317254

[men13252-bib-0019] Chaisson, M. J. P. , Wilson, R. K. , & Eichler, E. E. (2015). Genetic variation and the de novo assembly of human genomes. Nature Reviews Genetics, 16, 627 10.1038/nrg3933 PMC4745987 26442640

[men13252-bib-0020] Chalopin, D. , Volff, J.‐N. , Galiana, D. , Anderson, J. L. , & Schartl, M. (2015). Transposable elements and early evolution of sex chromosomes in fish. Chromosome Research, 23(3), 545–560. 10.1007/s10577-015-9490-8 26429387

[men13252-bib-0021] Chang, C.‐H. , Chavan, A. , Palladino, J. , Wei, X. , Martins, N. M. C. , Santinello, B. , Chen, C.‐C. , Erceg, J. , Beliveau, B. J. , Wu, C.‐T. , Larracuente, A. M. , & Mellone, B. G. (2019). Islands of retroelements are major components of *Drosophila* centromeres. PLOS Biology, 17(5), e3000241 10.1371/journal.pbio.3000241 31086362 PMC6516634

[men13252-bib-0022] Charlesworth, B. , Harvey, P. H. , Charlesworth, B. , & Charlesworth, D. (2000). The degeneration of Y chromosomes. Philosophical Transactions of the Royal Society of London. Series B: Biological Sciences, 355(1403), 1563–1572. 10.1098/rstb.2000.0717 11127901 PMC1692900

[men13252-bib-0023] Chin, C.‐S. , Peluso, P. , Sedlazeck, F. J. , Nattestad, M. , Concepcion, G. T. , Clum, A. , Dunn, C. , O'Malley, R. , Figueroa‐Balderas, R. , Morales‐Cruz, A. , Cramer, G. R. , Delledonne, M. , Luo, C. , Ecker, J. R. , Cantu, D. , Rank, D. R. , & Schatz, M. C. (2016). Phased diploid genome assembly with single‐molecule real‐time sequencing. Nature Methods, 13, 1050 10.1038/nmeth.4035 27749838 PMC5503144

[men13252-bib-0024] Chuong, E. B. , Elde, N. C. , & Feschotte, C. (2016). Regulatory activities of transposable elements: From conflicts to benefits. Nature Reviews Genetics, 18, 71 10.1038/nrg.2016.139 PMC5498291 27867194

[men13252-bib-0025] Coombe, L. , Zhang, J. , Vandervalk, B. P. , Chu, J. , Jackman, S. D. , Birol, I. , & Warren, R. L. (2018). arks: Chromosome‐scale scaffolding of human genome drafts with linked read kmers. BMC Bioinformatics, 19(1), 234 10.1186/s12859-018-2243-x 29925315 PMC6011487

[men13252-bib-0026] Cowley, M. , & Oakey, R. J. (2013). Transposable elements re‐wire and fine‐tune the transcriptome. PLOS Genetics, 9(1), e1003234 10.1371/journal.pgen.1003234 23358118 PMC3554611

[men13252-bib-0027] Christidis, L. (1990). Aves (Vol. 4). Balogh Scientific Books.

[men13252-bib-0028] Deakin, J. E. , Potter, S. , O’Neill, R. , Ruiz‐Herrera, A. , Cioffi, M. B. , Eldridge, M. D. B. , Fukui, K. , Marshall Graves, J. A. , Griffin, D. , Grutzner, F. , Kratochvíl, L. , Miura, I. , Rovatsos, M. , Srikulnath, K. , Wapstra, E. , & Ezaz, T. (2019). Chromosomics: Bridging the gap between genomes and chromosomes. Genes, 10, 627 10.3390/genes10080627 PMC6723020 31434289

[men13252-bib-0029] Deamer, D. , Akeson, M. , & Branton, D. (2016). Three decades of nanopore sequencing. Nature Biotechnology, 34(5), 518–524. 10.1038/nbt.3423 PMC6733523 27153285

[men13252-bib-0030] Deschamps, S. , Zhang, Y. , Llaca, V. , Ye, L. , Sanyal, A. , King, M. , May, G. , & Lin, H. (2018). A chromosome‐scale assembly of the sorghum genome using nanopore sequencing and optical mapping. Nature Communications, 9(1), 4844 10.1038/s41467-018-07271-1 PMC6242865 30451840

[men13252-bib-0031] Dohm, J. C. , Lottaz, C. , Borodina, T. , & Himmelbauer, H. (2008). Substantial biases in ultra‐short read data sets from high‐throughput DNA sequencing. Nucleic Acids Research, 36, e105 10.1093/nar/gkn425 18660515 PMC2532726

[men13252-bib-0032] Domanska, D. , Kanduri, C. , Simovski, B. , & Sandve, G. K. (2018). Mind the gaps: Overlooking inaccessible regions confounds statistical testing in genome analysis. BMC Bioinformatics, 19(1), 481 10.1186/s12859-018-2438-1 30547739 PMC6293655

[men13252-bib-0033] Dréau, A. , Venu, V. , Avdievich, E. , Gaspar, L. , & Jones, F. C. (2019). Genome‐wide recombination map construction from single individuals using linked‐read sequencing. Nature Communications, 10(1), 4309 10.1038/s41467-019-12210-9 PMC6754380 31541091

[men13252-bib-0034] Du, Z. , Zhao, Y. , & Li, N. (2008). Genome‐wide analysis reveals regulatory role of G4 DNA in gene transcription. Genome Research, 18(2), 233–241. 10.1101/gr.6905408 18096746 PMC2203621

[men13252-bib-0035] Du, Z. , Zhao, Y. , & Li, N. (2009). Genome‐wide colonization of gene regulatory elements by G4 DNA motifs. Nucleic Acids Research, 37(20), 6784–6798. 10.1093/nar/gkp710 19759215 PMC2777415

[men13252-bib-0036] Dudchenko, O. , Batra, S. S. , Omer, A. D. , Nyquist, S. K. , Hoeger, M. , Durand, N. C. , & Aiden, E. L. (2017). De novo assembly of the *Aedes aegypti* genome using Hi‐C yields chromosome‐length scaffolds. Science, 356(6333), 92–95. 10.1126/science.aal3327 28336562 PMC5635820

[men13252-bib-0037] Dudchenko, O. , Shamim, M. S. , Batra, S. S. , Durand, N. C. , Musial, N. T. , Mostofa, R. , Pham, M. , St Hilaire, B. G. , Yao, W. , Stamenova, E. , Hoeger, M. , Nyquist, S. K. , Korchina, V. , Pletch, K. , Flanagan, J. P. , Tomaszewicz, A. , McAloose, D. , Estrada, C. P. , Novak, B. J. , … Aiden, E. L. (2018). The Juicebox Assembly Tools module facilitates de novo assembly of mammalian genomes with chromosome‐length scaffolds for under $1000. bioRxiv, 254797 10.1101/254797

[men13252-bib-0038] Durand, N. C. , Shamim, M. S. , Machol, I. , Rao, S. S. P. , Huntley, M. H. , Lander, E. S. , & Aiden, E. L. (2016). Juicer provides a one‐click system for analyzing loop‐resolution Hi‐C experiments. Cell Systems, 3(1), 95–98. 10.1016/j.cels.2016.07.002 27467249 PMC5846465

[men13252-bib-0039] Eid, J. , Fehr, A. , Gray, J. , Luong, K. , Lyle, J. , Otto, G. , Peluso, P. , Rank, D. , Baybayan, P. , Bettman, B. , Bibillo, A. , Bjornson, K. , Chaudhuri, B. , Christians, F. , Cicero, R. , Clark, S. , Dalal, R. , deWinter, A. , Dixon, J. , … Turner, S. (2009). Real‐time DNA sequencing from single polymerase molecules. Science, 323(5910), 133–138. 10.1126/science.1162986 19023044

[men13252-bib-0040] Emera, D. , & Wagner, G. P. (2012). Transposable element recruitments in the mammalian placenta: Impacts and mechanisms. Briefings in Functional Genomics, 11(4), 267–276. 10.1093/bfgp/els013 22753775

[men13252-bib-0041] English, A. C. , Richards, S. , Han, Y. I. , Wang, M. , Vee, V. , Qu, J. , Qin, X. , Muzny, D. M. , Reid, J. G. , Worley, K. C. , & Gibbs, R. A. (2012). Mind the Gap: Upgrading genomes with pacific biosciences RS long‐read sequencing technology. PLoS One, 7(11), e47768 10.1371/journal.pone.0047768 23185243 PMC3504050

[men13252-bib-0042] Etherington, G. J. , Heavens, D. , Baker, D. , Lister, A. , McNelly, R. , Garcia, G. , Clavijo, B. , Macaulay, I. , Haerty, W. , & Di Palma, F. (2019). Sequencing smart: De novo sequencing and assembly approaches for non‐model mammals, bioRxiv, 723890 10.1101/723890 PMC7216774 32396200

[men13252-bib-0043] Faino, L. , Seidl, M. F. , Datema, E. , van den Berg, G. C. M. , Janssen, A. , Wittenberg, A. H. J. , & Thomma, B. P. H. J. (2015). Single‐molecule real‐time sequencing combined with optical mapping yields completely finished fungal genome. Mbio, 6(4), e00936–00915 10.1128/mBio.00936-15 26286689 PMC4542186

[men13252-bib-0044] Goebel, J. , Promerová, M. , Bonadonna, F. , McCoy, K. D. , Serbielle, C. , Strandh, M. , Yannic, G. , Burri, R. , & Fumagalli, L. (2017). 100 million years of multigene family evolution: Origin and evolution of the avian MHC class IIB. BMC Genomics, 18(1), 460 10.1186/s12864-017-3839-7 28610613 PMC5470263

[men13252-bib-0045] Goodwin, S. , McPherson, J. D. , & McCombie, W. R. (2016). Coming of age: Ten years of next‐generation sequencing technologies. Nature Reviews Genetics, 17, 333–351. 10.1038/nrg.2016.49 PMC10373632 27184599

[men13252-bib-0046] Gordon, D. , Huddleston, J. , Chaisson, M. J. P. , Hill, C. M. , Kronenberg, Z. N. , Munson, K. M. , Malig, M. , Raja, A. , Fiddes, I. , Hillier, L. W. , Dunn, C. , Baker, C. , Armstrong, J. , Diekhans, M. , Paten, B. , Shendure, J. , Wilson, R. K. , Haussler, D. , Chin, C.‐S. , & Eichler, E. E. (2016). Long‐read sequence assembly of the gorilla genome. Science, 352(6281), aae0344 10.1126/science.aae0344 27034376 PMC4920363

[men13252-bib-0047] Gregory, T. R. (2019). Animal genome size database. Retrieved from http://www.genomesize.com

[men13252-bib-0048] Griffin, D. , & Burt, D. W. (2014). All chromosomes great and small: 10 years on. Chromosome Research, 22(1), 1–6. 10.1007/s10577-014-9413-0 24700106

[men13252-bib-0049] Guiblet, W. M. , Cremona, M. A. , Cechova, M. , Harris, R. S. , Kejnovská, I. , Kejnovsky, E. , Eckert, K. , Chiaromonte, F. , & Makova, K. D. (2018). Long‐read sequencing technology indicates genome‐wide effects of non‐B DNA on polymerization speed and error rate. Genome Research, 28(12), 1767–1778. 10.1101/gr.241257.118 30401733 PMC6280752

[men13252-bib-0050] Haas, B. J. , Papanicolaou, A. , Yassour, M. , Grabherr, M. , Blood, P. D. , Bowden, J. , Couger, M. B. , Eccles, D. , Li, B. O. , Lieber, M. , MacManes, M. D. , Ott, M. , Orvis, J. , Pochet, N. , Strozzi, F. , Weeks, N. , Westerman, R. , William, T. , Dewey, C. N. , … Regev, A. (2013). De novo transcript sequence reconstruction from RNA‐seq using the Trinity platform for reference generation and analysis. Nature Protocols, 8(8), 1494–1512. 10.1038/nprot.2013.084 23845962 PMC3875132

[men13252-bib-0051] Hartley, G. , & O’Neill, R. J. (2019). Centromere repeats: Hidden gems of the genome. Genes, 10(3), 223 10.3390/genes10030223 PMC6471113 30884847

[men13252-bib-0052] Hobza, R. , Cegan, R. , Jesionek, W. , Kejnovsky, E. , Vyskot, B. , & Kubat, Z. (2017). Impact of repetitive elements on the Y chromosome formation in plants. Genes (Basel), 8(11), 10.3390/genes8110302 PMC5704215 29104214

[men13252-bib-0053] Hron, T. , Pajer, P. , Pačes, J. , Bartůněk, P. , & Elleder, D. (2015). Hidden genes in birds. Genome Biology, 16(1), 164 10.1186/s13059-015-0724-z 26283656 PMC4539667

[men13252-bib-0054] Hughes, A. L. , & Yeager, M. (1998). Natural selection at major histocompatibility complex loci of vertebrates. Annual Review of Genetics, 32(1), 415–435. 10.1146/annurev.genet.32.1.415 9928486

[men13252-bib-0055] Irestedt, M. , Jønsson, K. A. , Fjeldså, J. , Christidis, L. , & Ericson, P. G. P. (2009). An unexpectedly long history of sexual selection in birds‐of‐paradise. BMC Evolutionary Biology, 9(1), 235 10.1186/1471-2148-9-235 19758445 PMC2755009

[men13252-bib-0056] Jackman, S. D. , Coombe, L. , Chu, J. , Warren, R. L. , Vandervalk, B. P. , Yeo, S. , Xue, Z. , Mohamadi, H. , Bohlmann, J. , Jones, S. J. M. , & Birol, I. (2018). tigmint: Correcting assembly errors using linked reads from large molecules. BMC Bioinformatics, 19(1), 393 10.1186/s12859-018-2425-6 30367597 PMC6204047

[men13252-bib-0057] Jain, M. , Olsen, H. E. , Turner, D. J. , Stoddart, D. , Bulazel, K. V. , Paten, B. , Haussler, D. , Willard, H. F. , Akeson, M. , & Miga, K. H. (2018). Linear assembly of a human centromere on the Y chromosome. Nature Biotechnology, 36, 321 10.1038/nbt.4109 PMC5886786 29553574

[men13252-bib-0058] Johnson, J. M. , Edwards, S. , Shoemaker, D. , & Schadt, E. E. (2005). Dark matter in the genome: Evidence of widespread transcription detected by microarray tiling experiments. Trends in Genetics, 21(2), 93–102. 10.1016/j.tig.2004.12.009 15661355

[men13252-bib-0059] Kapitonov, V. V. , & Koonin, E. V. (2015). Evolution of the RAG1‐RAG2 locus: Both proteins came from the same transposon. Biology Direct, 10(1), 20 10.1186/s13062-015-0055-8 25928409 PMC4411706

[men13252-bib-0060] Kapusta, A. , & Suh, A. (2017). Evolution of bird genomes‐a transposon's‐eye view. Annals of the New York Academy of Sciences, 1389(1), 164–185. 10.1111/nyas.13295 27997700

[men13252-bib-0061] Korf, I. (2004). Gene finding in novel genomes. BMC Bioinformatics, 5(1), 59 10.1186/1471-2105-5-59 15144565 PMC421630

[men13252-bib-0062] Kozarewa, I. , Ning, Z. , Quail, M. A. , Sanders, M. J. , Berriman, M. , & Turner, D. J. (2009). Amplification‐free Illumina sequencing‐library preparation facilitates improved mapping and assembly of (G+C)‐biased genomes. Nature Methods, 6, 291–295. 10.1038/nmeth.1311 19287394 PMC2664327

[men13252-bib-0063] Kurtz, S. , Phillippy, A. , Delcher, A. L. , Smoot, M. , Shumway, M. , Antonescu, C. , & Salzberg, S. L. (2004). Versatile and open software for comparing large genomes. Genome Biology, 5(2), R12 10.1186/gb-2004-5-2-r12 14759262 PMC395750

[men13252-bib-0064] Lajoie, B. R. , Dekker, J. , & Kaplan, N. (2015). The Hitchhiker's guide to Hi‐C analysis: Practical guidelines. Methods, 72, 65–75. 10.1016/j.ymeth.2014.10.031 25448293 PMC4347522

[men13252-bib-0065] Law, J. A. , & Jacobsen, S. E. (2010). Establishing, maintaining and modifying DNA methylation patterns in plants and animals. Nature Reviews Genetics, 11(3), 204–220. 10.1038/nrg2719 PMC3034103 20142834

[men13252-bib-0066] Lerat, E. , Casacuberta, J. , Chaparro, C. , & Vieira, C. (2019). On the importance to acknowledge transposable elements in epigenomic analyses. Genes, 10(4), 258 10.3390/genes10040258 PMC6523952 30935103

[men13252-bib-0067] Levin, H. L. , & Moran, J. V. (2011). Dynamic interactions between transposable elements and their hosts. Nature Reviews Genetics, 12(9), 615–627. 10.1038/nrg3030 PMC3192332 21850042

[men13252-bib-0068] Levis, R. W. , Ganesan, R. , Houtchens, K. , Tolar, L. A. , & Sheen, F.‐M. (1993). Transposons in place of telomeric repeats at a *Drosophila* telomere. Cell, 75(6), 1083–1093. 10.1016/0092-8674(93)90318-K 8261510

[men13252-bib-0069] Li, H. , & Durbin, R. (2010). Fast and accurate long‐read alignment with Burrows‐Wheeler transform. Bioinformatics, 26(5), 589–595. 10.1093/bioinformatics/btp698 20080505 PMC2828108

[men13252-bib-0070] Li, H. , Handsaker, B. , Wysoker, A. , Fennell, T. , Ruan, J. , Homer, N. , Marth, G. , Abecasis, G. , Durbin, R. , & 1000 Genome Project Data Processing Subgroup (2009). The Sequence Alignment/Map format and SAMtools. Bioinformatics, 25(16), 2078–2079. 10.1093/bioinformatics/btp352 19505943 PMC2723002

[men13252-bib-0071] Li, Q. , Li, H. , Huang, W. U. , Xu, Y. , Zhou, Q. , Wang, S. , Ruan, J. , Huang, S. , & Zhang, Z. (2019). A chromosome‐scale genome assembly of cucumber (*Cucumis sativus* L.). GigaScience, 8(6), 10 10.1093/gigascience/giz072 PMC6582320 31216035

[men13252-bib-0072] Lieberman‐Aiden, E. , van Berkum, N. L. , Williams, L. , Imakaev, M. , Ragoczy, T. , Telling, A. , Amit, I. , Lajoie, B. R. , Sabo, P. J. , Dorschner, M. O. , Sandstrom, R. , Bernstein, B. , Bender, M. A. , Groudine, M. , Gnirke, A. , Stamatoyannopoulos, J. , Mirny, L. A. , Lander, E. S. , & Dekker, J. (2009). Comprehensive mapping of long‐range interactions reveals folding principles of the human genome. Science, 326(5950), 289–293. 10.1126/science.1181369 19815776 PMC2858594

[men13252-bib-0073] Ligon, R. A. , Diaz, C. D. , Morano, J. L. , Troscianko, J. , Stevens, M. , Moskeland, A. , Laman, T. G. , & Scholes III, E. (2018). Evolution of correlated complexity in the radically different courtship signals of birds‐of‐paradise. PLOS Biology, 16(11), e2006962 10.1371/journal.pbio.2006962 30457985 PMC6245505

[men13252-bib-0074] Loomis, E. W. , Eid, J. S. , Peluso, P. , Yin, J. , Hickey, L. , Rank, D. , McCalmon, S. , Hagerman, R. J. , Tassone, F. , & Hagerman, P. J. (2013). Sequencing the unsequenceable: Expanded CGG‐repeat alleles of the fragile X gene. Genome Research, 23(1), 121–128. 10.1101/gr.141705.112 23064752 PMC3530672

[men13252-bib-0075] Lovell, P. V. , Wirthlin, M. , Wilhelm, L. , Minx, P. , Lazar, N. H. , Carbone, L. , Warren, W. C. , & Mello, C. V. (2014). Conserved syntenic clusters of protein coding genes are missing in birds. Genome Biology, 15(12), 565 10.1186/s13059-014-0565-1 25518852 PMC4290089

[men13252-bib-0076] Lowe, T. M. , & Eddy, S. R. (1997). trnascan‐se: A program for improved detection of transfer RNA genes in genomic sequence. Nucleic Acids Research, 25(5), 955–964. 10.1093/nar/25.5.955 9023104 PMC146525

[men13252-bib-0077] Maizels, N. , & Gray, L. T. (2013). The G4 genome. PLOS Genetics, 9(4), e1003468 10.1371/journal.pgen.1003468 23637633 PMC3630100

[men13252-bib-0078] Marks, P. , Garcia, S. , Barrio, A. M. , Belhocine, K. , Bernate, J. , Bharadwaj, R. , Bjornson, K. , Catalanotti, C. , Delaney, J. , Fehr, A. , Fiddes, I. T. , Galvin, B. , Heaton, H. , Herschleb, J. , Hindson, C. , Holt, E. , Jabara, C. B. , Jett, S. , Keivanfar, N. , … Church, D. M. (2019). Resolving the full spectrum of human genome variation using Linked‐Reads. Genome Research, 29(4), 635–645. 10.1101/gr.234443.118 30894395 PMC6442396

[men13252-bib-0079] McGurk, M. P. , Dion‐Côté, A.‐M. , & Barbash, D. A. (2019). Rapid evolution at the telomere: Transposable element dynamics at an intrinsically unstable locus. bioRxiv, 782904 10.1101/782904 PMC8045721 33724410

[men13252-bib-0080] Meyne, J. , Ratliff, R. L. , & Moyzis, R. K. (1989). Conservation of the human telomere sequence (TTAGGG)n among vertebrates. Proceedings of the National Academy of Sciences, 86(18), 7049–7053. 10.1073/pnas.86.18.7049 PMC297991 2780561

[men13252-bib-0081] Michael, T. P. , Jupe, F. , Bemm, F. , Motley, S. T. , Sandoval, J. P. , Lanz, C. , Loudet, O. , Weigel, D. , & Ecker, J. R. (2018). High contiguity *Arabidopsis thaliana* genome assembly with a single nanopore flow cell. Nature Communications, 9(1), 541 10.1038/s41467-018-03016-2 PMC5803254 29416032

[men13252-bib-0082] Miga, K. H. , Koren, S. , Rhie, A. , Vollger, M. R. , Gershman, A. , Bzikadze, A. , Brooks, S. , Howe, E. , Porubsky, D. , Logsdon, G. A. , Schneider, V. A. , Potapova, T. , Wood, J. , Chow, W. , Armstrong, J. , Fredrickson, J. , Pak, E. , Tigyi, K. , Kremitzki, M. , … Phillippy, A. M. (2020). Telomere‐to‐telomere assembly of a complete human X chromosome. Nature, 585(7823), 79–84. 10.1038/s41586-020-2547-7 32663838 PMC7484160

[men13252-bib-0083] Miller, M. M. , & Taylor, R. L. Jr (2016). Brief review of the chicken Major Histocompatibility Complex: The genes, their distribution on chromosome 16, and their contributions to disease resistance. Poultry Science, 95(2), 375–392. 10.3382/ps/pev379 PMC4988538 26740135

[men13252-bib-0084] Montoliu‐Nerin, M. , Sánchez‐García, M. , Bergin, C. , Grabherr, M. , Ellis, B. , Kutschera, V. E. , Kierczak, M. , Johannesson, H. , & Rosling, A. (2020). Building de novo reference genome assemblies of complex eukaryotic microorganisms from single nuclei. Scientific Reports, 10(1), 1303 10.1038/s41598-020-58025-3 31992756 PMC6987183

[men13252-bib-0085] Nishimura, O. , Hara, Y. , & Kuraku, S. (2017). gVolante for standardizing completeness assessment of genome and transcriptome assemblies. Bioinformatics, 33(22), 3635–3637. 10.1093/bioinformatics/btx445 29036533 PMC5870689

[men13252-bib-0086] O’Connor, E. A. , Westerdahl, H. , Burri, R. , & Edwards, S. V. (2019). Avian MHC evolution in the era of genomics: Phase 1.0. Cells, 8(10), 1152 10.3390/cells8101152 PMC6829271 31561531

[men13252-bib-0087] O'Leary, N. A. , Wright, M. W. , Brister, J. R. , Ciufo, S. , Haddad, D. , McVeigh, R. , Rajput, B. , Robbertse, B. , Smith‐White, B. , Ako‐Adjei, D. , Astashyn, A. , Badretdin, A. , Bao, Y. , Blinkova, O. , Brover, V. , Chetvernin, V. , Choi, J. , Cox, E. , Ermolaeva, O. , … Pruitt, K. D. (2015). Reference sequence (RefSeq) database at NCBI: Current status, taxonomic expansion, and functional annotation. Nucleic Acids Research, 44(D1), D733–D745. 10.1093/nar/gkv1189 26553804 PMC4702849

[men13252-bib-0088] Ooi, H. S. , Kwo, C. Y. , Wildpaner, M. , Sirota, F. L. , Eisenhaber, B. , Maurer‐Stroh, S. , Wong, W. C. , Schleiffer, A. , Eisenhaber, F. , & Schneider, G. (2009). annie: Integrated de novo protein sequence annotation. Nucleic Acids Research, 37(Web Server), W435–W440. 10.1093/nar/gkp254 19389726 PMC2703921

[men13252-bib-0089] Ou, S. , Chen, J. , & Jiang, N. (2018). Assessing genome assembly quality using the LTR Assembly Index (LAI). Nucleic Acids Research, 46(21), e126 10.1093/nar/gky730 30107434 PMC6265445

[men13252-bib-0090] Oyola, S. O. , Otto, T. D. , Gu, Y. , Maslen, G. , Manske, M. , Campino, S. , Turner, D. J. , MacInnis, B. , Kwiatkowski, D. P. , Swerdlow, H. P. , & Quail, M. A. (2012). Optimizing illumina next‐generation sequencing library preparation for extremely at‐biased genomes. BMC Genomics, 13(1), 1 10.1186/1471-2164-13-1 22214261 PMC3312816

[men13252-bib-0091] Paajanen, P. , Kettleborough, G. , López‐Girona, E. , Giolai, M. , Heavens, D. , Baker, D. , Lister, A. , Cugliandolo, F. , Wilde, G. , Hein, I. , Macaulay, I. , Bryan, G. J. , & Clark, M. D. (2019). A critical comparison of technologies for a plant genome sequencing project. GigaScience, 8(3), 1–12. 10.1093/gigascience/giy163 PMC6423373 30624602

[men13252-bib-0092] Peñalba, J. V. , Deng, Y. , Fang, Q. , Joseph, L. , Moritz, C. , & Cockburn, A. (2019). Genome of an iconic Australian bird: High‐quality assembly and linkage map of the superb fairy‐wren (*Malurus cyaneus*). Molecular Ecology Resources, 20(2), 560–578. 10.1111/1755-0998.13124 31821695

[men13252-bib-0093] Peona, V. , Palacios‐Gimenez, O. M. , Blommaert, J. , Liu, J. , Haryoko, T. , Jønsson, K. A. , Suh, A. (2020) The avian W chromosome is a refugium for endogenous retroviruses with likely effects on female‐biased mutational load and genetic incompatibilities. BioRxiv, 2020.07.31.230854 10.1101/2020.07.31.230854 PMC8310711 34304594

[men13252-bib-0094] Peona, V. , Weissensteiner, M. H. , & Suh, A. (2018). How complete are "complete" genome assemblies?‐An avian perspective. Molecular Ecology Resources, 18(6), 1188–1195. 10.1111/1755-0998.12933 30035372

[men13252-bib-0095] Pertea, M. , Pertea, G. M. , Antonescu, C. M. , Chang, T.‐C. , Mendell, J. T. , & Salzberg, S. L. (2015). StringTie enables improved reconstruction of a transcriptome from RNA‐seq reads. Nature Biotechnology, 33(3), 290–295. 10.1038/nbt.3122 PMC4643835 25690850

[men13252-bib-0096] Pettersson, M. E. , Rochus, C. M. , Han, F. , Chen, J. , Hill, J. , Wallerman, O. , Fan, G. , Hong, X. , Xu, Q. , Zhang, H. E. , Liu, S. , Liu, X. , Haggerty, L. , Hunt, T. , Martin, F. J. , Flicek, P. , Bunikis, I. , Folkvord, A. , & Andersson, L. (2019). A chromosome‐level assembly of the Atlantic herring genome—Detection of a supergene and other signals of selection. Genome Research, 29(11), 1919–1928. 10.1101/gr.253435.119 31649060 PMC6836730

[men13252-bib-0097] Platt, R. N. II , Blanco‐Berdugo, L. , & Ray, D. A. (2016). Accurate transposable element annotation is vital when analyzing new genome assemblies. Genome Biology and Evolution, 8(2), 403–410. 10.1093/gbe/evw009 26802115 PMC4779615

[men13252-bib-0098] Prost, S. , Armstrong, E. E. , Nylander, J. , Thomas, G. W. C. , Suh, A. , Petersen, B. , Dalen, L. , Benz, B. W. , Blom, M. P. K. , Palkopoulou, E. , Ericson, P. G. P. , & Irestedt, M. (2019). Comparative analyses identify genomic features potentially involved in the evolution of birds‐of‐paradise. GigaScience, 8, 1–12. 10.1093/gigascience/giz003 PMC6497032 30689847

[men13252-bib-0099] Putnam, N. H. , O'Connell, B. L. , Stites, J. C. , Rice, B. J. , Blanchette, M. , Calef, R. , Troll, C. J. , Fields, A. , Hartley, P. D. , Sugnet, C. W. , Haussler, D. , Rokhsar, D. S. , & Green, R. E. (2016). Chromosome‐scale shotgun assembly using an in vitro method for long‐range linkage. Genome Research, 26(3), 342–350. 10.1101/gr.193474.115 26848124 PMC4772016

[men13252-bib-0100] Quevillon, E. , Silventoinen, V. , Pillai, S. , Harte, N. , Mulder, N. , Apweiler, R. , & Lopez, R. (2005). interproscan: Protein domains identifier. Nucleic Acids Research, 33(suppl_2), W116–W120. 10.1093/nar/gki442 15980438 PMC1160203

[men13252-bib-0101] Quinlan, A. R. (2014). bedtools: The swiss‐army tool for genome feature analysis. Current Protocols in Bioinformatics, 47(1), 11.12.11–11.12.34. 10.1002/0471250953.bi1112s47 PMC4213956 25199790

[men13252-bib-0102] Raiber, E.‐A. , Kranaster, R. , Lam, E. , Nikan, M. , & Balasubramanian, S. (2011). A non‐canonical DNA structure is a binding motif for the transcription factor SP1 in vitro. Nucleic Acids Research, 40(4), 1499–1508. 10.1093/nar/gkr882 22021377 PMC3287196

[men13252-bib-0103] Rhie, A. , McCarthy, S. , Fedrigo, O. , Damas, J , Formenti, G. , Koren, S , Uliano‐Silva, M. , Chow, W. , Fungtammasan, A. , Gedman, G. L. , Cantin, L. , Thibaud‐Nissen, F. , Haggerty, L. , Lee, C. , June Ko, B. , Kim, J. , Bista, I. , Smith, M. , Haase, B. , … Jarvis, E. D. (2020). Towards complete and error‐free genome assemblies of all vertebrate species. bioRxiv, 2020.09.08.285395 10.1101/2020.09.08.285395 PMC8081667 33911273

[men13252-bib-0104] Rhoads, A. , & Au, K. F. (2015). PacBio sequencing and its applications. Genomics, Proteomics and Bioinformatics, 13(5), 278–289. 10.1016/j.gpb.2015.08.002 PMC4678779 26542840

[men13252-bib-0105] Ribeiro, F. J. , Przybylski, D. , Yin, S. , Sharpe, T. , Gnerre, S. , Abouelleil, A. , Berlin, A. M. , Montmayeur, A. , Shea, T. P. , Walker, B. J. , Young, S. K. , Russ, C. , Nusbaum, C. , MacCallum, I. , & Jaffe, D. B. (2012). Finished bacterial genomes from shotgun sequence data. Genome Research, 22(11), 2270–2277. 10.1101/gr.141515.112 22829535 PMC3483556

[men13252-bib-0106] Sahakyan, A. B. , Chambers, V. S. , Marsico, G. , Santner, T. , Di Antonio, M. , & Balasubramanian, S. (2017). Machine learning model for sequence‐driven DNA G‐quadruplex formation. Scientific Reports, 7(1), 14535 10.1038/s41598-017-14017-4 29109402 PMC5673958

[men13252-bib-0107] Schadt, E. E. , Turner, S. , & Kasarskis, A. (2010). A window into third‐generation sequencing. Human Molecular Genetics, 19(R2), R227–R240. 10.1093/hmg/ddq416 20858600

[men13252-bib-0108] Schiavone, D. , Guilbaud, G. , Murat, P. , Papadopoulou, C. , Sarkies, P. , Prioleau, M.‐N. , Balasubramanian, S. , & Sale, J. E. (2014). Determinants of G quadruplex‐induced epigenetic instability in REV1‐deficient cells. The EMBO Journal, 33(21), 2507–2520. 10.15252/embj.201488398 25190518 PMC4282387

[men13252-bib-0109] Schmidt, M.‐ H.‐W. , Vogel, A. , Denton, A. K. , Istace, B. , Wormit, A. , van de Geest, H. , Usadel, B. (2017). De novo assembly of a new *Solanum pennellii* accession using nanopore sequencing. The Plant Cell, 29(10), 2336–2348. 10.1105/tpc.17.00521 29025960 PMC5774570

[men13252-bib-0110] Sedlazeck, F. J. , Lee, H. , Darby, C. A. , & Schatz, M. C. (2018). Piercing the dark matter: Bioinformatics of long‐range sequencing and mapping. Nature Reviews Genetics, 19(6), 329–346. 10.1038/s41576-018-0003-4 29599501

[men13252-bib-0111] Seo, J.‐S. , Rhie, A. , Kim, J. , Lee, S. , Sohn, M.‐H. , Kim, C.‐U. , Hastie, A. , Cao, H. , Yun, J.‐Y. , Kim, J. , Kuk, J. , Park, G. H. , Kim, J. , Ryu, H. , Kim, J. , Roh, M. , Baek, J. , Hunkapiller, M. W. , Korlach, J. , … Kim, C. (2016). De novo assembly and phasing of a Korean human genome. Nature, 538, 243–247. 10.1038/nature20098 27706134

[men13252-bib-0112] Shedlock, A. M. , Takahashi, K. , & Okada, N. (2004). SINEs of speciation: Tracking lineages with retroposons. Trends in Ecology and Evolution, 19(10), 545–553. 10.1016/j.tree.2004.08.002 16701320

[men13252-bib-0113] Shiina, T. , Hosomichi, K. , Inoko, H. , & Kulski, J. K. (2009). The HLA genomic loci map: Expression, interaction, diversity and disease. Journal of Human Genetics, 54, 15 10.1038/jhg.2008.5 19158813

[men13252-bib-0114] Simão, F. A. , Waterhouse, R. M. , Ioannidis, P. , Kriventseva, E. V. , & Zdobnov, E. M. (2015). busco: Assessing genome assembly and annotation completeness with single‐copy orthologs. Bioinformatics, 31(19), 3210–3212. 10.1093/bioinformatics/btv351 26059717

[men13252-bib-0115] Slater, G. S. C. , & Birney, E. (2005). Automated generation of heuristics for biological sequence comparison. BMC Bioinformatics, 6(1), 31 10.1186/1471-2105-6-31 15713233 PMC553969

[men13252-bib-0116] Slotkin, R. K. (2018). The case for not masking away repetitive DNA. Mobile DNA, 9(1), 15 10.1186/s13100-018-0120-9 29743957 PMC5930866

[men13252-bib-0117] Smeds, L. , Kawakami, T. , Burri, R. , Bolivar, P. , Husby, A. , Qvarnström, A. , Uebbing, S. , & Ellegren, H. (2014). Genomic identification and characterization of the pseudoautosomal region in highly differentiated avian sex chromosomes. Nature Communications, 5(1), 5448 10.1038/ncomms6448 PMC4272252 25378102

[men13252-bib-0118] Smeds, L. , Warmuth, V. , Bolivar, P. , Uebbing, S. , Burri, R. , Suh, A. , Nater, A. , Bureš, S. , Garamszegi, L. Z. , Hogner, S. , Moreno, J. , Qvarnström, A. , Ružić, M. , Sæther, S.‐A. , Sætre, G.‐P. , Török, J. , & Ellegren, H. (2015). Evolutionary analysis of the female‐specific avian W chromosome. Nature Communications, 6(1), 7330 10.1038/ncomms8330 PMC4468903 26040272

[men13252-bib-0119] Smith, J. J. , Timoshevskaya, N. , Timoshevskiy, V. A. , Keinath, M. C. , Hardy, D. , & Voss, S. R. (2019). A chromosome‐scale assembly of the axolotl genome. Genome Research, 29(2), 317–324. 10.1101/gr.241901.118 30679309 PMC6360810

[men13252-bib-0120] Stanke, M. , Keller, O. , Gunduz, I. , Hayes, A. , Waack, S. , & Morgenstern, B. (2006). augustus: Ab initio prediction of alternative transcripts. Nucleic Acids Research, 34(suppl_2), W435–W439. 10.1093/nar/gkl200 16845043 PMC1538822

[men13252-bib-0121] Su, X.‐Z. , Wu, Y. , Sifri, C. D. , & Wellems, T. E. (1996). Reduced extension temperatures required for PCR amplification of extremely A+T‐rich DNA. Nucleic Acids Research, 24(8), 1574–1575. 10.1093/nar/24.8.1574 8628694 PMC145803

[men13252-bib-0122] Suh, A. , Smeds, L. , & Ellegren, H. (2018). Abundant recent activity of retrovirus‐like retrotransposons within and among flycatcher species implies a rich source of structural variation in songbird genomes. Molecular Ecology, 27(1), 99–111. 10.1111/mec.14439 29171119

[men13252-bib-0123] Sun, H. , Rowan, B. A. , Flood, P. J. , Brandt, R. , Fuss, J. , Hancock, A. M. , Michelmore, R. W. , Huettel, B. , & Schneeberger, K. (2019). Linked‐read sequencing of gametes allows efficient genome‐wide analysis of meiotic recombination. Nature Communications, 10(1), 4310 10.1038/s41467-019-12209-2 PMC6754367 31541084

[men13252-bib-0124] Tanaka, Y. , Asano, T. , Kanemitsu, Y. , Goto, T. , Yoshida, Y. , Yasuba, K. , & Kobata, K. (2019). Positional differences of intronic transposons in pAMT affect the pungency level in chili pepper through altered splicing efficiency. The Plant Journal, 100(4), 693–705. 10.1111/tpj.14462 31323150

[men13252-bib-0125] Teeling, E. C. , Vernes, S. C. , Dávalos, L. M. , Ray, D. A. , Gilbert, M. T. P. , & Myers, E. , & Bat1K Consortium (2018). Bat biology, genomes, and the Bat1K project: To generate chromosome‐level genomes for all living bat species. Annual Review of Animal Biosciences, 6(1), 23–46. 10.1146/annurev-animal-022516-022811 29166127

[men13252-bib-0126] Thomma, B. P. H. J. , Seidl, M. F. , Shi‐Kunne, X. , Cook, D. E. , Bolton, M. D. , van Kan, J. A. L. , & Faino, L. (2016). Mind the gap; seven reasons to close fragmented genome assemblies. Fungal Genetics and Biology, 90, 24–30. 10.1016/j.fgb.2015.08.010 26342853

[men13252-bib-0127] Tilak, M.‐K. , Botero‐Castro, F. , Galtier, N. , & Nabholz, B. (2018). Illumina library preparation for sequencing the GC‐Rich fraction of heterogeneous genomic DNA. Genome Biology and Evolution, 10(2), 616–622. 10.1093/gbe/evy022 29385572 PMC5808798

[men13252-bib-0128] Walker, B. J. , Abeel, T. , Shea, T. , Priest, M. , Abouelliel, A. , Sakthikumar, S. , Cuomo, C. A. , Zeng, Q. , Wortman, J. , Young, S. K. , & Earl, A. M. (2014). pilon: An integrated tool for comprehensive microbial variant detection and genome assembly improvement. PLoS One, 9(11), e112963 10.1371/journal.pone.0112963 25409509 PMC4237348

[men13252-bib-0129] Wallberg, A. , Bunikis, I. , Pettersson, O. V. , Mosbech, M.‐B. , Childers, A. K. , Evans, J. D. , Mikheyev, A. S. , Robertson, H. M. , Robinson, G. E. , & Webster, M. T. (2019). A hybrid de novo genome assembly of the honeybee, Apis mellifera, with chromosome‐length scaffolds. BMC Genomics, 20(1), 275 10.1186/s12864-019-5642-0 30961563 PMC6454739

[men13252-bib-0130] Warren, W. C. , Clayton, D. F. , Ellegren, H. , Arnold, A. P. , Hillier, L. D. W. , Künstner, A. , Searle, S. , White, S. , Vilella, A. J. , Fairley, S. , Heger, A. , Kong, L. , Ponting, C. P. , Jarvis, E. D. , Mello, C. V. , Minx, P. , Lovell, P. , Velho, T. A. F. , Ferris, M. , … Wilson, R. K. (2010). The genome of a songbird. Nature, 464(7289), 757–762. 10.1038/nature08819 20360741 PMC3187626

[men13252-bib-0131] Warren, W. C. , Hillier, L. D. W. , Tomlinson, C. , Minx, P. , Kremitzki, M. , Graves, T. , Markovic, C. , Bouk, N. , Pruitt, K. D. , Thibaud‐Nissen, F. , Schneider, V. , Mansour, T. A. , Brown, C. T. , Zimin, A. , Hawken, R. , Abrahamsen, M. , Pyrkosz, A. B. , Morisson, M. , Fillon, V. , … Cheng, H. H. (2017). A new chicken genome assembly provides insight into avian genome structure. G3: Genes, Genomes, Genetics, 7(1), 109–117. 10.1534/g3.116.035923 27852011 PMC5217101

[men13252-bib-0132] Waterhouse, R. M. , Seppey, M. , Simão, F. A. , Manni, M. , Ioannidis, P. , Klioutchnikov, G. , Kriventseva, E. V. , & Zdobnov, E. M. (2017). busco applications from quality assessments to gene prediction and phylogenomics. Molecular Biology and Evolution, 35(3), 543–548. 10.1093/molbev/msx319 PMC5850278 29220515

[men13252-bib-0133] Watson, M. , & Warr, A. (2019). Errors in long‐read assemblies can critically affect protein prediction. Nature Biotechnology, 37(2), 124–126. 10.1038/s41587-018-0004-z 30670796

[men13252-bib-0134] Weisenfeld, N. I. , Kumar, V. , Shah, P. , Church, D. M. , & Jaffe, D. B. (2017). Direct determination of diploid genome sequences. Genome Research, 27(5), 757–767. 10.1101/gr.214874.116 28381613 PMC5411770

[men13252-bib-0135] Weissensteiner, M. H. , Bunikis, I. , Catalan, A. , Francoijs, K.‐J. , Knief, U. , Heim, W. , Peona, V. , Pophaly, S. D. , Sedlazeck, F. J. , Suh, F. J. , Warmuth, F. J. , & Wolf, J. B. (2019). The population genomics of structural variation in a songbird genus. bioRxiv, 830356 10.1101/830356 PMC7341801 32636372

[men13252-bib-0136] Weissensteiner, M. H. , Pang, A. W. C. , Bunikis, I. , Hoijer, I. , Vinnere‐Petterson, O. , Suh, A. , & Wolf, J. B. W. (2017). Combination of short‐read, long‐read, and optical mapping assemblies reveals large‐scale tandem repeat arrays with population genetic implications. Genome Research, 27(5), 697–708. 10.1101/gr.215095.116 28360231 PMC5411765

[men13252-bib-0137] Weissensteiner, M. H. , & Suh, A. (2019). Repetitive DNA: The dark matter of avian genomics In Kraus R. H. S. (Ed.), Avian genomics in ecology and evolution: From the lab into the wild (pp. 93–150). Springer International Publishing.

[men13252-bib-0138] Wenger, A. M. , Peluso, P. , Rowell, W. J. , Chang, P.‐C. , Hall, R. J. , Concepcion, G. T. , Ebler, J. , Fungtammasan, A. , Kolesnikov, A. , Olson, N. D. , Töpfer, A. , Alonge, M. , Mahmoud, M. , Qian, Y. , Chin, C.‐S. , Phillippy, A. M. , Schatz, M. C. , Myers, G. , DePristo, M. A. , … Hunkapiller, M. W. (2019). Accurate circular consensus long‐read sequencing improves variant detection and assembly of a human genome. Nature Biotechnology, 37(10), 1155–1162. 10.1038/s41587-019-0217-9 PMC6776680 31406327

[men13252-bib-0139] Westbrook, C. J. , Karl, J. A. , Wiseman, R. W. , Mate, S. , Koroleva, G. , Garcia, K. , Sanchez‐Lockhart, M. , O’Connor, D. H. , & Palacios, G. (2015). No assembly required: Full‐length MHC class I allele discovery by PacBio circular consensus sequencing. Human Immunology, 76(12), 891–896. 10.1016/j.humimm.2015.03.022 26028281

[men13252-bib-0140] Wicker, T. , Sabot, F. , Hua‐Van, A. , Bennetzen, J. L. , Capy, P. , Chalhoub, B. , Flavell, A. , Leroy, P. , Morgante, M. , Panaud, O. , Paux, E. , SanMiguel, P. , & Schulman, A. H. (2007). A unified classification system for eukaryotic transposable elements. Nature Reviews Genetics, 8(12), 973–982. 10.1038/nrg2165 17984973

[men13252-bib-0141] Willard, H. F. , & Waye, J. S. (1987). Hierarchical order in chromosome‐specific human alpha satellite DNA. Trends in Genetics, 3, 192–198. 10.1016/0168-9525(87)90232-0

[men13252-bib-0142] Wyman, S. K. , Jansen, R. K. , & Boore, J. L. (2004). Automatic annotation of organellar genomes with DOGMA. Bioinformatics, 20(17), 3252–3255. 10.1093/bioinformatics/bth352 15180927

[men13252-bib-0143] Xu, L. , Auer, G. , Peona, V. , Suh, A. , Deng, Y. , Feng, S. , Zhang, G. , Blom, M. P. K. , Christidis, L. , Prost, S. , Irestedt, M. , & Zhou, Q. I. (2019). Dynamic evolutionary history and gene content of sex chromosomes across diverse songbirds. Nature Ecology and Evolution, 3(5), 834–844. 10.1038/s41559-019-0850-1 30936435

[men13252-bib-0144] Yazdi, H. P. , & Ellegren, H. (2018). A Genetic map of Ostrich Z chromosome and the role of inversions in avian sex chromosome evolution. Genome Biology and Evolution, 10(8), 2049–2060. 10.1093/gbe/evy163 30099482 PMC6105114

[men13252-bib-0145] Yeo, S. , Coombe, L. , Warren, R. L. , Chu, J. , & Birol, I. (2017). arcs: Scaffolding genome drafts with linked reads. Bioinformatics, 34(5), 725–731. 10.1093/bioinformatics/btx675 PMC6030987 29069293

[men13252-bib-0146] Yoshimura, J. , Ichikawa, K. , Shoura, M. J. , Artiles, K. L. , Gabdank, I. , Wahba, L. , Smith, C. L. , Edgley, M. L. , Rougvie, A. E. , Fire, A. Z. , Morishita, S. , & Schwarz, E. M. (2019). Recompleting the *Caenorhabditis elegans* genome. Genome Research, 29(6), 1009–1022. 10.1101/gr.244830.118 31123080 PMC6581061

[men13252-bib-0147] Zhang, G. , Li, C. , Li, Q. , Li, B. , Larkin, D. M. , Lee, C. , Storz, J. F. , Antunes, A. , Greenwold, M. J. , Meredith, R. W. , Odeen, A. , Cui, J. , Zhou, Q. , Xu, L. , Pan, H. , Wang, Z. , Jin, L. , Zhang, P. , Hu, H. , … Froman, D. P. (2014). Comparative genomics reveals insights into avian genome evolution and adaptation. Science, 346(6215), 1311–1320. 10.1126/science.1251385 25504712 PMC4390078

[men13252-bib-0148] Zhang, Y. , Cheng, T. C. , Huang, G. , Lu, Q. , Surleac, M. D. , Mandell, J. D. , Pontarotti, P. , Petrescu, A. J. , Xu, A. , Xiong, Y. , & Schatz, D. G. (2019). Transposon molecular domestication and the evolution of the RAG recombinase. Nature, 569(7754), 79–84. 10.1038/s41586-019-1093-7 30971819 PMC6494689

[men13252-bib-0149] Zheng, G. X. Y. , Lau, B. T. , Schnall‐Levin, M. , Jarosz, M. , Bell, J. M. , Hindson, C. M. , Kyriazopoulou‐Panagiotopoulou, S. , Masquelier, D. A. , Merrill, L. , Terry, J. M. , Mudivarti, P. A. , Wyatt, P. W. , Bharadwaj, R. , Makarewicz, A. J. , Li, Y. , Belgrader, P. , Price, A. D. , Lowe, A. J. , Marks, P. , … Ji, H. P. (2016). Haplotyping germline and cancer genomes with high‐throughput linked‐read sequencing. Nature Biotechnology, 34, 303 10.1038/nbt.3432 PMC4786454 26829319

[men13252-bib-0150] Zhou, Q. , Zhang, J. , Bachtrog, D. , An, N. , Huang, Q. , Jarvis, E. D. , Gilbert, M. T. P. , & Zhang, G. (2014). Complex evolutionary trajectories of sex chromosomes across bird taxa. Science, 346(6215), 1246338 10.1126/science.1246338 25504727 PMC6445272

