# Supplementary material for: Identifying the causes and consequences of assembly gaps using a multiplatform genome assembly of a bird‐of‐paradise
Source: Mol Ecol Resour. 2020 Oct 10;21(1):263–86. doi: 10.1111/1755-0998.13252 (PMC7757076; doi:10.1111/1755-0998.13252)
Supplement: Supplementary file 1 — Figures S1‐S14 [file MEN-21-263-s001.pdf]

Identifying the causes and consequences of assembly gaps using a multiplatform genome assembly of a bird-of-paradise

Valentina Peona[1,2], Mozes P.K. Blom[3,4], Luohao Xu[5], Reto Burri[6], Shawn Sullivan[7], Ignas Bunikis[8], Ivan Liachko[7], Tri Haryoko[9], Knud A. Jønsson[10], Qi Zhou[5,11,12], Martin Irestedt[3], Alexander Suh[1,2,13]

Affiliation

[1] Department of Ecology and Genetics – Evolutionary Biology, Uppsala University, Science for Life Laboratories, Norbyvägen 18D, SE-752 36, Uppsala, Sweden
[2] Department of Organismal Biology – Systematic Biology, Uppsala University, Norbyvägen 18D, SE-752 36, Uppsala, Sweden
[3] Department of Bioinformatics and Genetics, Swedish Museum of Natural History, SE-104 05, Stockholm, Sweden
[4] Museum für Naturkunde, Leibniz Institut für Evolutions- und Biodiversitätsforschung, Berlin, Germany
[5] Department of Neurosciences and Developmental Biology, University of Vienna, Vienna, Austria
[6] Department of Population Ecology, Institute of Ecology and Evolution, Friedrich-Schiller-University Jena, Dornburger Strasse 159, D-07743 Jena, Germany
[7] Phase Genomics, Inc. 1617 8th Ave N, Seattle, WA 98109 USA
[8] Uppsala Genome Center, Science for Life Laboratory, Dept. of Immunology, Genetics and Pathology, Uppsala University, SE‐752 37, Uppsala, Sweden
[9] Museum Zoologicum Bogoriense, Research Centre for Biology, Indonesian Institute of Sciences (LIPI), Cibinong, Indonesia
[10] Natural History Museum of Denmark, University of Copenhagen, Universitetsparken 15, DK-2100 Copenhagen, Denmark
[11] MOE Laboratory of Biosystems Homeostasis & Protection, Life Sciences Institute, Zhejiang University, Hangzhou, China
[12] Center for Reproductive Medicine, The 2nd Affiliated Hospital, School of Medicine, Zhejiang University
[13] School of Biological Sciences – Organisms and the Environment, University of East Anglia, NR4 7TJ, Norwich, UK

Supplementary Figures

Figures S4, S8 and S13 are shown in separate documents due to their large sizes.

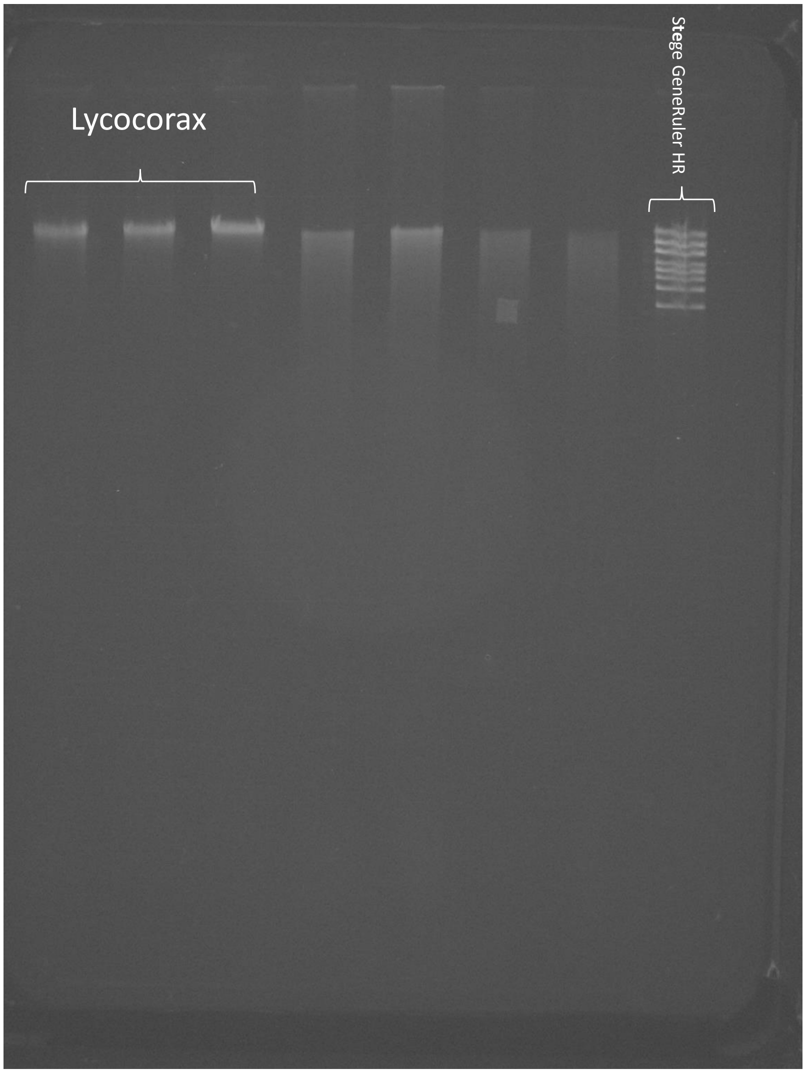

Figure S1. Agarose gel electrophoretic run of the reference female DNA library used for generating sequence data for lycPyrIL, lycPyrSN1, and lycPyrPB. DNA was extracted using magnetic beads with the Kingfisher Duo robot and the top band of the GeneRuler High Range DNA Ladder corresponds to 48,502 bp fragment length. A 0.6% agarose gel was run with 1X TAE buffer.

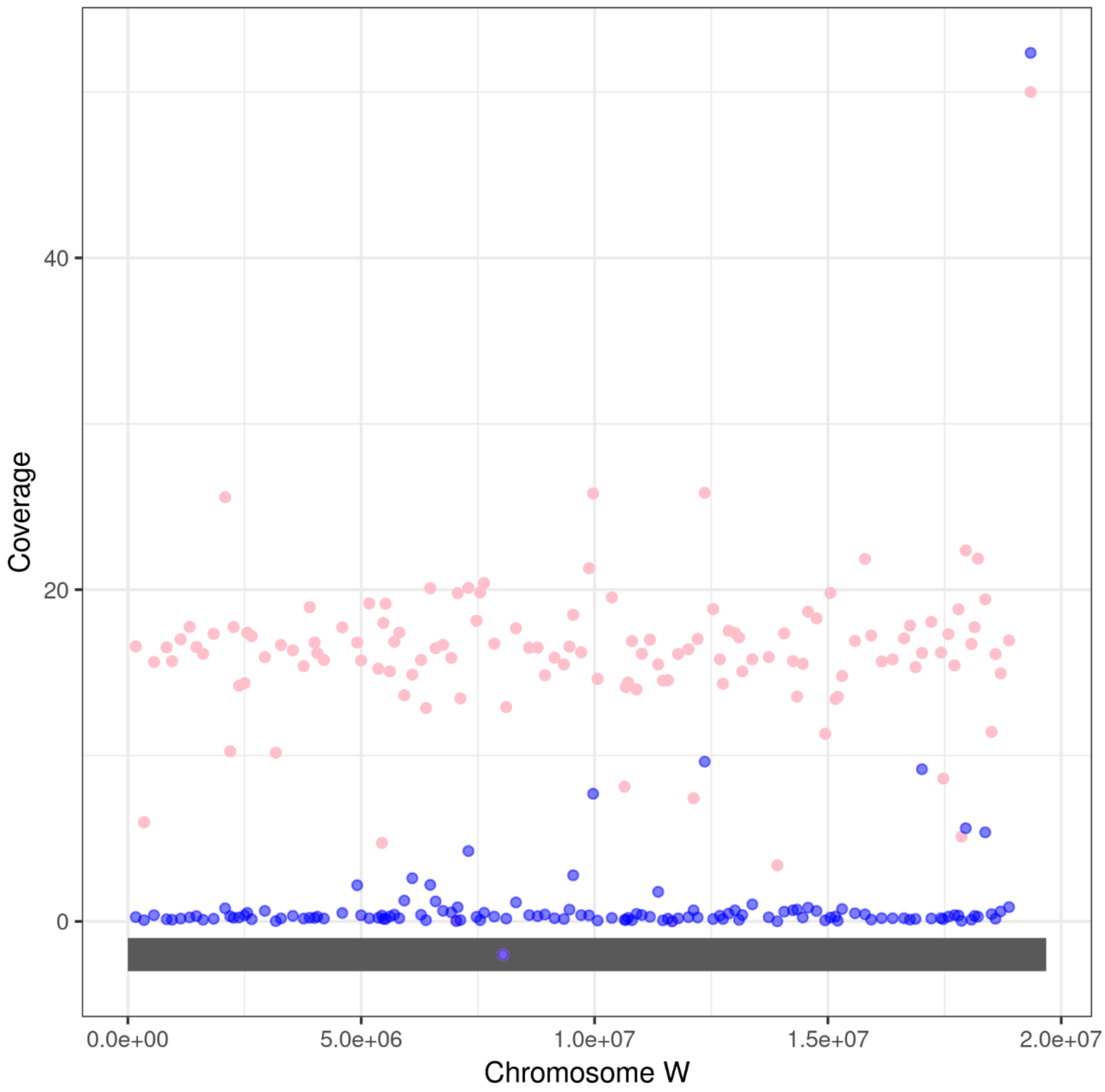

Figure S2a. Mean coverage across the W chromosome of lycPyr6 (final assembly version) using female and male 10X Genomics linked reads. Each dot corresponds to a contig and is placed at the contig midpoint. Pink: female coverage (lycPyrSN1 library); blue: male coverage (data from Peona et al. 2020). Contigs with coverage >100 were placed at the bottom within the grey bar. Most of the contigs show the expected coverage in female and males with the exception of a few contigs were coverage is very low or very high in both sexes, likely because of their repetitive content and low mappability.

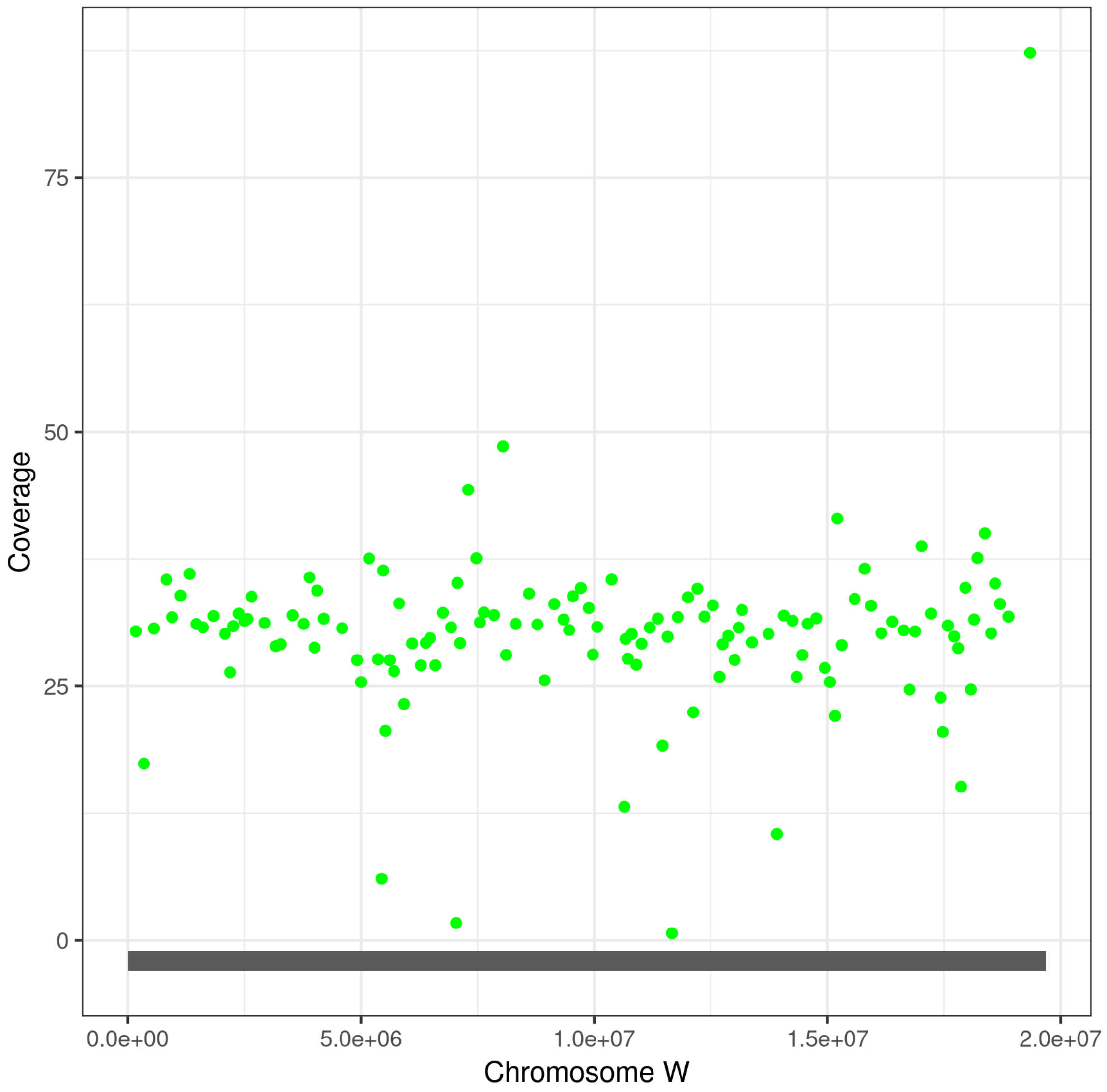

Figure S2b. Mean coverage across the W chromosome of lycPyr6 (final assembly version) using female PacBio reads. Each dot corresponds to a contig and is placed at the contig midpoint.

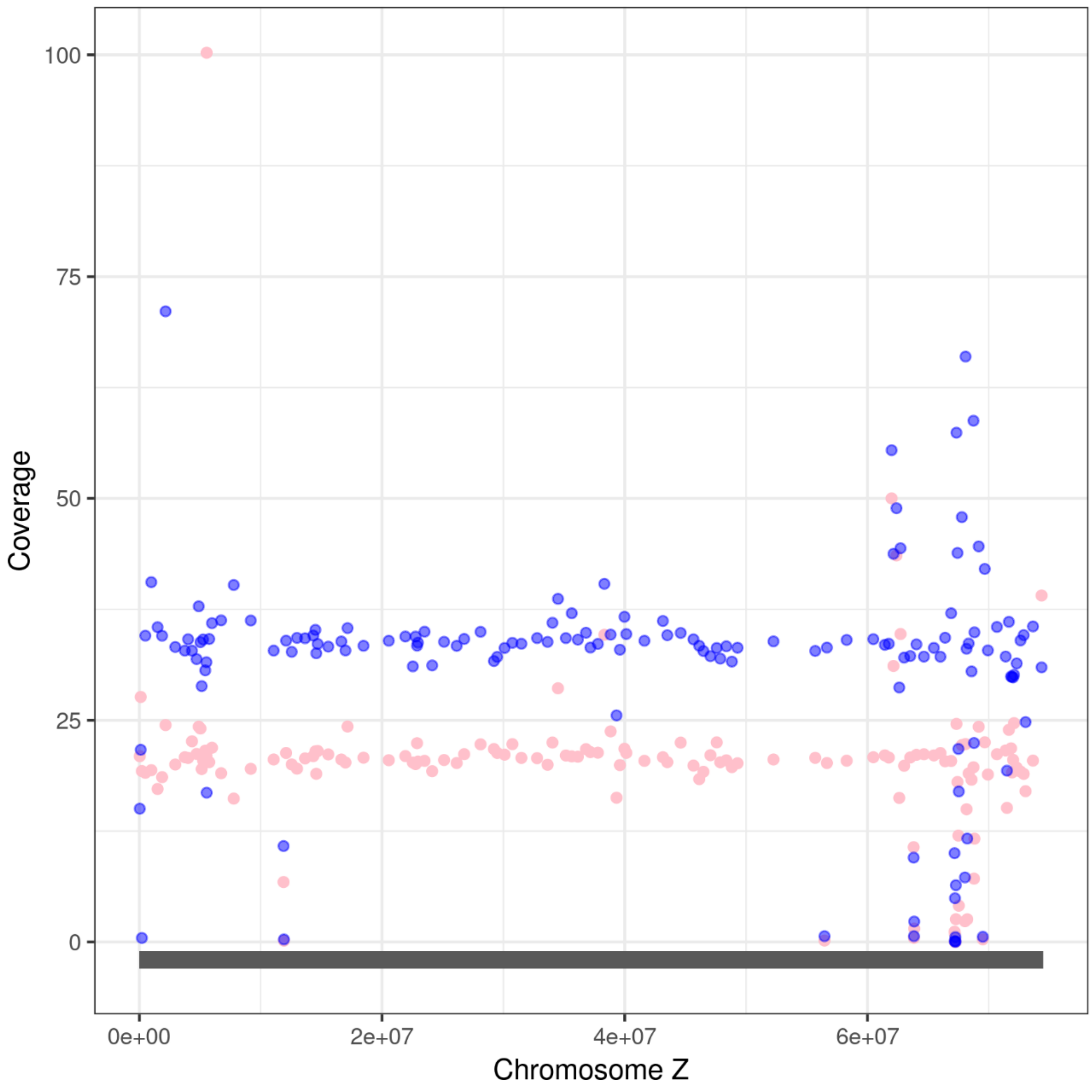

Figure S3a. Mean coverage across the Z chromosome of lycPyr6 (final assembly version) using 10X Genomics reads (lycPyrSN1 library). Each dot corresponds to a contig and is placed at the contig midpoint. Pink: female coverage (lycPyrSN1 library); blue: male coverage (data from Peona et al. 2020). Contigs with coverage >100 were placed at the bottom within the grey bar. Most of the contigs show the expected coverage in female and males with the exception of a few contigs were coverage is very low or very high in both sexes, likely because of their repetitive content and low mappability.

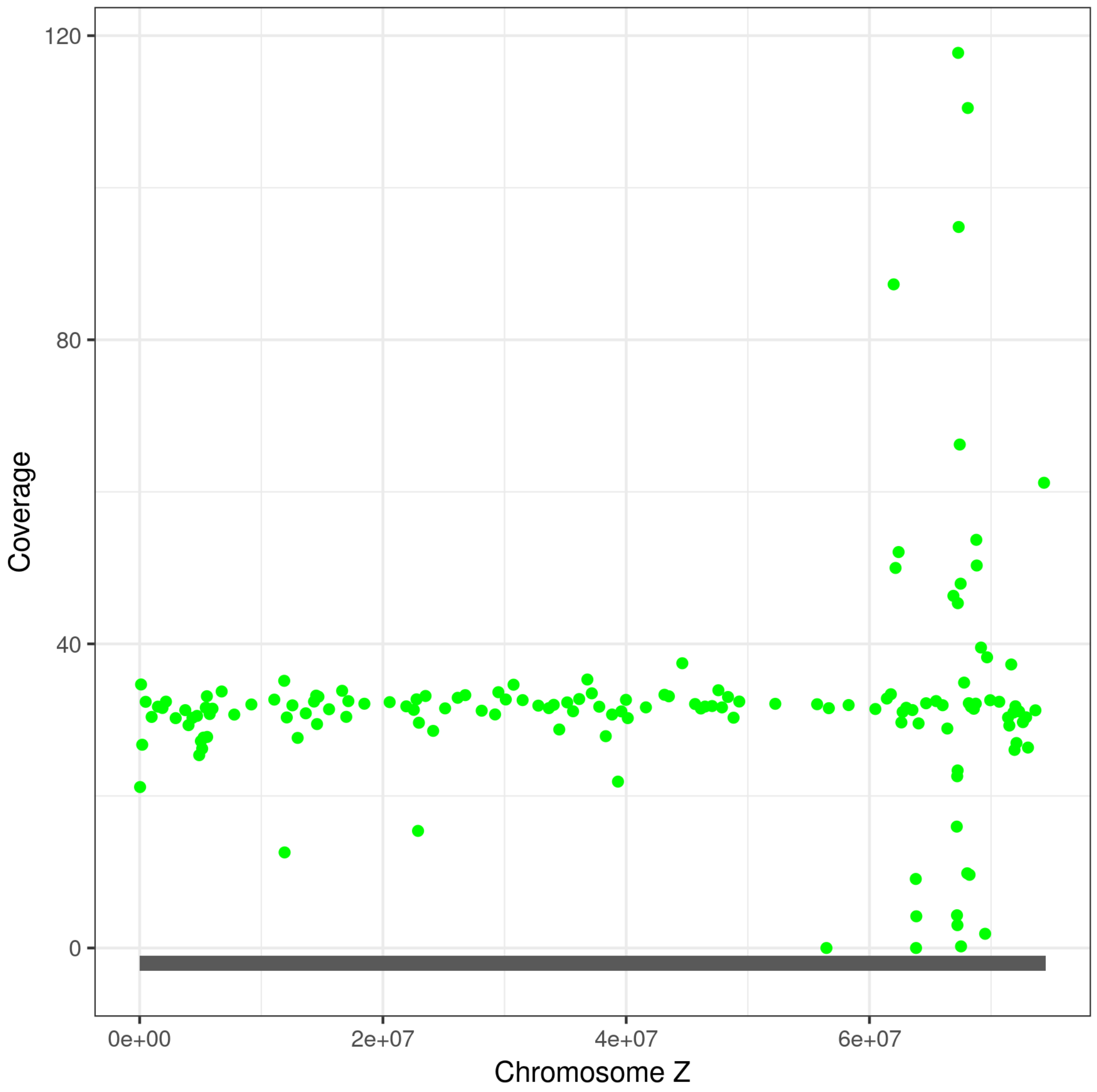

Figure S3b. Mean coverage across the Z chromosome of lycPyr6 (final assembly version) using female PacBio reads. Each dot corresponds to a contig and is placed at the contig midpoint.

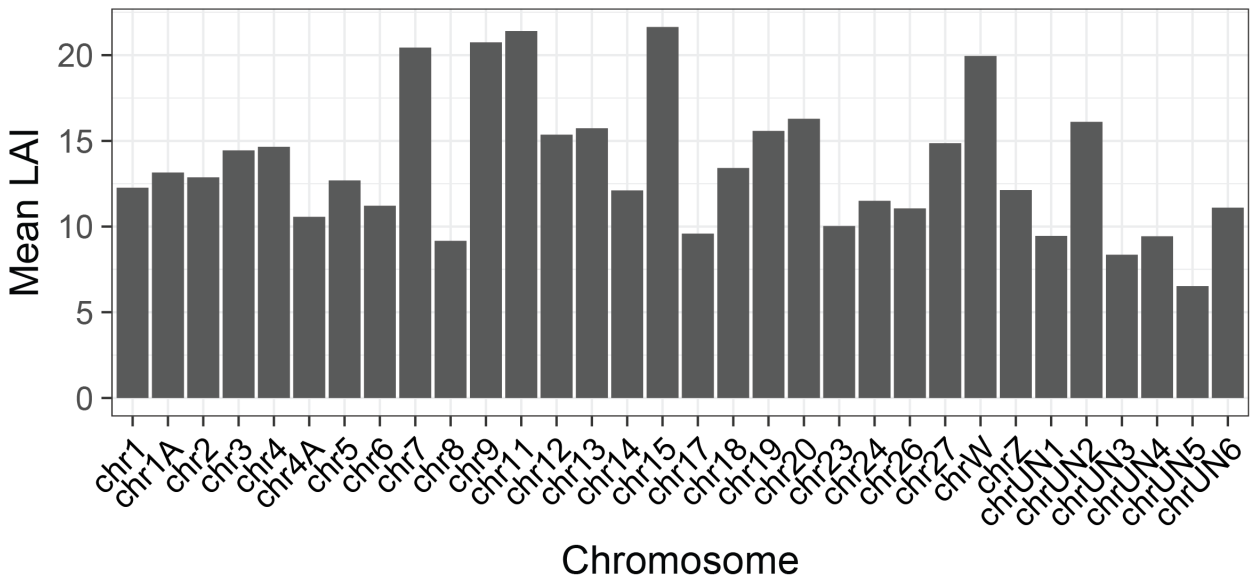

Figure S5. Barplot of the mean LTR Assembly Index (Jiang et al. 2018) calculated on lycPyr6 (final assembly version) per chromosome. Draft quality is characterized by LAI score less than 10; reference quality by LAI score ranges from 10 to 20; and gold quality by LAI score greater than 20.

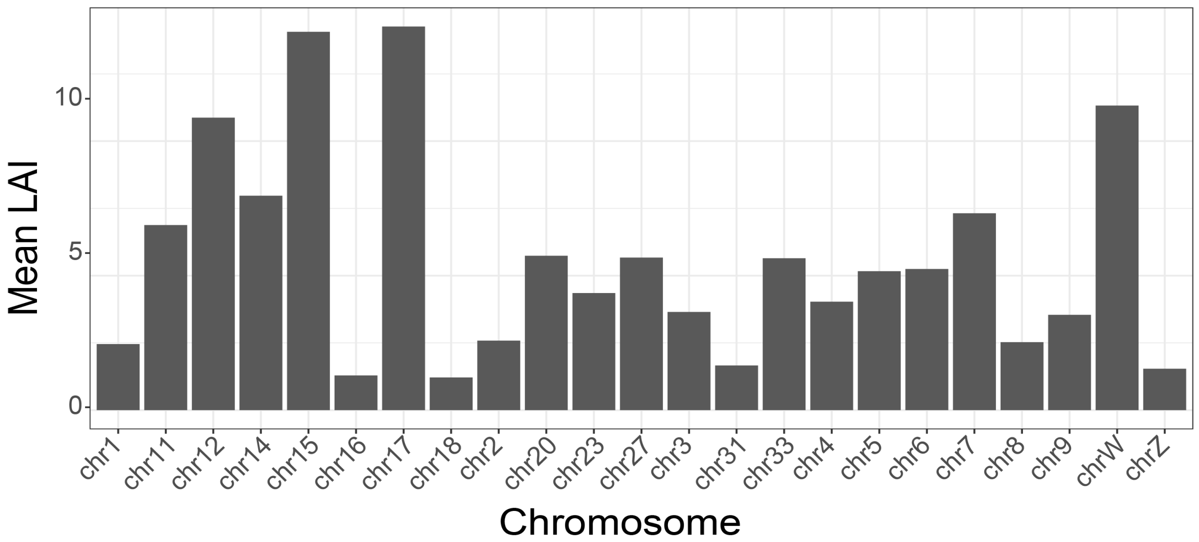

Figure S6. Barplot of the mean LTR Assembly Index (Jiang et al. 2018) calculated on the chicken assembly galGal6a per chromosome. Draft quality is characterized by LAI score less than 10; reference quality by LAI score ranges from 10 to 20; and gold quality by LAI score greater than 20.

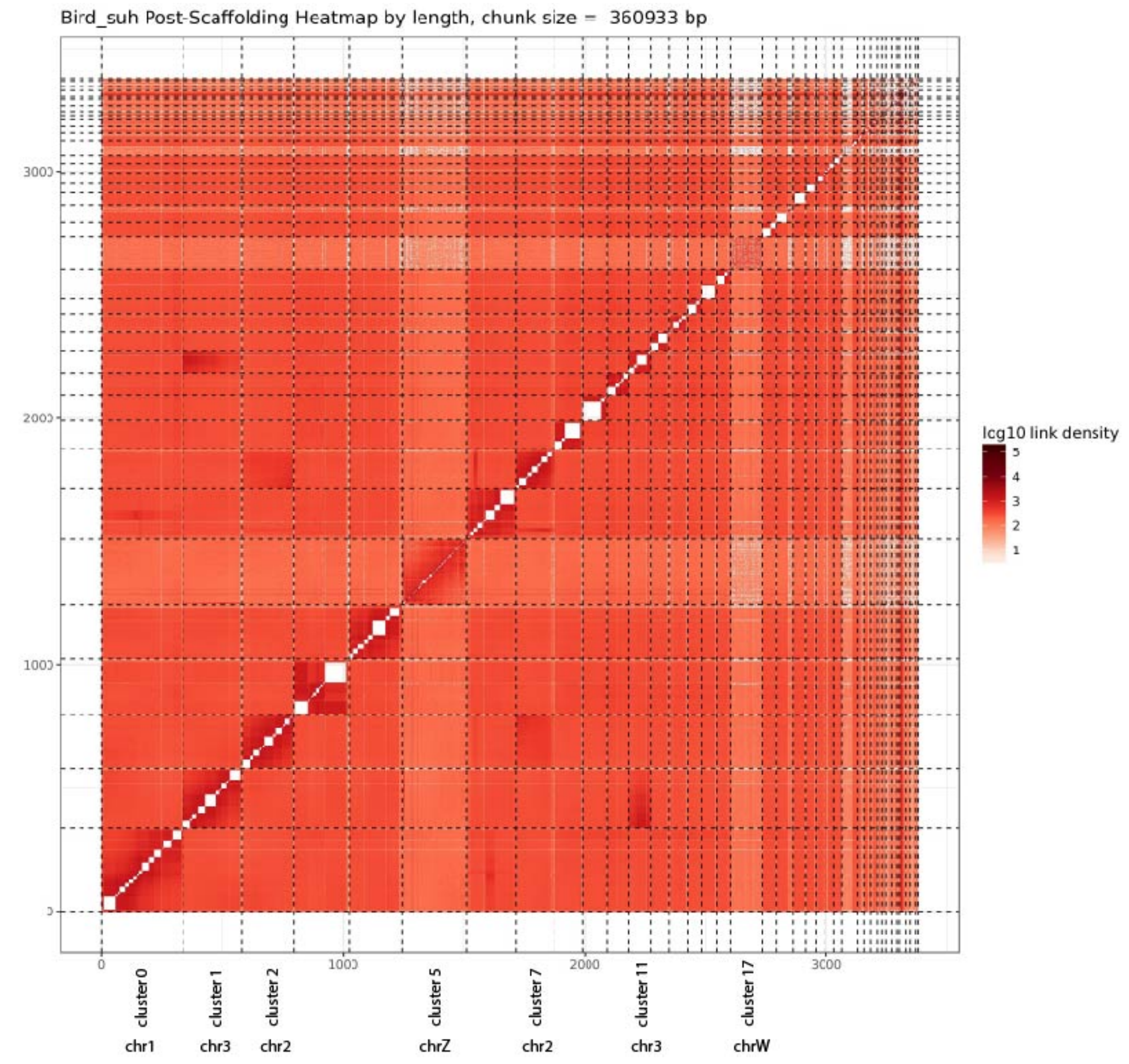

Figure S7. Post-clustering Hi-C heatmap of lycPyr4 produced by Proximo (Phase Genomics). Each dark red square represents a cluster (chromosome model) of contigs/scaffolds. The white little squares represent the contigs/scaffolds within each chromosome model; the white colour indicates that the interactions within single contigs/scaffolds are not shown. Clusters 5 and 17 have remarkably fewer inter-cluster interactions because they are the sex chromosomes (Z and W, respectively) and have half of the coverage with respect to the autosomes. The darker red shades outside the main chromosome models represent inter-cluster interactions, in this case the likely interaction between chromosome arms that remained separated during the scaffolding process. More specifically, clusters 2 and 7 are the two arms of chromosome 2; clusters 1 and 11 are the two arms of chromosome 3.

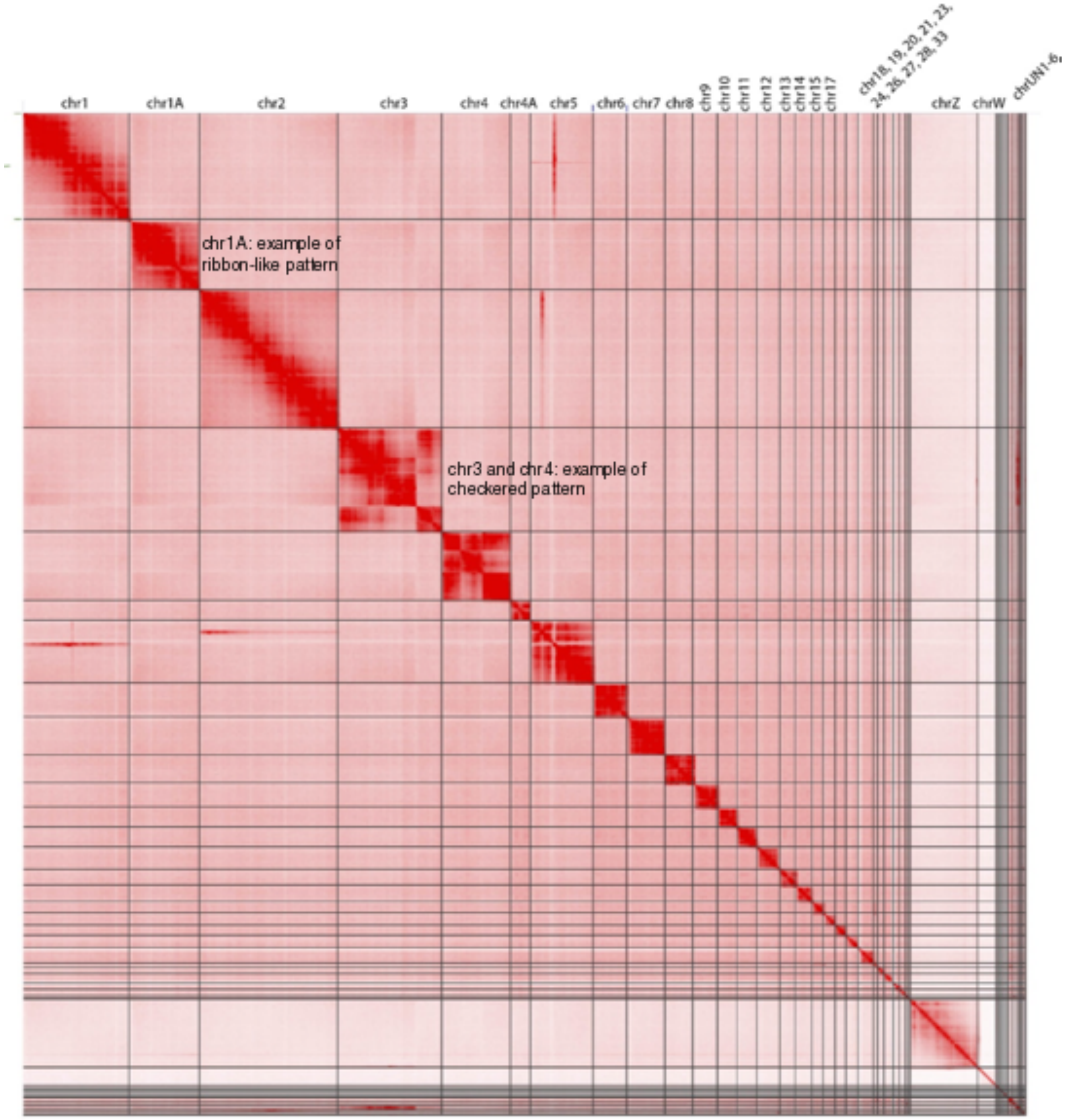

Figure S9. Post-clustering Hi-C heatmap of lycPyr5 produced by Juicer. The red streaks outside the assembly diagonal of the chromosome models represent inter-cluster interactions, i.e., two misassemblies at the primary assembly level. These two contigs were cut in lycPyr6 according to the Hi-C heatmap, and the halves with strong interchromosomal interactions were placed onto the other two chromosomes (from chromosome 5 to chromosome 1 and chromosome 2).

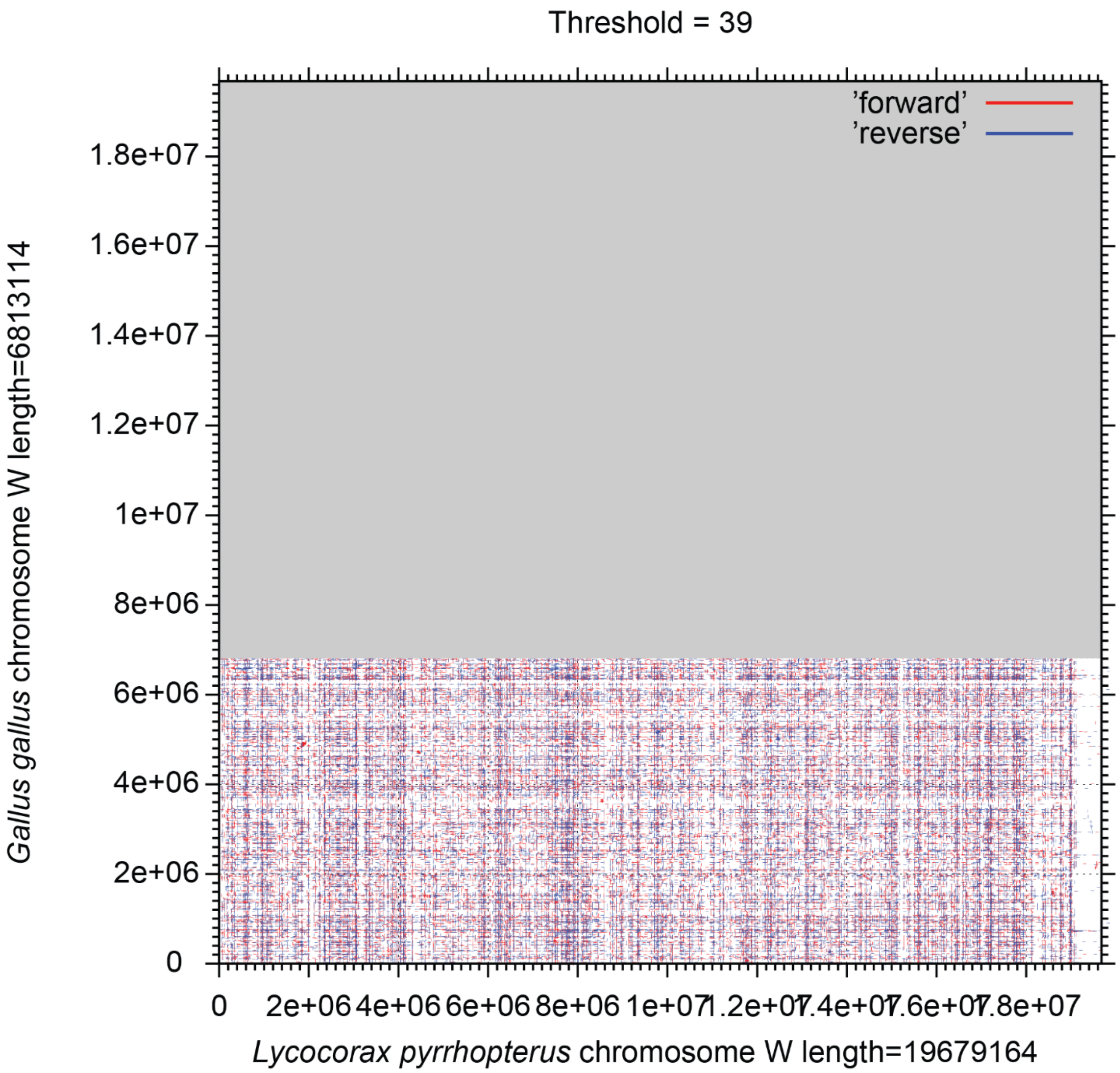

Figure S10. Dotplot of the alignment between the W chromosomes of *Gallus gallus* (chicken) galGal5 and *Lycocorax pyrrhopterus* (paradise crow) lycPyr6.

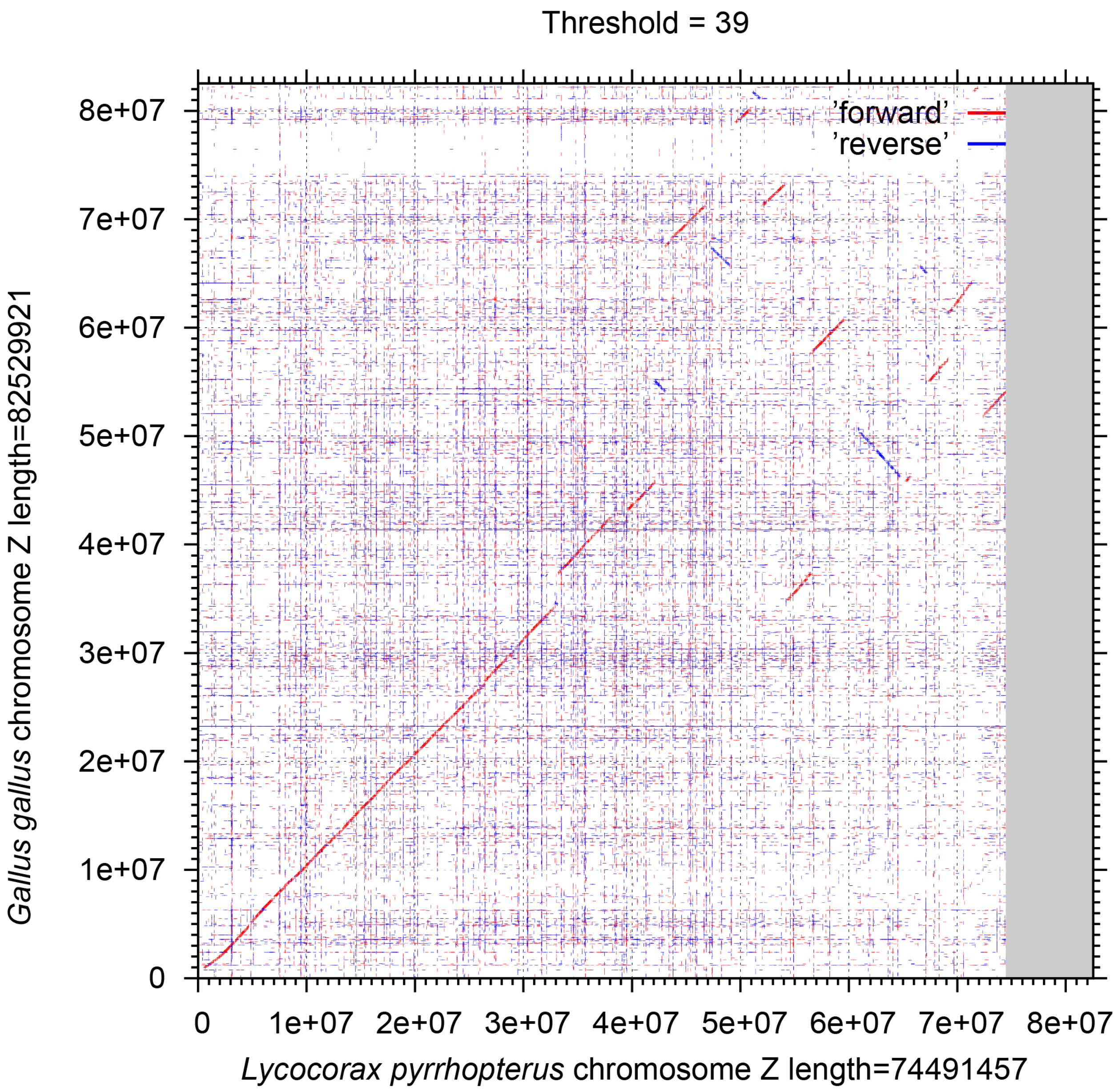

Figure S11. Dotplot of the alignment between the Z chromosomes of *Gallus gallus* (chicken) galGal5 and *Lycocorax pyrrhopterus* (paradise crow) lycPyr6.

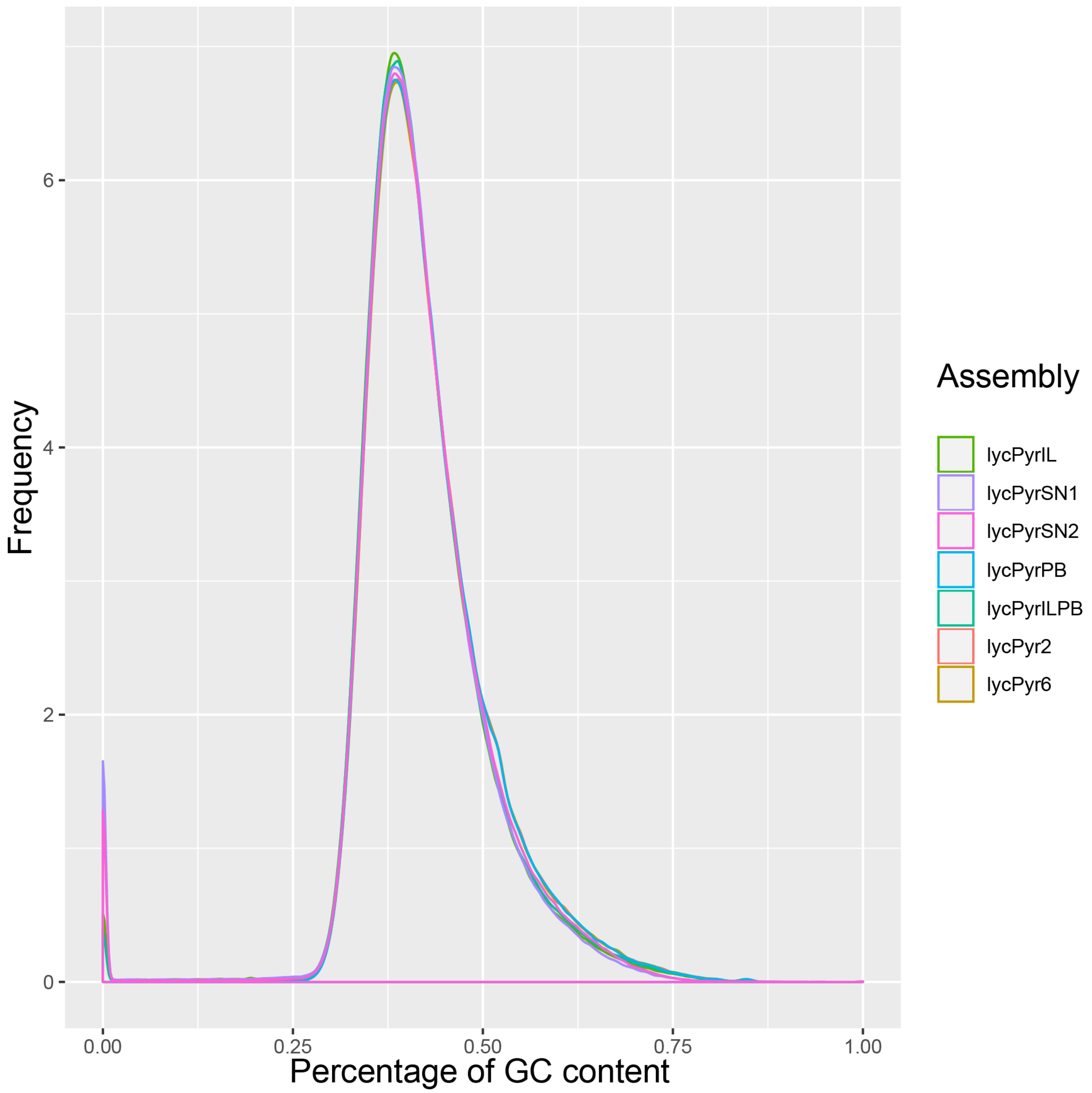

Figure S12. Percentage of GC content calculated per window of 1 kb for each lycPyr assembly version.

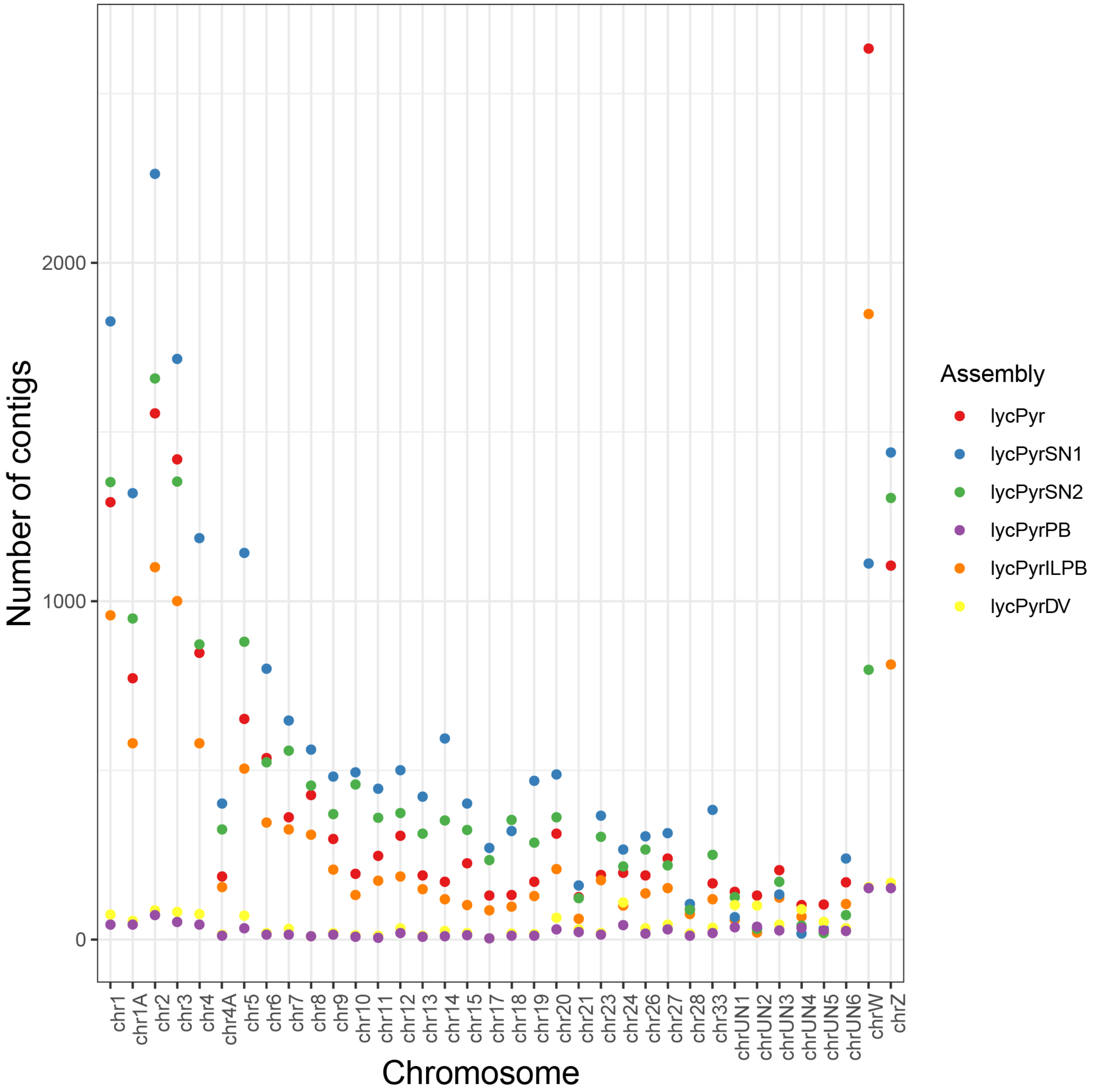

Figure S14. Number of contigs belonging to each chromosome model for each lycPyr assembly version.
