# Supplementary material for: Identifying the causes and consequences of assembly gaps using a multiplatform genome assembly of a bird‐of‐paradise
Source: Mol Ecol Resour. 2020 Oct 10;21(1):263–86. doi: 10.1111/1755-0998.13252 (PMC7757076; doi:10.1111/1755-0998.13252)
Supplement: Supplementary file 2 — Figure S4 [file MEN-21-263-s002.pdf]

Identifying the causes and consequences of assembly gaps using a multiplatform genome assembly of a bird-of-paradise

Valentina Peona[1,2], Mozes P.K. Blom[3,4], Luohao Xu[5], Reto Burri[6], Shawn Sullivan[7], Ignas Bunikis[8], Ivan Liachko[7], Tri Haryoko[9], Knud A. Jønsson[10], Qi Zhou[5,11,12], Martin Irestedt[3], Alexander Suh[1,2,13]

Affiliation

[1] Department of Ecology and Genetics – Evolutionary Biology, Uppsala University, Science for Life Laboratories, Norbyvägen 18D, SE-752 36, Uppsala, Sweden
[2] Department of Organismal Biology – Systematic Biology, Uppsala University, Norbyvägen 18D, SE-752 36, Uppsala, Sweden
[3] Department of Bioinformatics and Genetics, Swedish Museum of Natural History, SE-104 05, Stockholm, Sweden
[4] Museum für Naturkunde, Leibniz Institut für Evolutions- und Biodiversitätsforschung, Berlin, Germany
[5] Department of Neurosciences and Developmental Biology, University of Vienna, Vienna, Austria
[6] Department of Population Ecology, Institute of Ecology and Evolution, Friedrich-Schiller-University Jena, Dornburger Strasse 159, D-07743 Jena, Germany
[7] Phase Genomics, Inc. 1617 8th Ave N, Seattle, WA 98109 USA
[8] Uppsala Genome Center, Science for Life Laboratory, Dept. of Immunology, Genetics and Pathology, Uppsala University, SE-752 37, Uppsala, Sweden
[9] Museum Zoologicum Bogoriense, Research Centre for Biology, Indonesian Institute of Sciences (LIPI), Cibinong, Indonesia
[10] Natural History Museum of Denmark, University of Copenhagen, Universitetsparken 15, DK-2100 Copenhagen, Denmark
[11] MOE Laboratory of Biosystems Homeostasis & Protection, Life Sciences Institute, Zhejiang University, Hangzhou, China
[12] Center for Reproductive Medicine, The 2nd Affiliated Hospital, School of Medicine, Zhejiang University
[13] School of Biological Sciences – Organisms and the Environment, University of East Anglia, NR4 7TJ, Norwich, UK

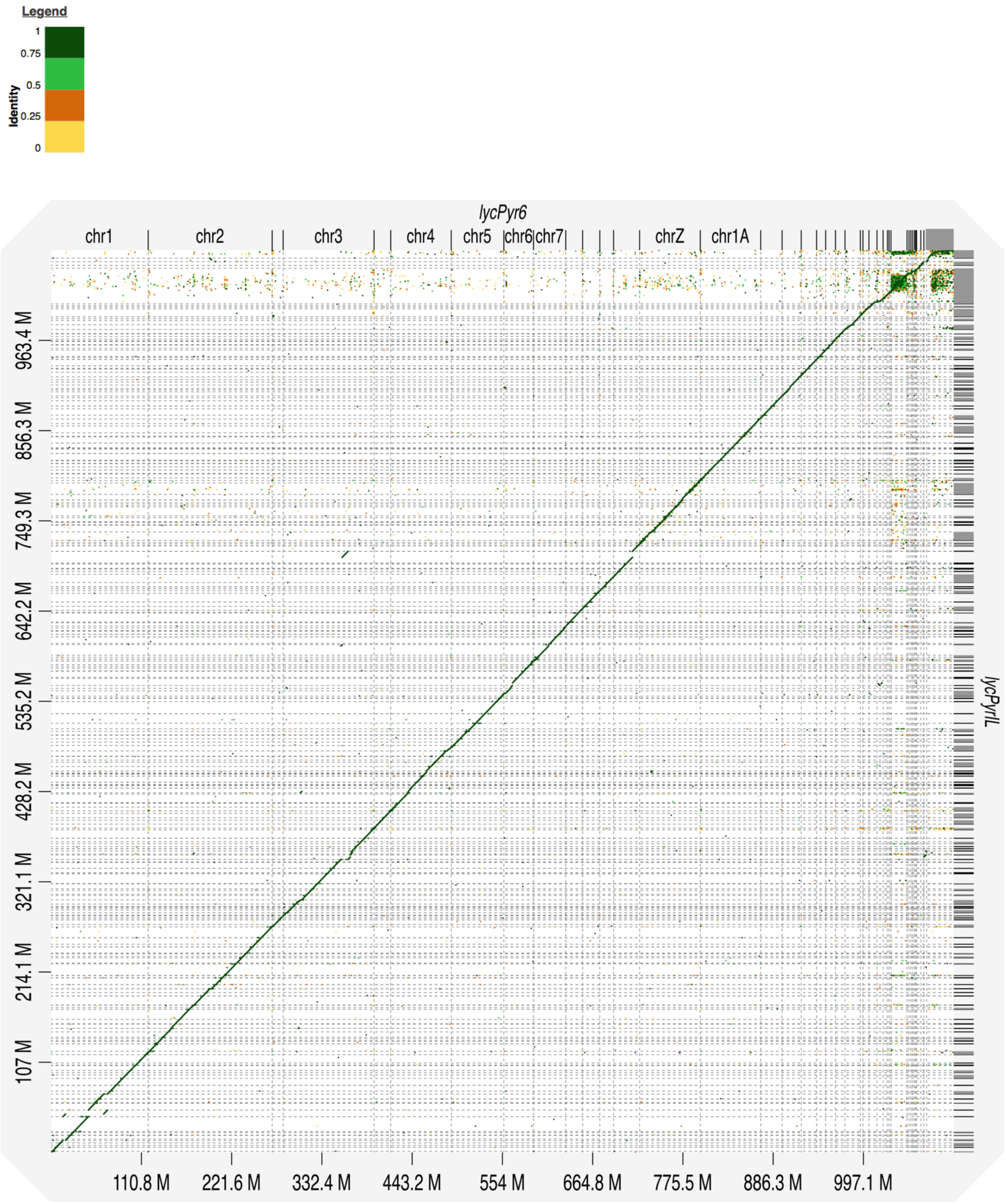

Figure S4. Dotplots of the alignments of the different lycPyr assembly versions against lycPyr6 (final assembly version). All-by-all alignments and plots were generated using D-Genies (http://dgenies.toulouse.inra.fr/).

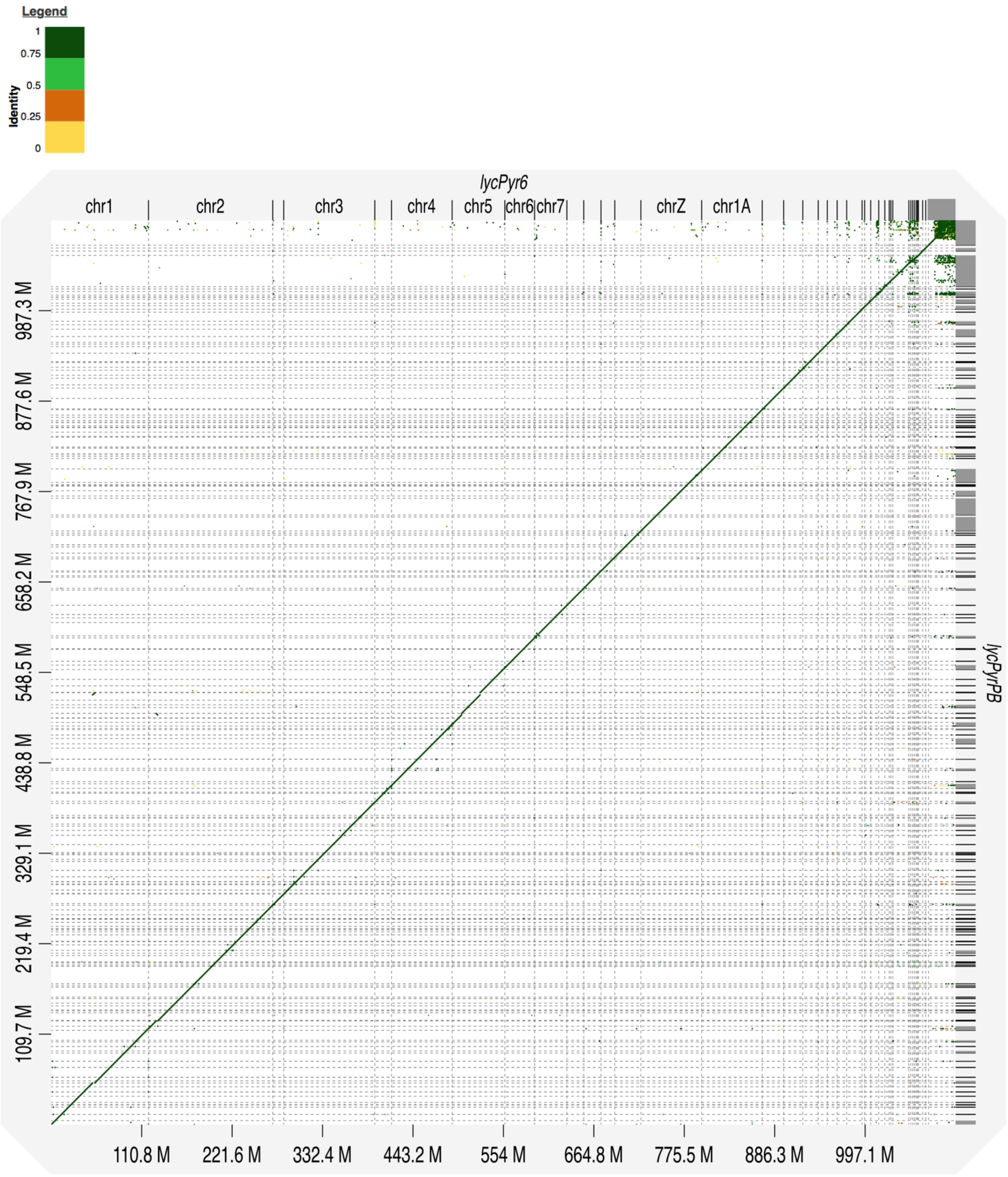

Figure S4. Continued.

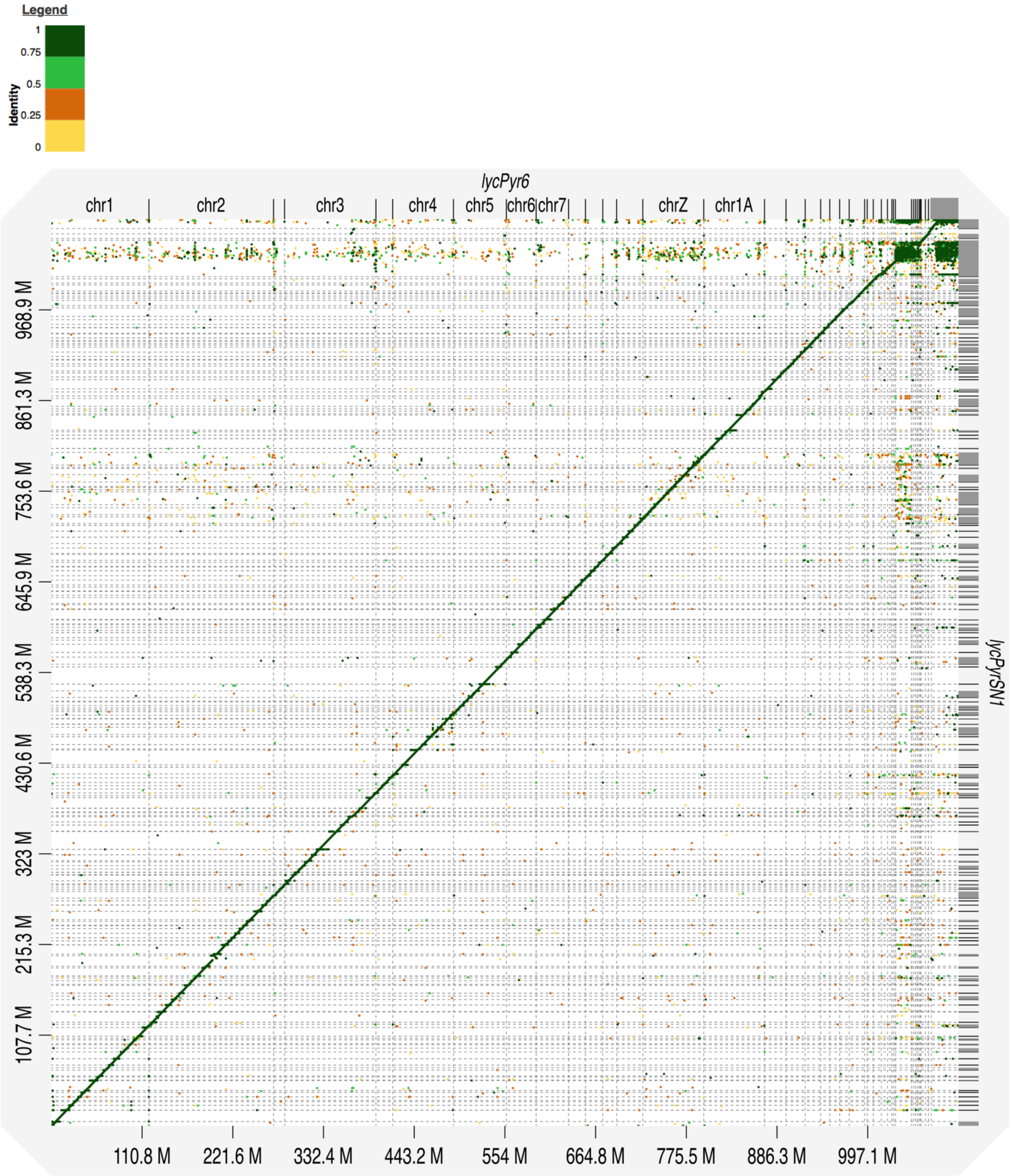

Figure S4. Continued.

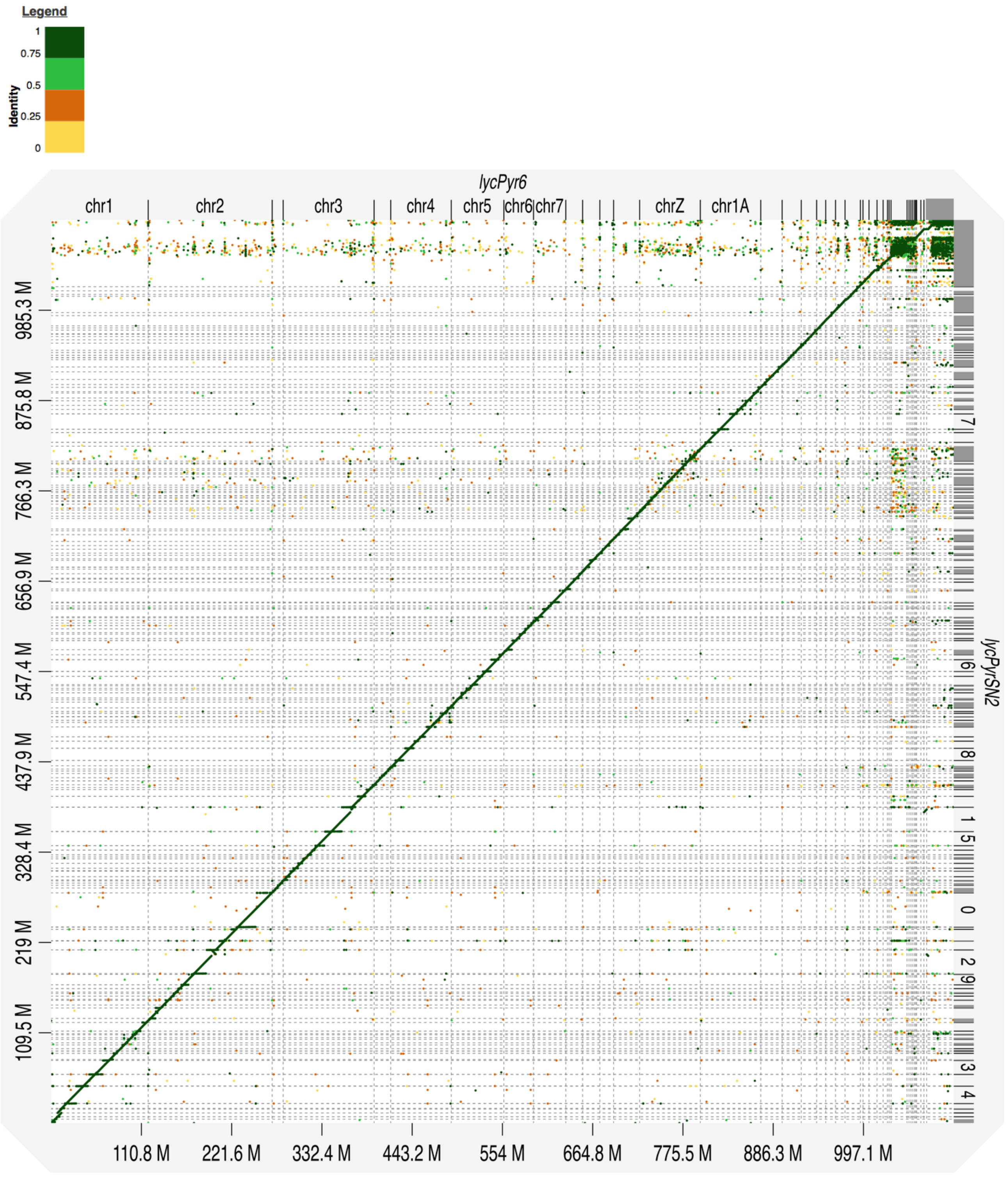

Figure S4. Continued.

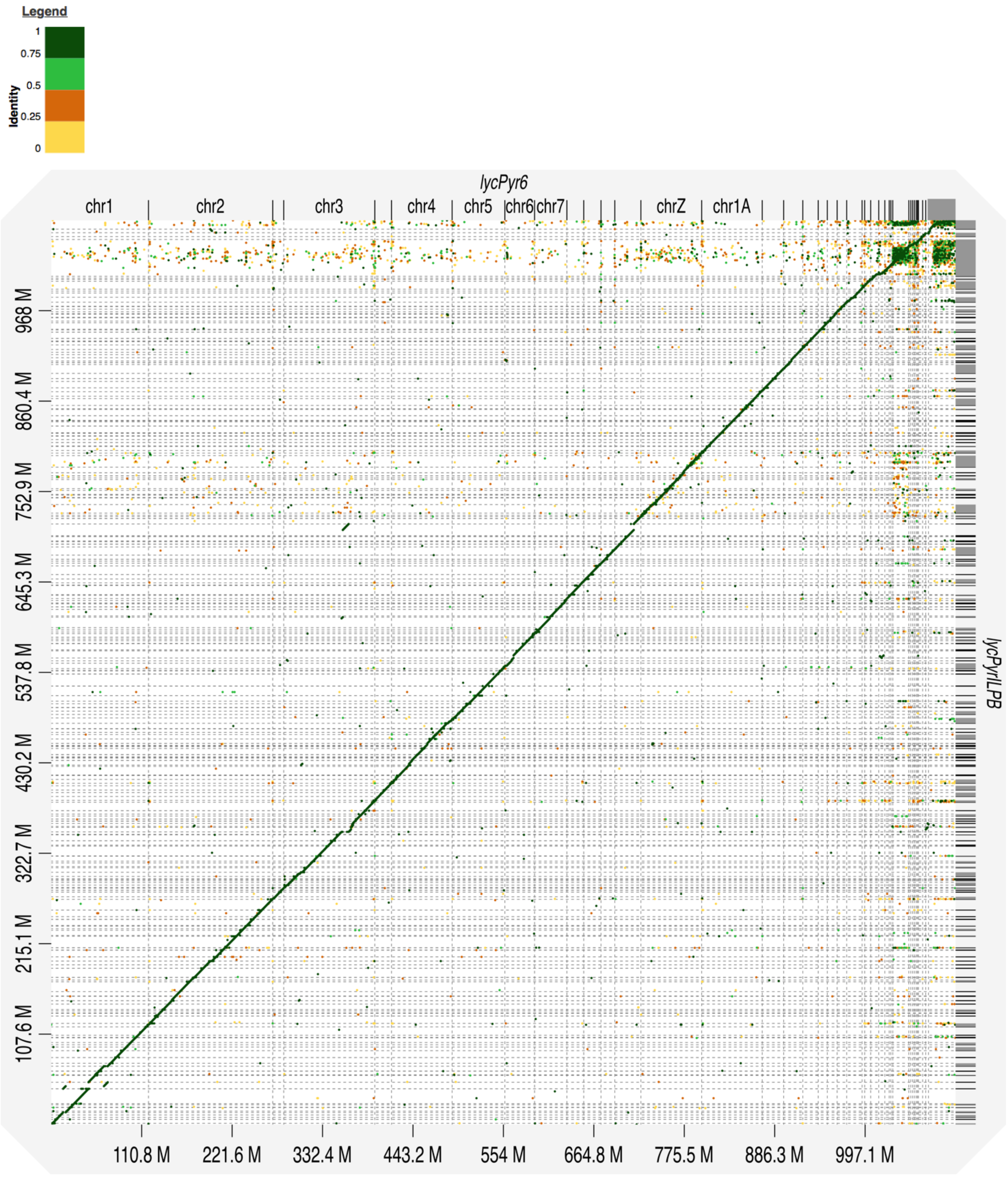

Figure S4. Continued.

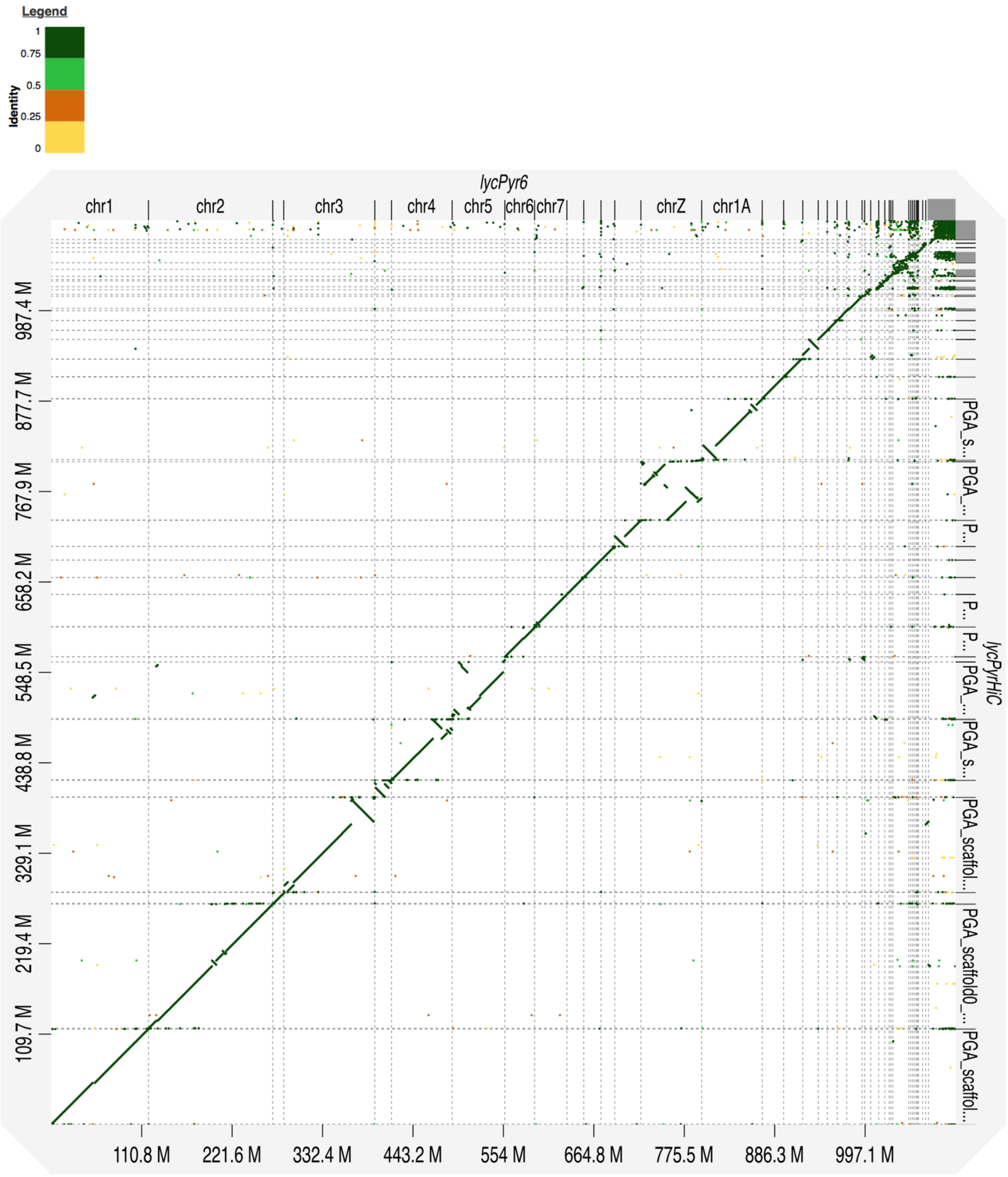

Figure S4. Continued.

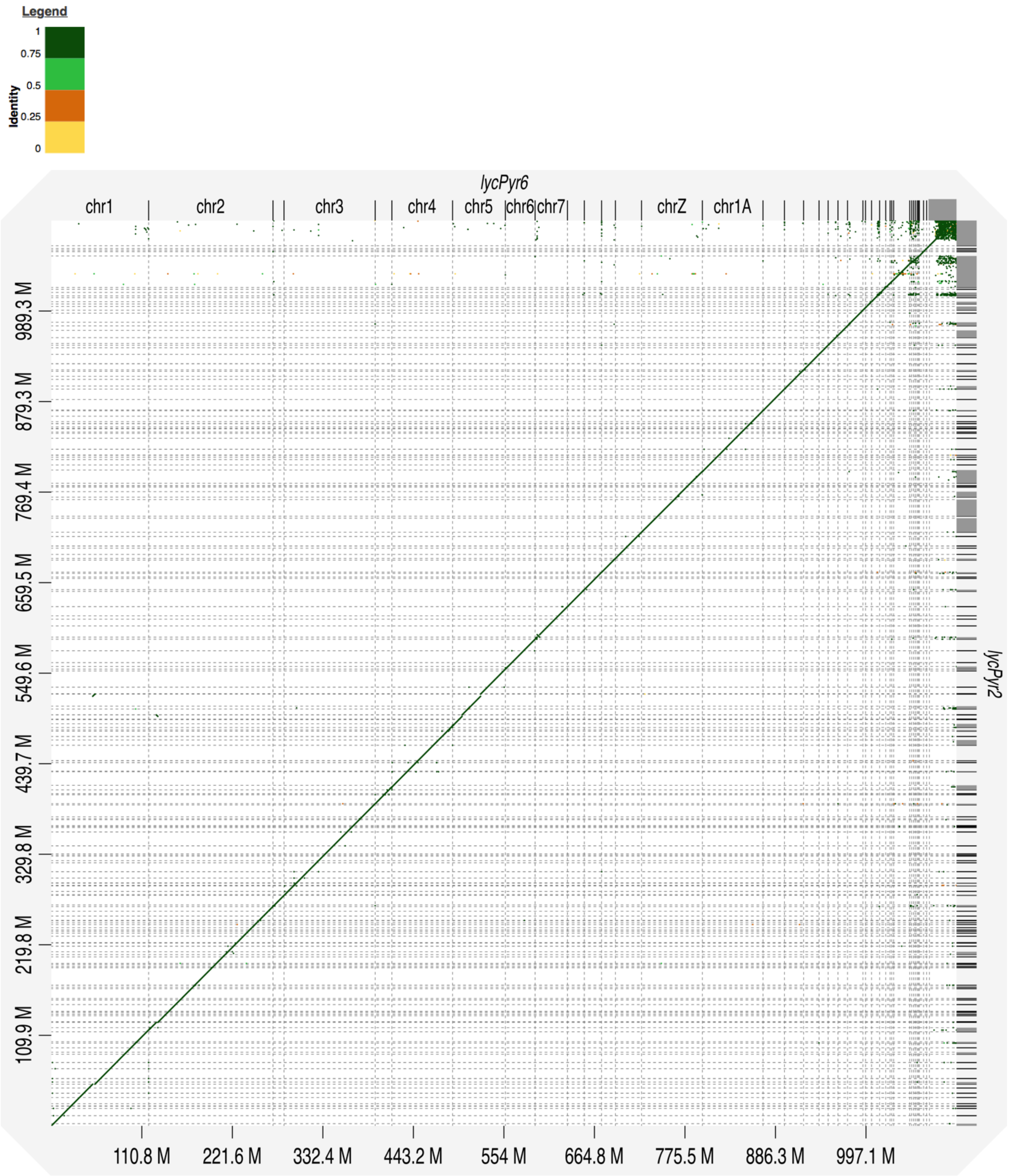

Figure S4. Continued.

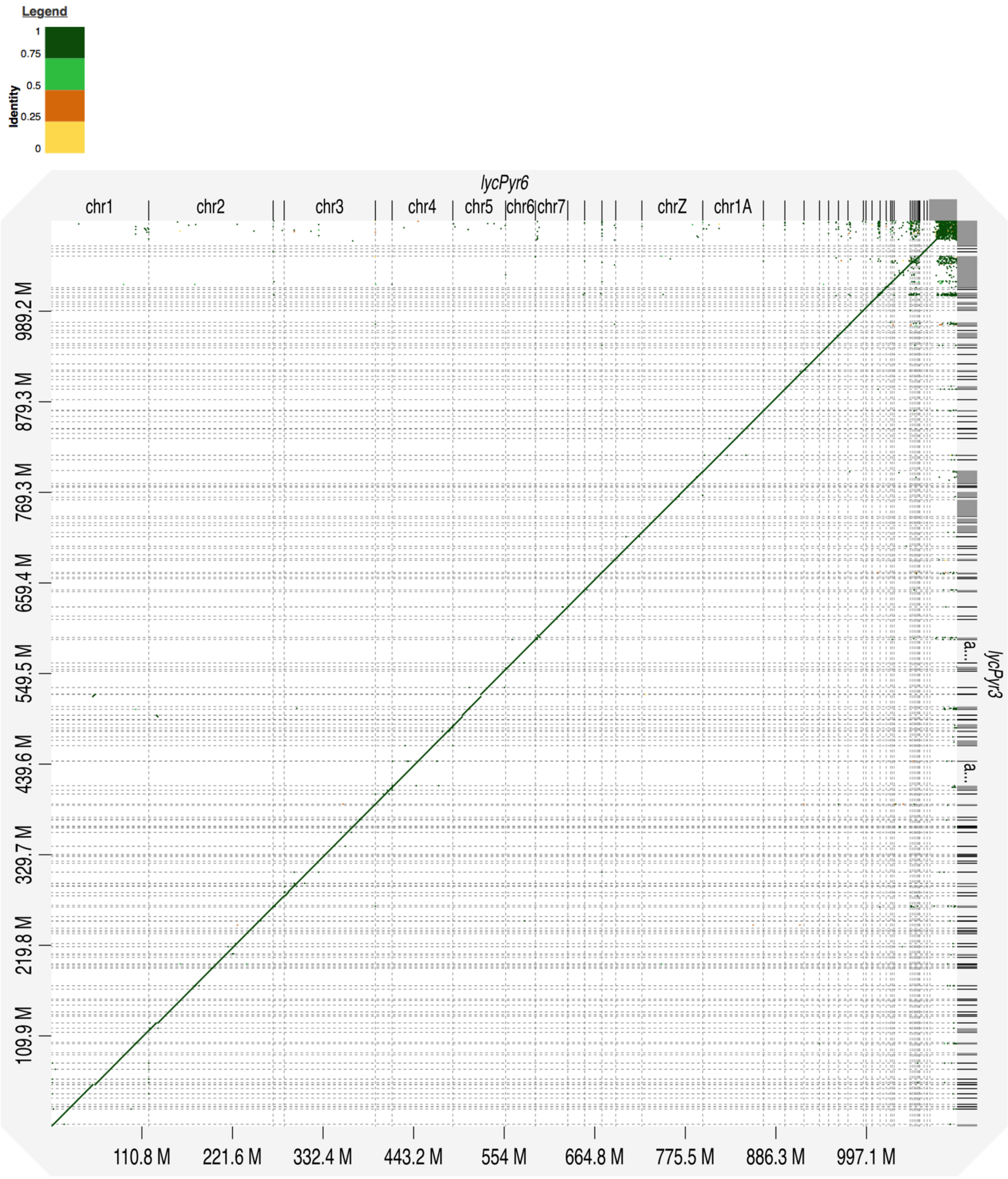

Figure S4. Continued.

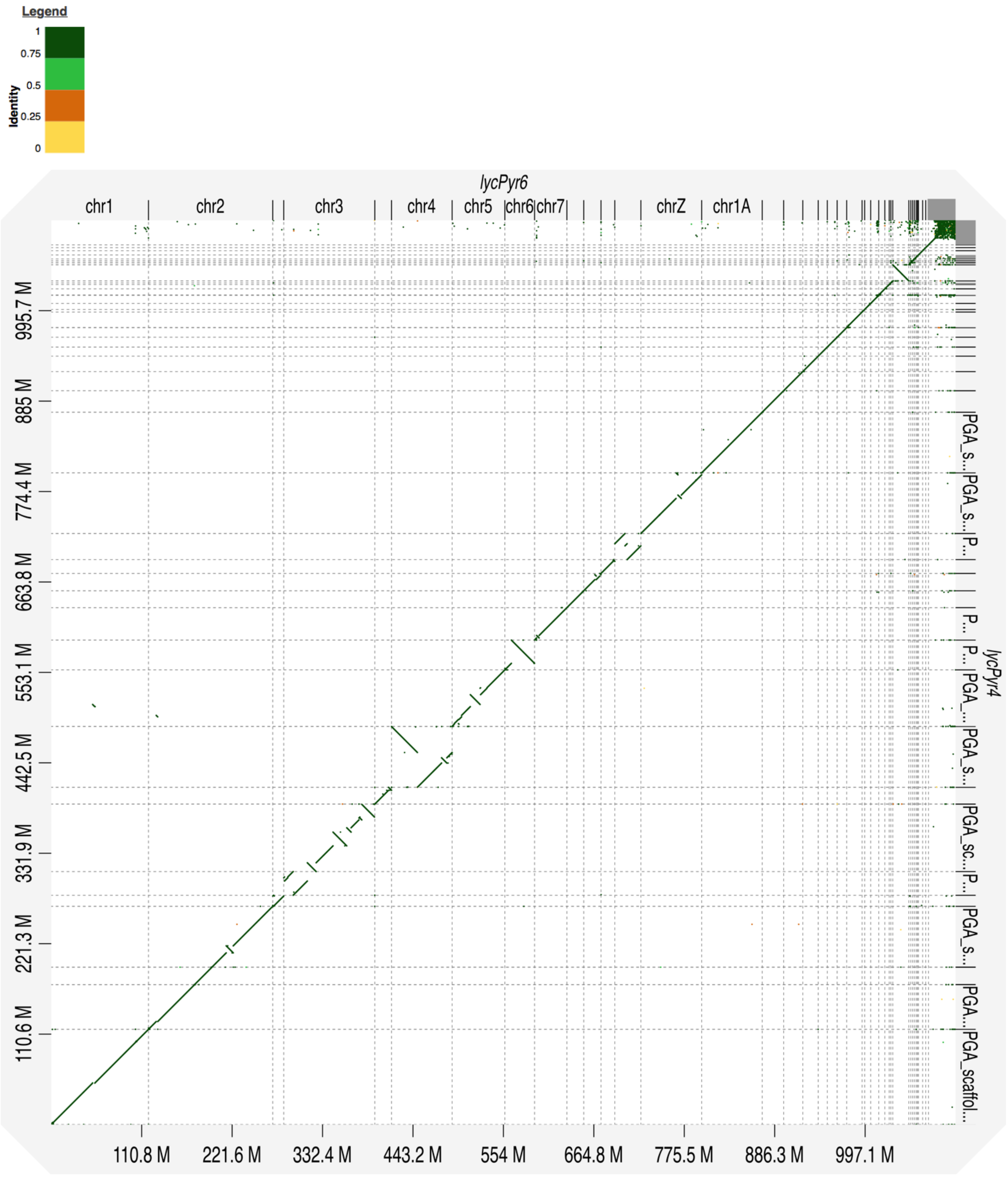

Figure S4. Continued.

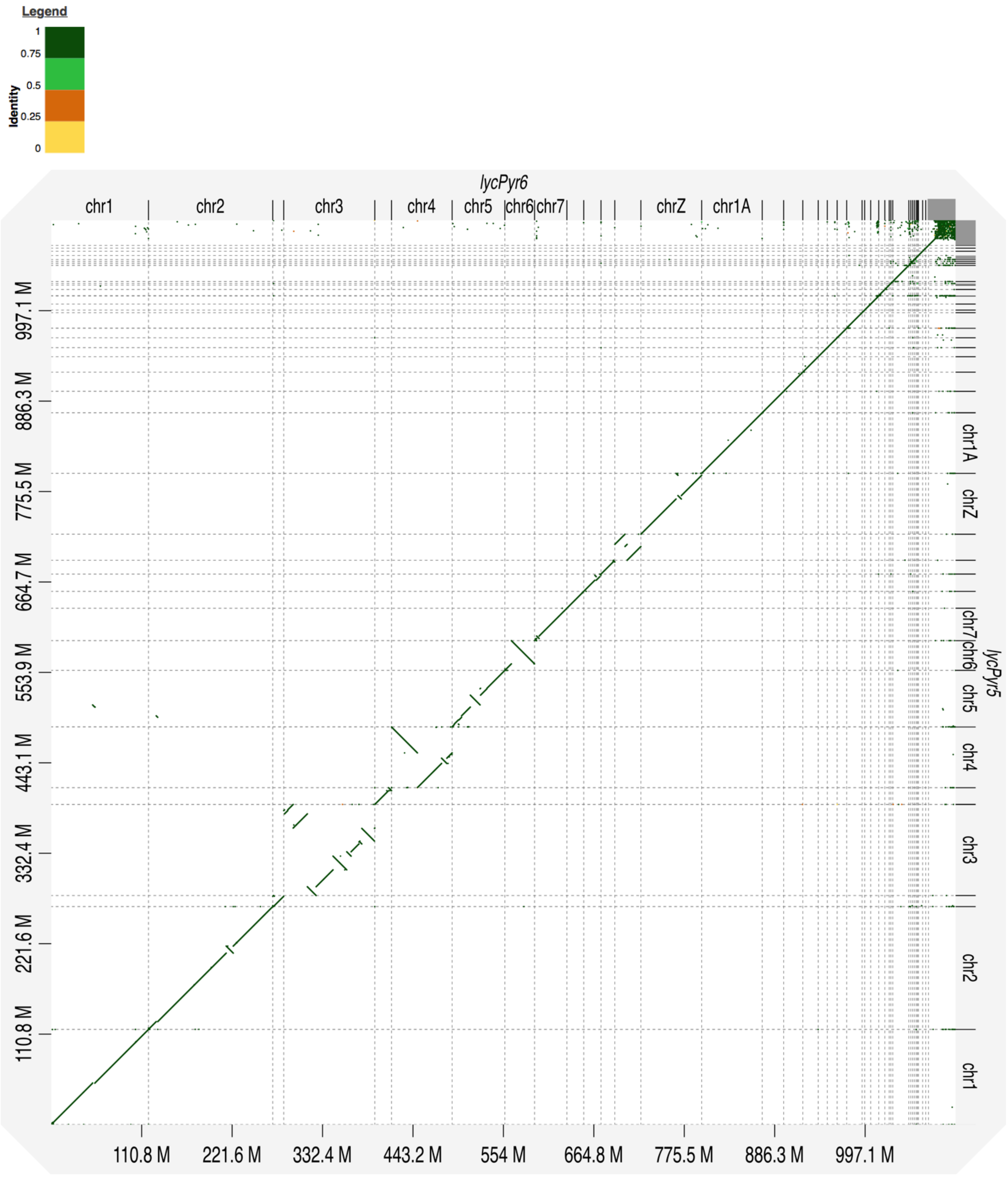

Figure S4. Continued.
