# Supplementary figures and images for: Identifying the causes and consequences of assembly gaps using a multiplatform genome assembly of a bird‐of‐paradise (part 2 of 2)
Source: Mol Ecol Resour. 2020 Oct 10;21(1):263–86. doi: 10.1111/1755-0998.13252 (PMC7757076; doi:10.1111/1755-0998.13252)

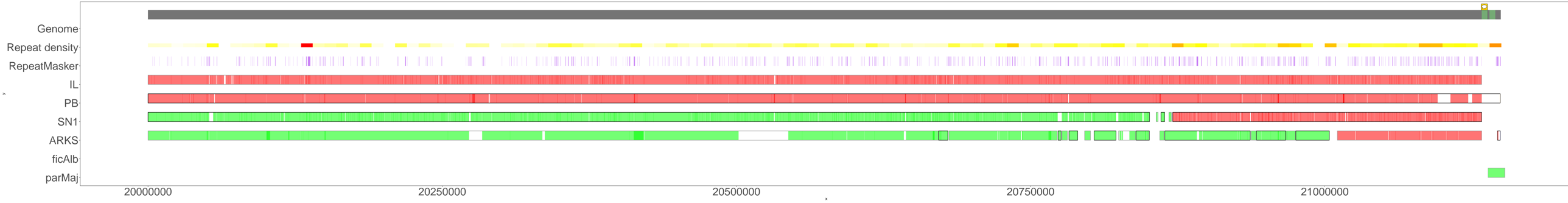

Supplement: Supplementary file 3 — Figure S8 [file MEN-21-263-s003.zip › PGA_scaffold14_plot_3.pdf]

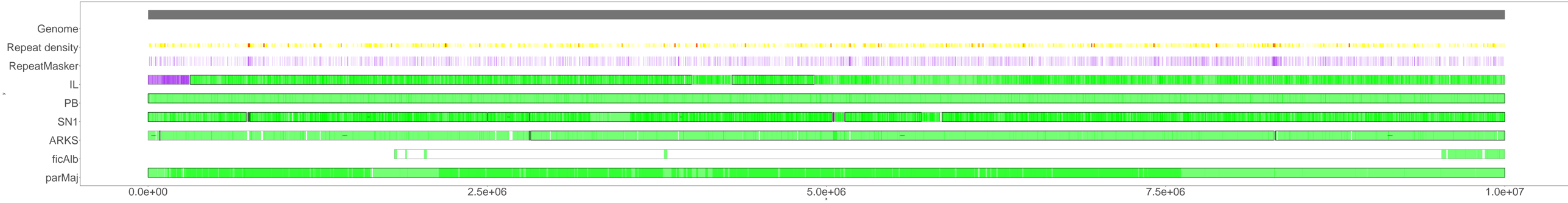

Supplement: Supplementary file 3 — Figure S8 [file MEN-21-263-s003.zip › PGA_scaffold15_plot_1.pdf]

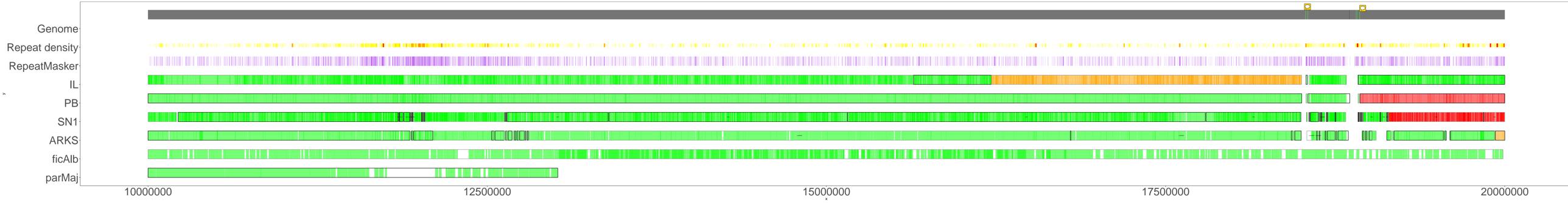

Supplement: Supplementary file 3 — Figure S8 [file MEN-21-263-s003.zip › PGA_scaffold15_plot_2.pdf]

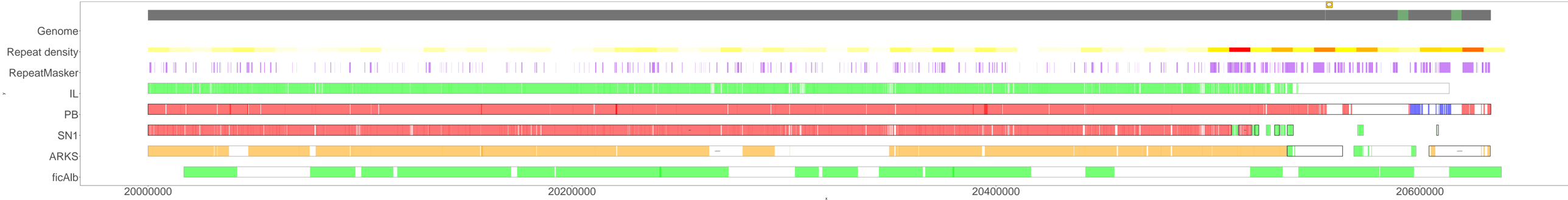

Supplement: Supplementary file 3 — Figure S8 [file MEN-21-263-s003.zip › PGA_scaffold15_plot_3.pdf]

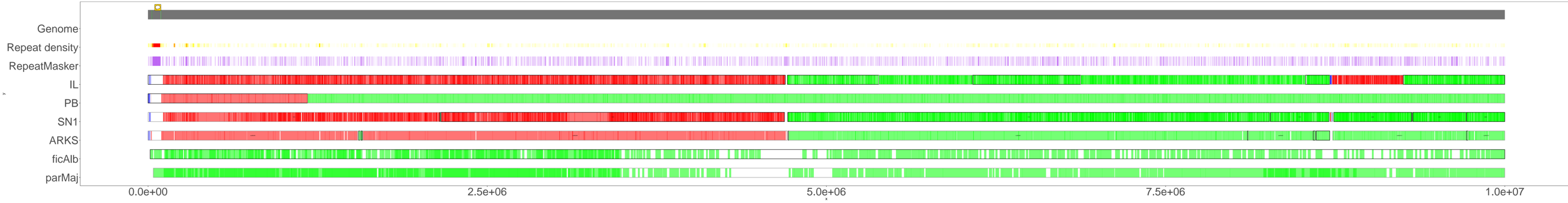

Supplement: Supplementary file 3 — Figure S8 [file MEN-21-263-s003.zip › PGA_scaffold16_plot_1.pdf]

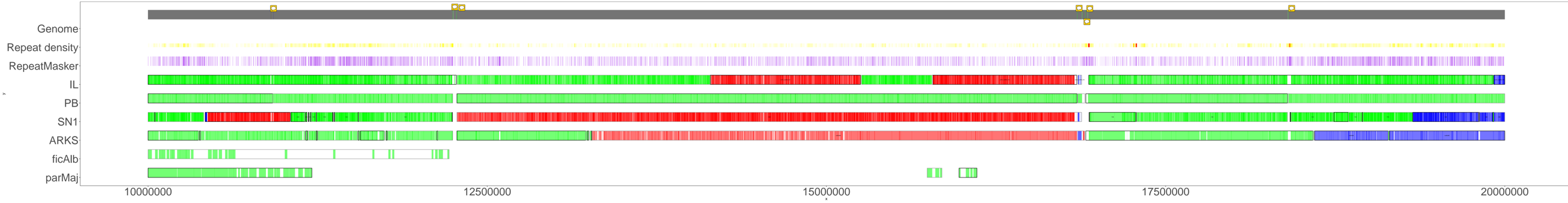

Supplement: Supplementary file 3 — Figure S8 [file MEN-21-263-s003.zip › PGA_scaffold16_plot_2.pdf]

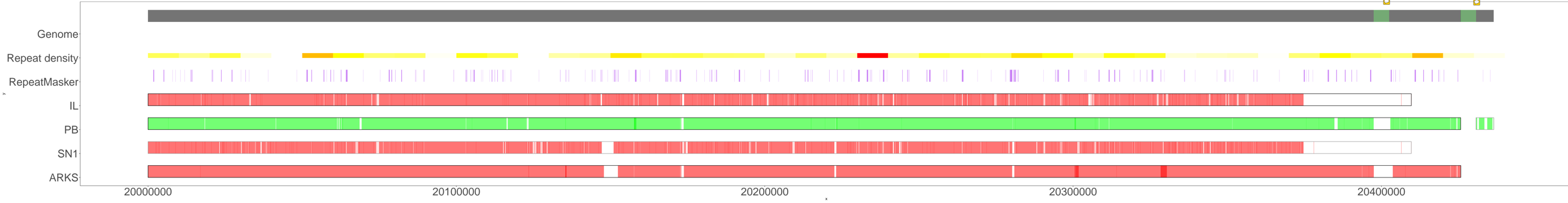

Supplement: Supplementary file 3 — Figure S8 [file MEN-21-263-s003.zip › PGA_scaffold16_plot_3.pdf]

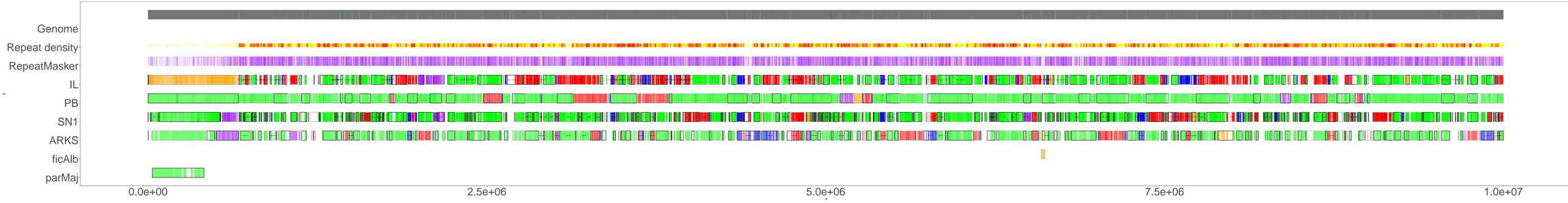

Supplement: Supplementary file 3 — Figure S8 [file MEN-21-263-s003.zip › PGA_scaffold17_plot_1.pdf]

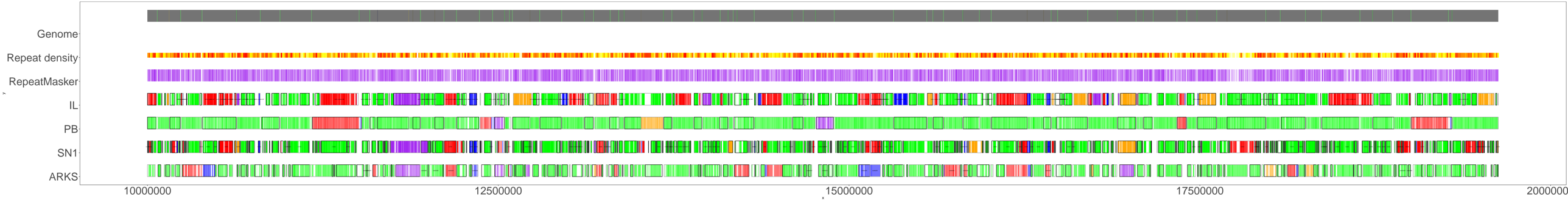

Supplement: Supplementary file 3 — Figure S8 [file MEN-21-263-s003.zip › PGA_scaffold17_plot_2.pdf]

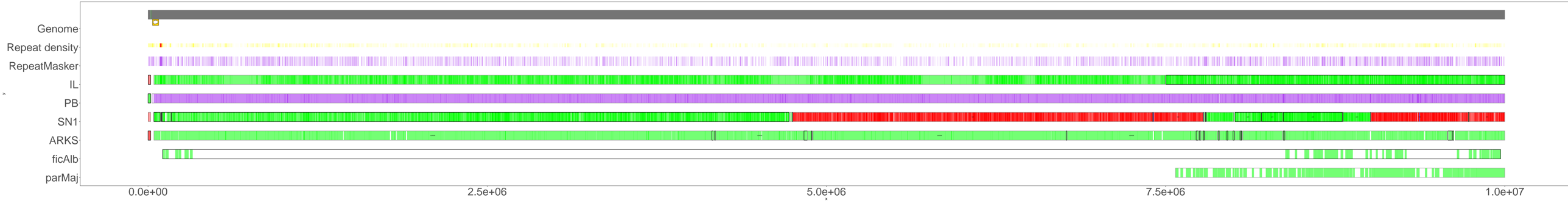

Supplement: Supplementary file 3 — Figure S8 [file MEN-21-263-s003.zip › PGA_scaffold18_plot_1.pdf]

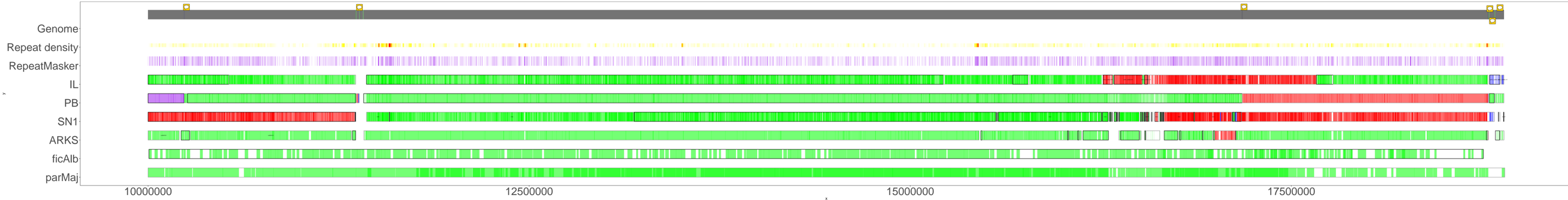

Supplement: Supplementary file 3 — Figure S8 [file MEN-21-263-s003.zip › PGA_scaffold18_plot_2.pdf]

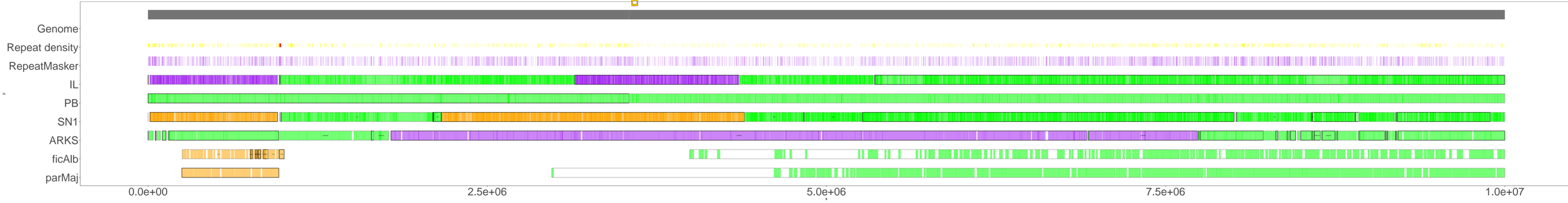

Supplement: Supplementary file 3 — Figure S8 [file MEN-21-263-s003.zip › PGA_scaffold19_plot_1.pdf]

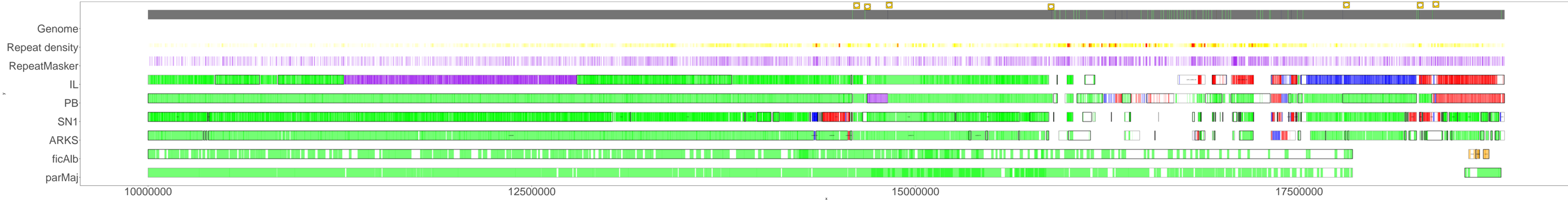

Supplement: Supplementary file 3 — Figure S8 [file MEN-21-263-s003.zip › PGA_scaffold19_plot_2.pdf]

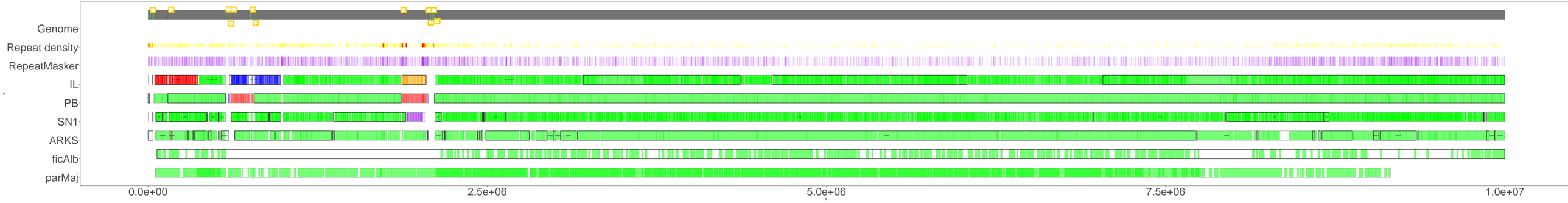

Supplement: Supplementary file 3 — Figure S8 [file MEN-21-263-s003.zip › PGA_scaffold20_plot_1.pdf]

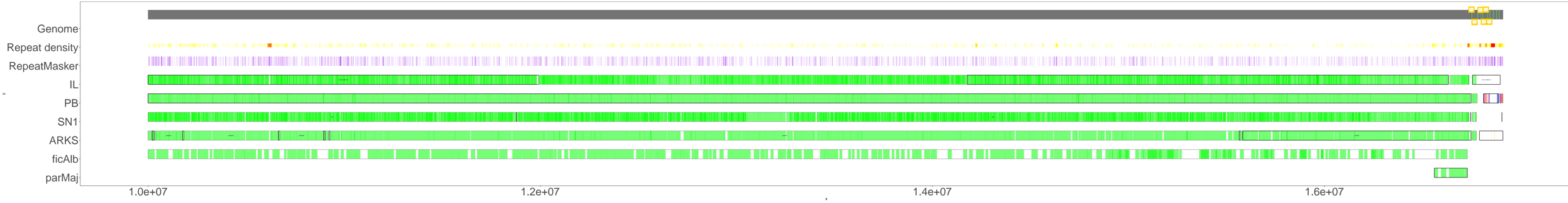

Supplement: Supplementary file 3 — Figure S8 [file MEN-21-263-s003.zip › PGA_scaffold20_plot_2.pdf]

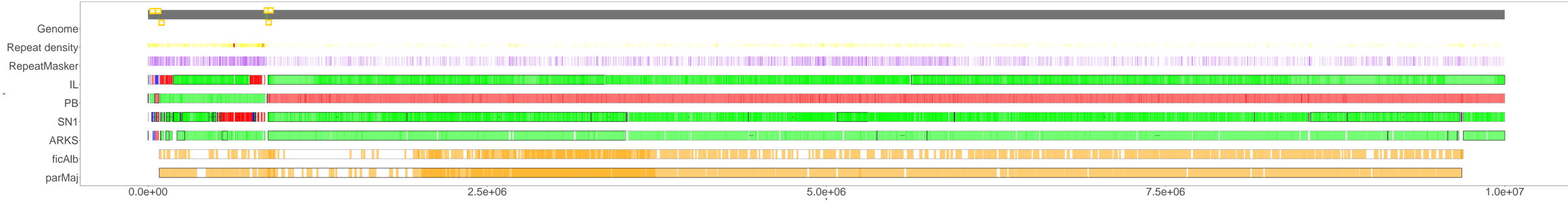

Supplement: Supplementary file 3 — Figure S8 [file MEN-21-263-s003.zip › PGA_scaffold21_plot_1.pdf]

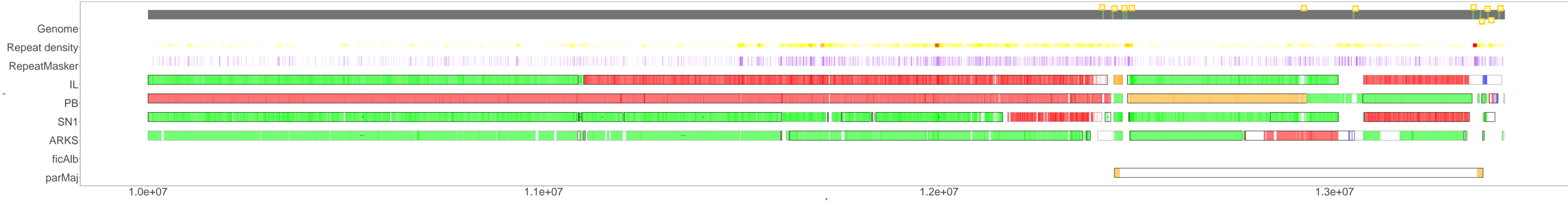

Supplement: Supplementary file 3 — Figure S8 [file MEN-21-263-s003.zip › PGA_scaffold21_plot_2.pdf]

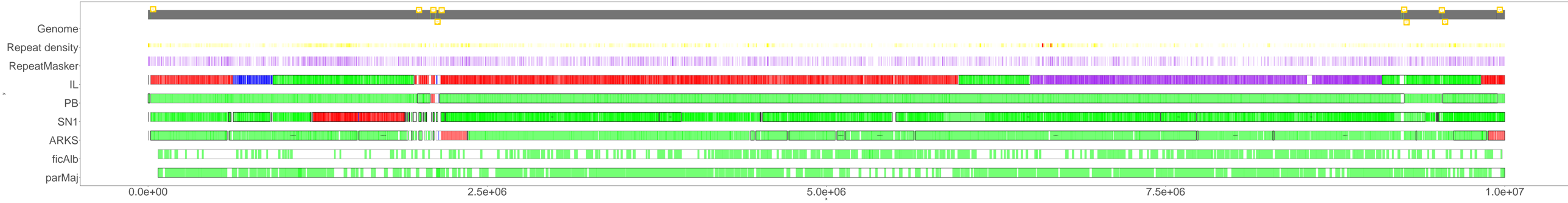

Supplement: Supplementary file 3 — Figure S8 [file MEN-21-263-s003.zip › PGA_scaffold22_plot_1.pdf]

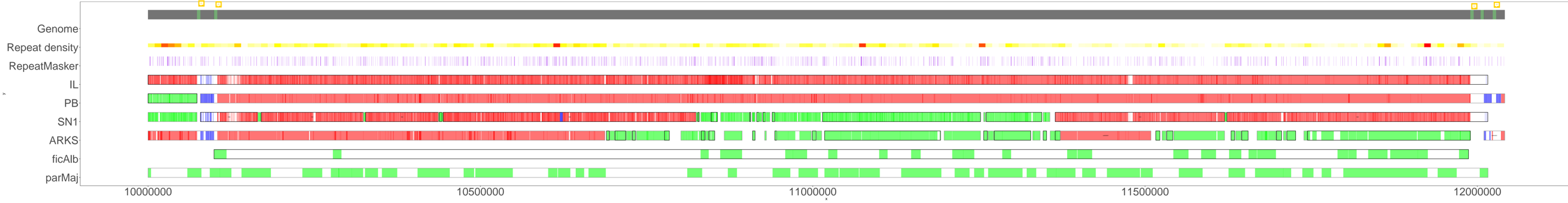

Supplement: Supplementary file 3 — Figure S8 [file MEN-21-263-s003.zip › PGA_scaffold22_plot_2.pdf]

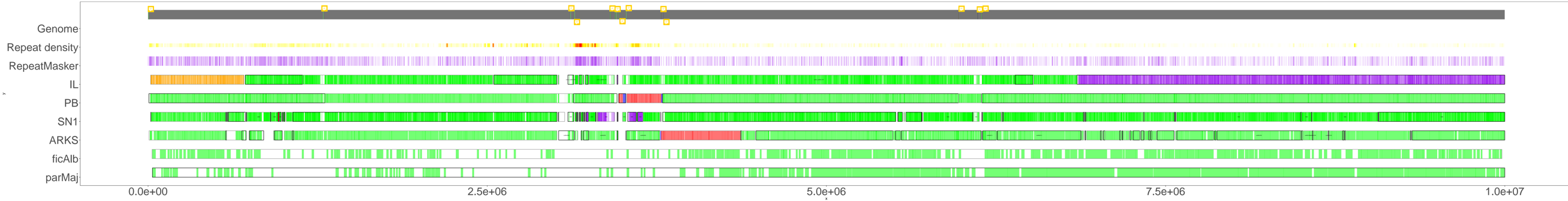

Supplement: Supplementary file 3 — Figure S8 [file MEN-21-263-s003.zip › PGA_scaffold23_plot_1.pdf]

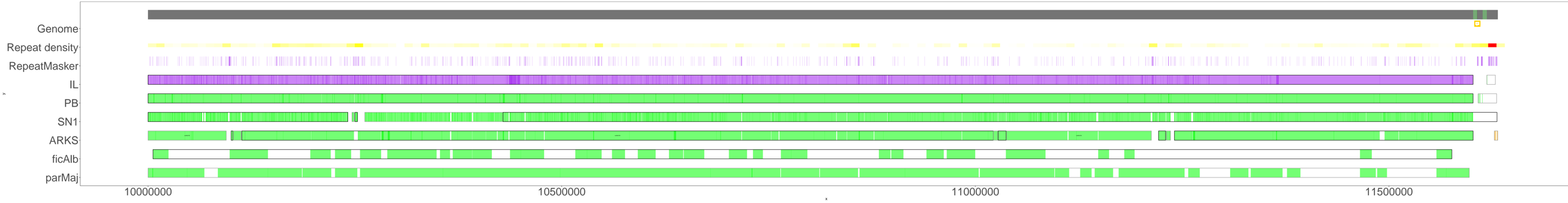

Supplement: Supplementary file 3 — Figure S8 [file MEN-21-263-s003.zip › PGA_scaffold23_plot_2.pdf]

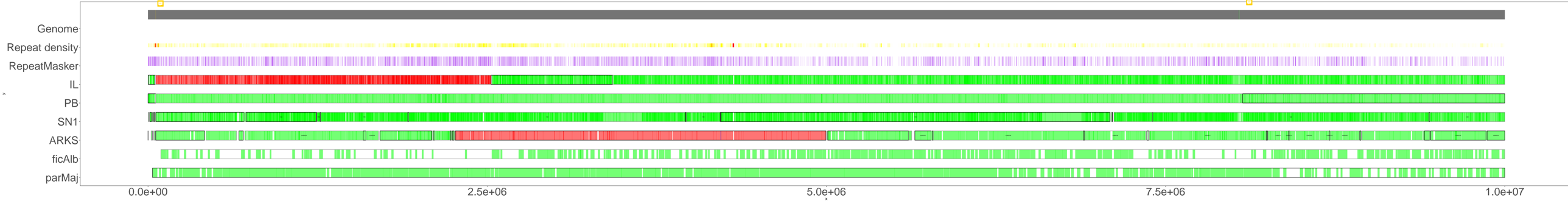

Supplement: Supplementary file 3 — Figure S8 [file MEN-21-263-s003.zip › PGA_scaffold24_plot_1.pdf]

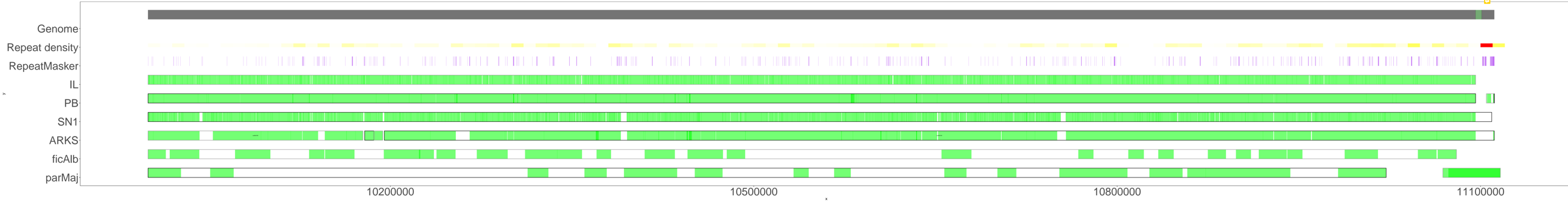

Supplement: Supplementary file 3 — Figure S8 [file MEN-21-263-s003.zip › PGA_scaffold24_plot_2.pdf]

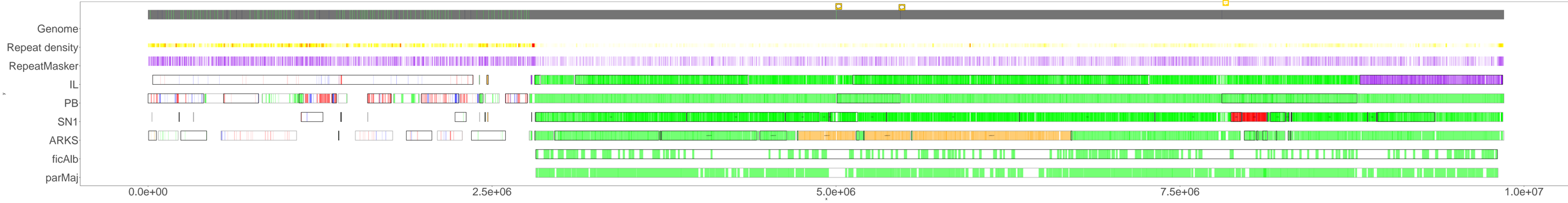

Supplement: Supplementary file 3 — Figure S8 [file MEN-21-263-s003.zip › PGA_scaffold25_plot_1.pdf]

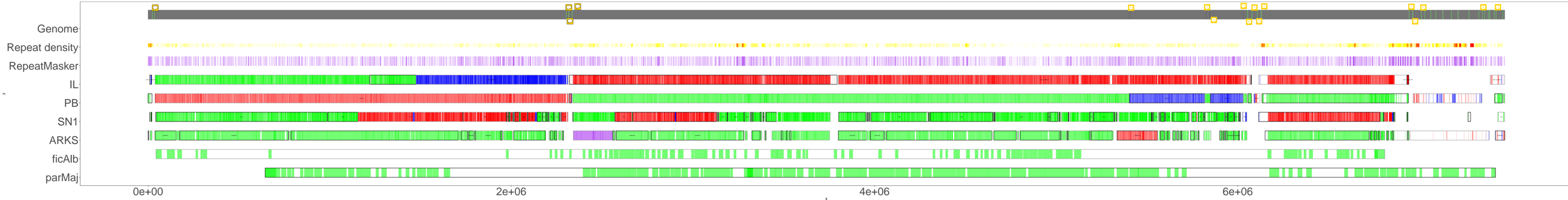

Supplement: Supplementary file 3 — Figure S8 [file MEN-21-263-s003.zip › PGA_scaffold26_plot_1.pdf]
