# Supplementary figures and images for: Identifying the causes and consequences of assembly gaps using a multiplatform genome assembly of a bird‐of‐paradise (part 1 of 2)
Source: Mol Ecol Resour. 2020 Oct 10;21(1):263–86. doi: 10.1111/1755-0998.13252 (PMC7757076; doi:10.1111/1755-0998.13252)

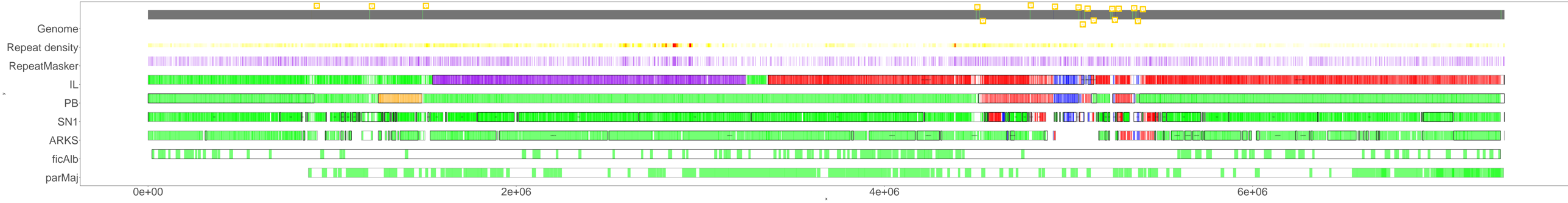

Supplement: Supplementary file 3 — Figure S8 [file MEN-21-263-s003.zip › PGA_scaffold27_plot_1.pdf]

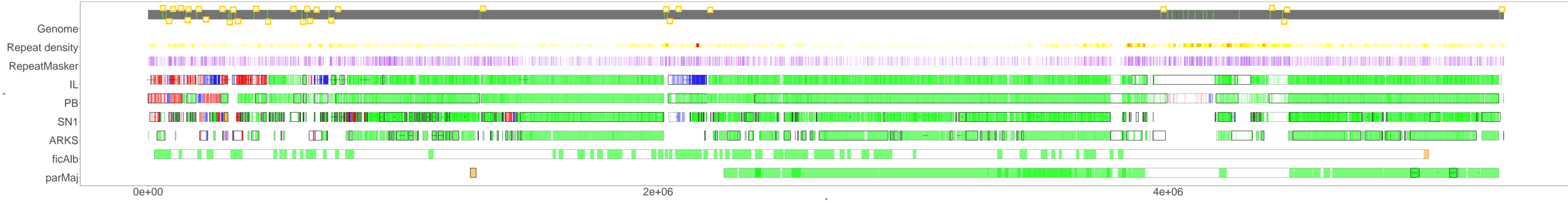

Supplement: Supplementary file 3 — Figure S8 [file MEN-21-263-s003.zip › PGA_scaffold28_plot_1.pdf]

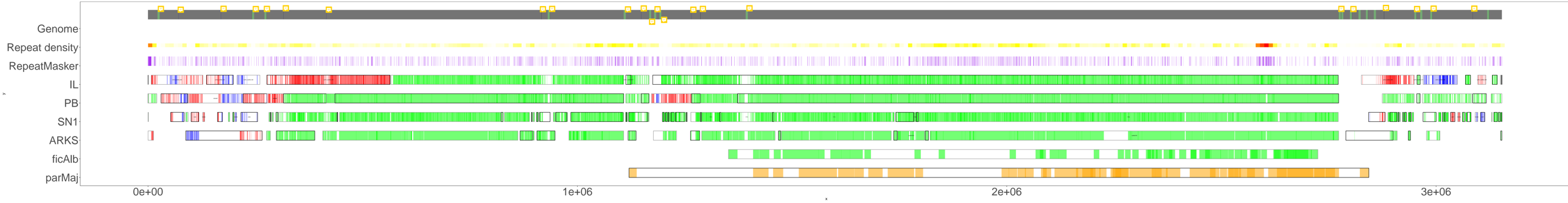

Supplement: Supplementary file 3 — Figure S8 [file MEN-21-263-s003.zip › PGA_scaffold29_plot_1.pdf]

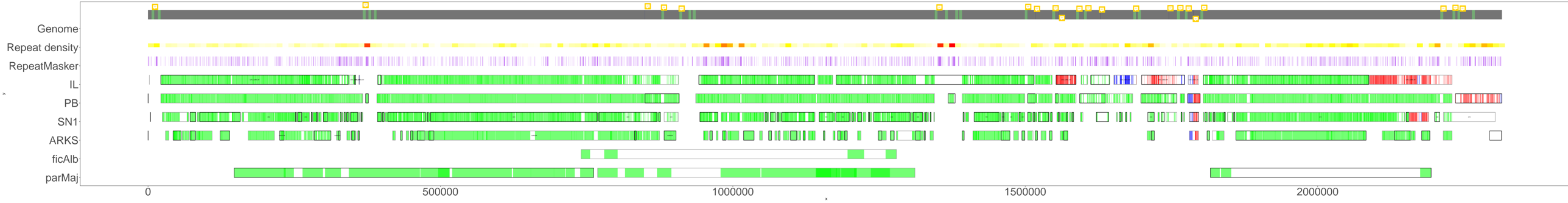

Supplement: Supplementary file 3 — Figure S8 [file MEN-21-263-s003.zip › PGA_scaffold30_plot_1.pdf]

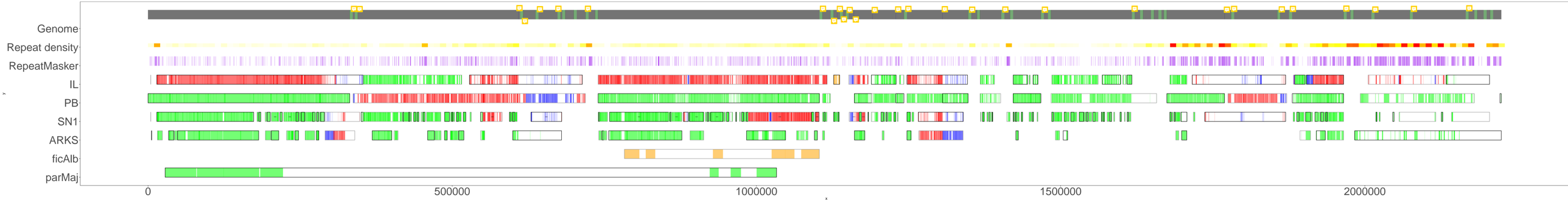

Supplement: Supplementary file 3 — Figure S8 [file MEN-21-263-s003.zip › PGA_scaffold31_plot_1.pdf]

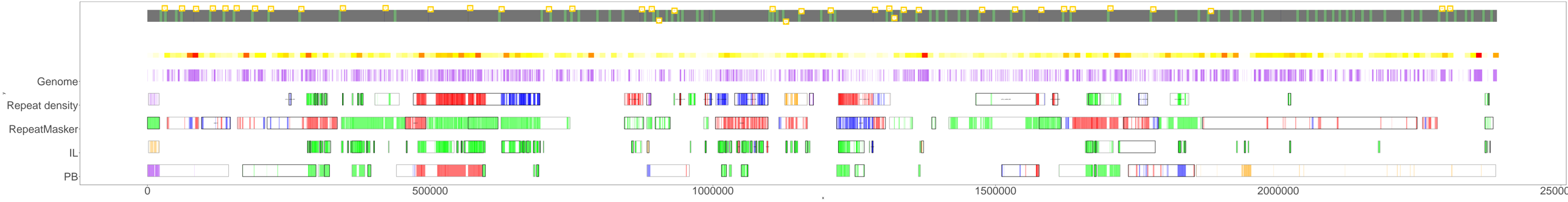

Supplement: Supplementary file 3 — Figure S8 [file MEN-21-263-s003.zip › PGA_scaffold32_plot_1.pdf]

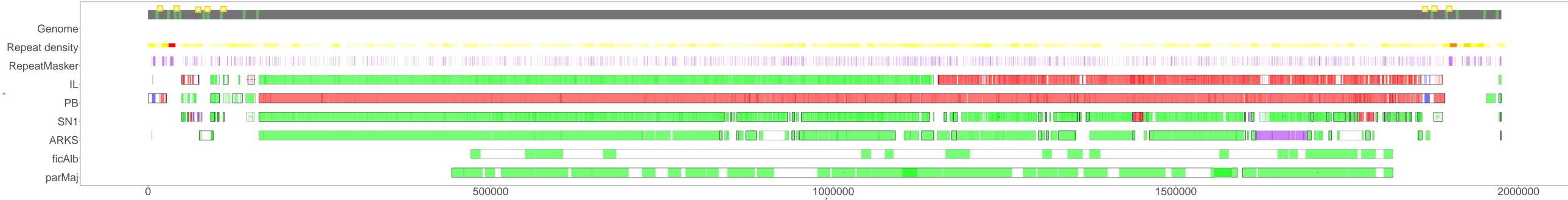

Supplement: Supplementary file 3 — Figure S8 [file MEN-21-263-s003.zip › PGA_scaffold33_plot_1.pdf]

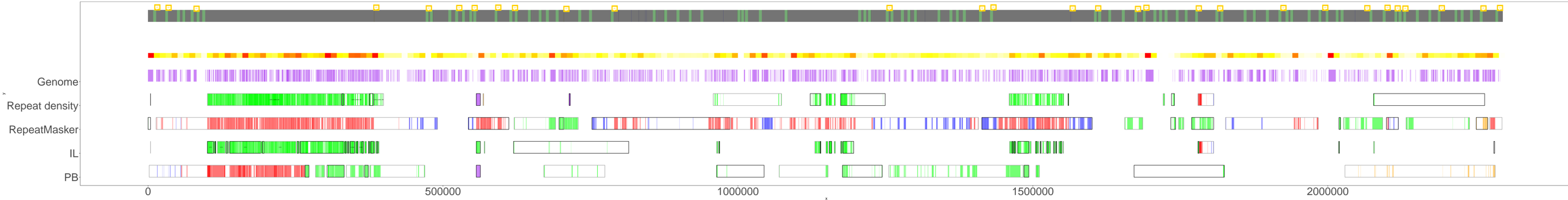

Supplement: Supplementary file 3 — Figure S8 [file MEN-21-263-s003.zip › PGA_scaffold34_plot_1.pdf]

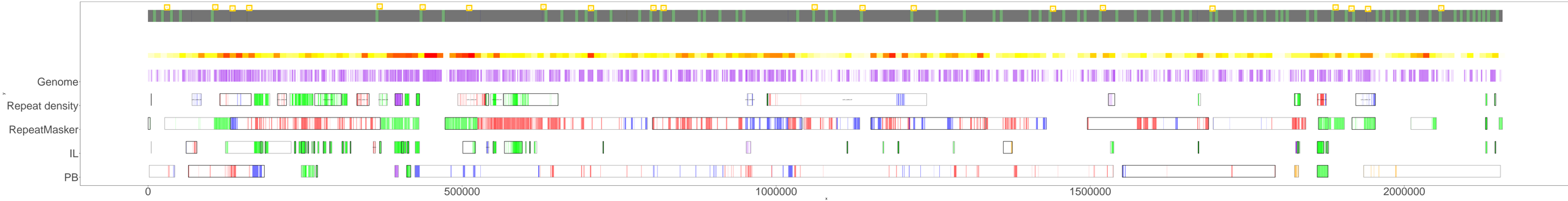

Supplement: Supplementary file 3 — Figure S8 [file MEN-21-263-s003.zip › PGA_scaffold35_plot_1.pdf]

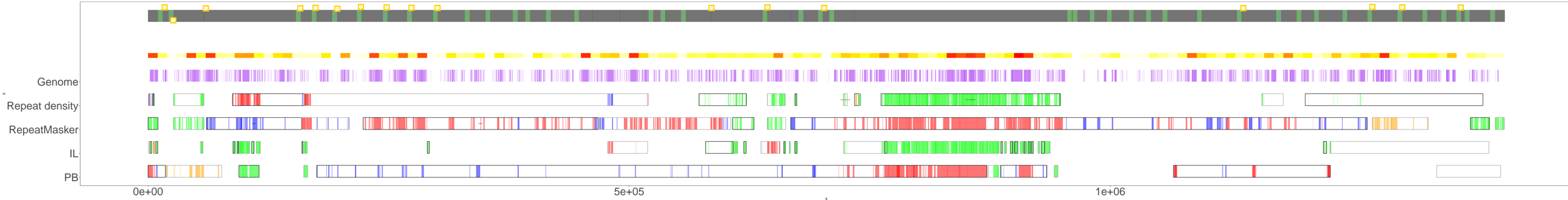

Supplement: Supplementary file 3 — Figure S8 [file MEN-21-263-s003.zip › PGA_scaffold36_plot_1.pdf]

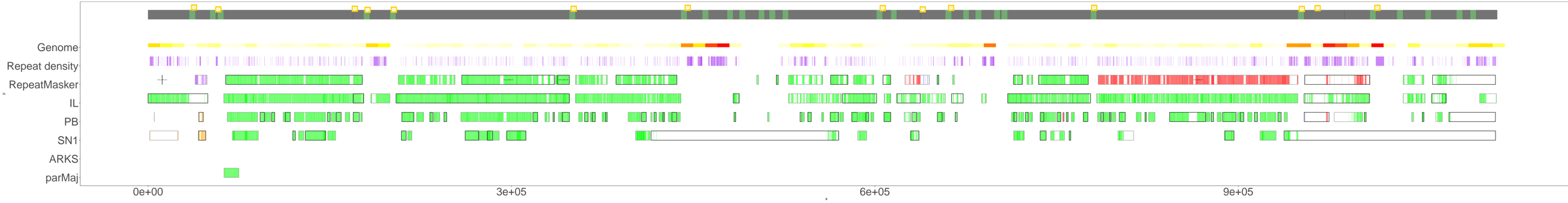

Supplement: Supplementary file 3 — Figure S8 [file MEN-21-263-s003.zip › PGA_scaffold37_plot_1.pdf]

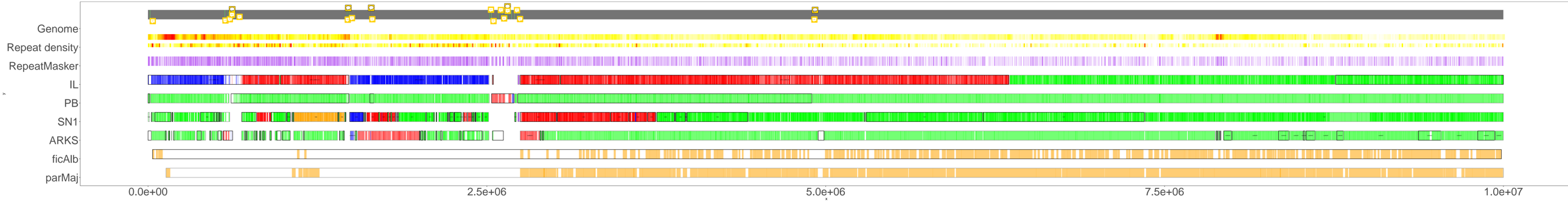

Supplement: Supplementary file 3 — Figure S8 [file MEN-21-263-s003.zip › PGA_scaffold0_plot_1.pdf]

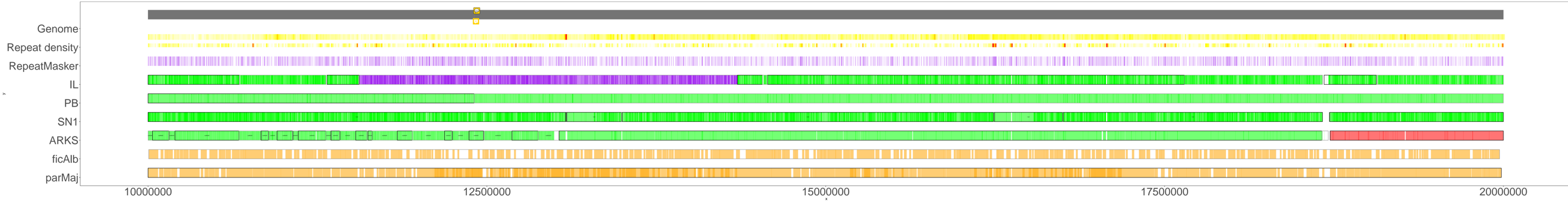

Supplement: Supplementary file 3 — Figure S8 [file MEN-21-263-s003.zip › PGA_scaffold0_plot_2.pdf]

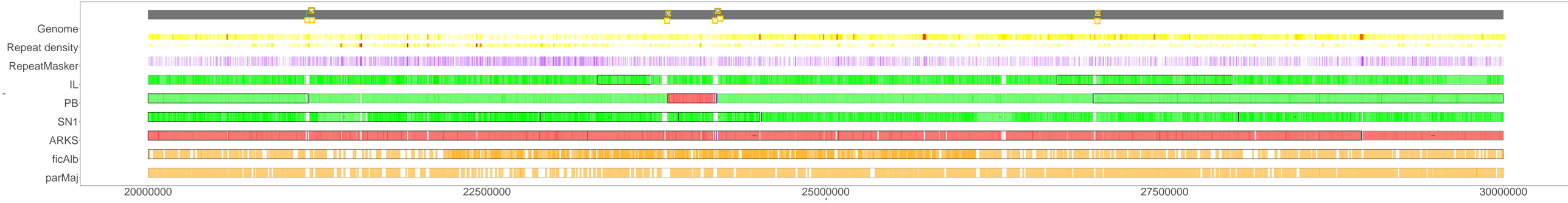

Supplement: Supplementary file 3 — Figure S8 [file MEN-21-263-s003.zip › PGA_scaffold0_plot_3.pdf]

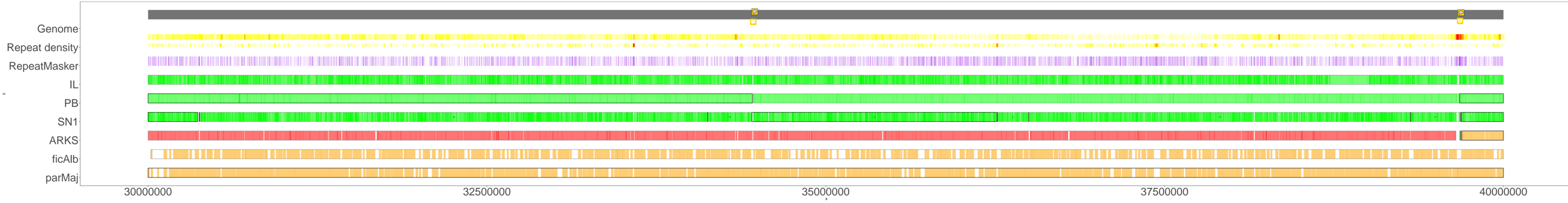

Supplement: Supplementary file 3 — Figure S8 [file MEN-21-263-s003.zip › PGA_scaffold0_plot_4.pdf]

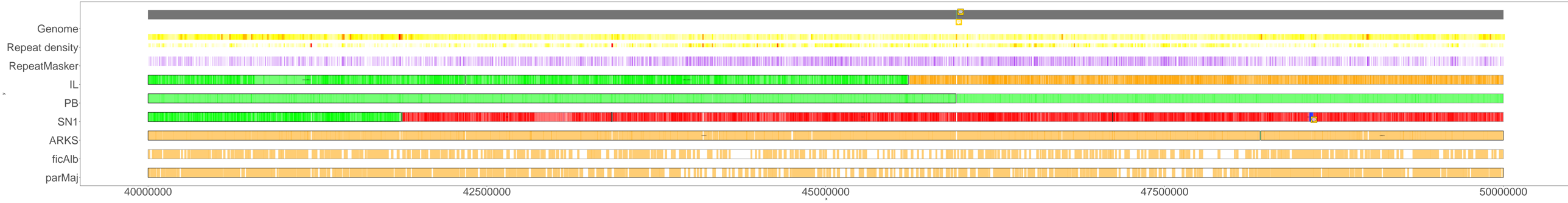

Supplement: Supplementary file 3 — Figure S8 [file MEN-21-263-s003.zip › PGA_scaffold0_plot_5.pdf]

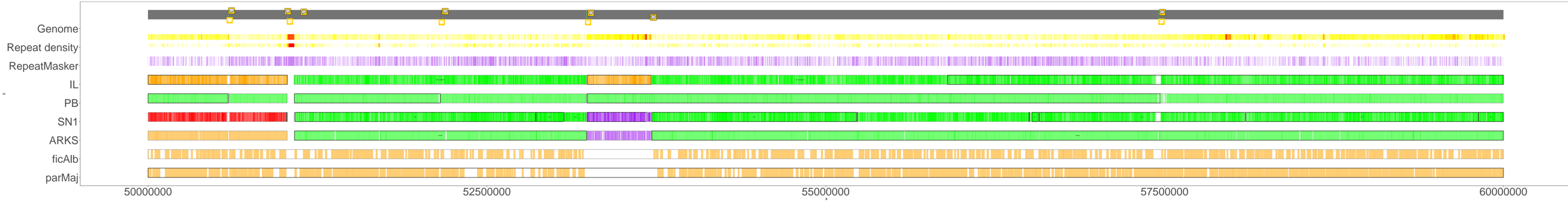

Supplement: Supplementary file 3 — Figure S8 [file MEN-21-263-s003.zip › PGA_scaffold0_plot_6.pdf]

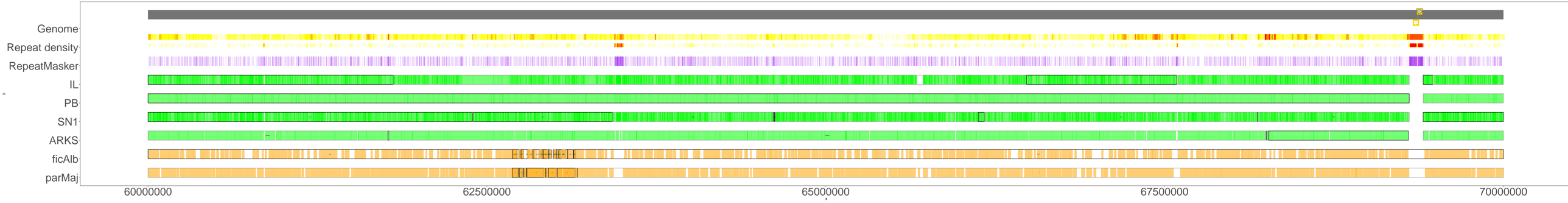

Supplement: Supplementary file 3 — Figure S8 [file MEN-21-263-s003.zip › PGA_scaffold0_plot_7.pdf]

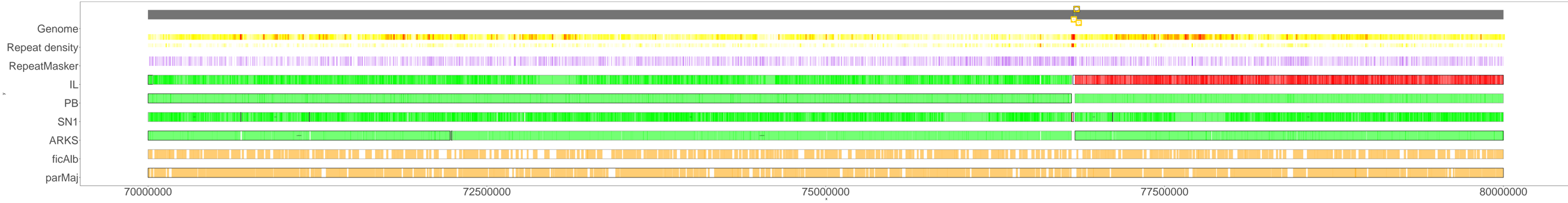

Supplement: Supplementary file 3 — Figure S8 [file MEN-21-263-s003.zip › PGA_scaffold0_plot_8.pdf]

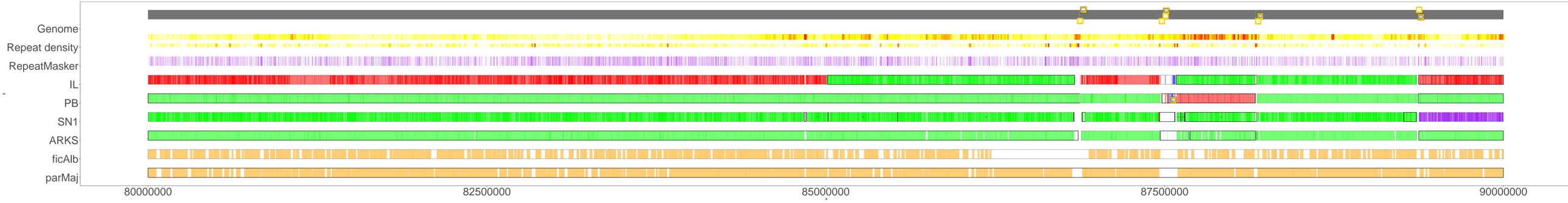

Supplement: Supplementary file 3 — Figure S8 [file MEN-21-263-s003.zip › PGA_scaffold0_plot_9.pdf]

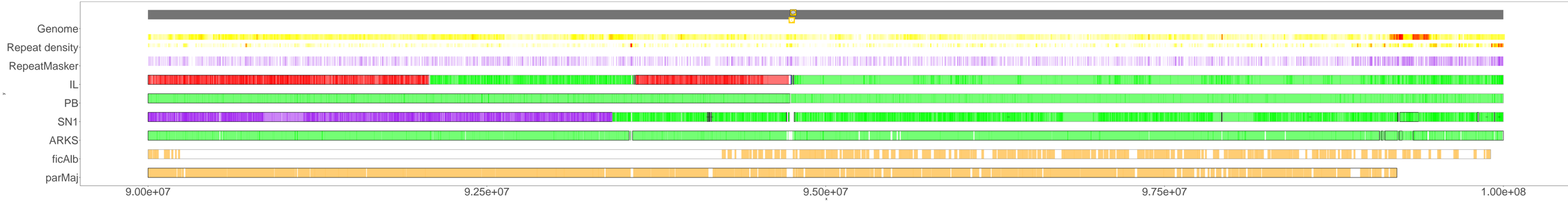

Supplement: Supplementary file 3 — Figure S8 [file MEN-21-263-s003.zip › PGA_scaffold0_plot_10.pdf]

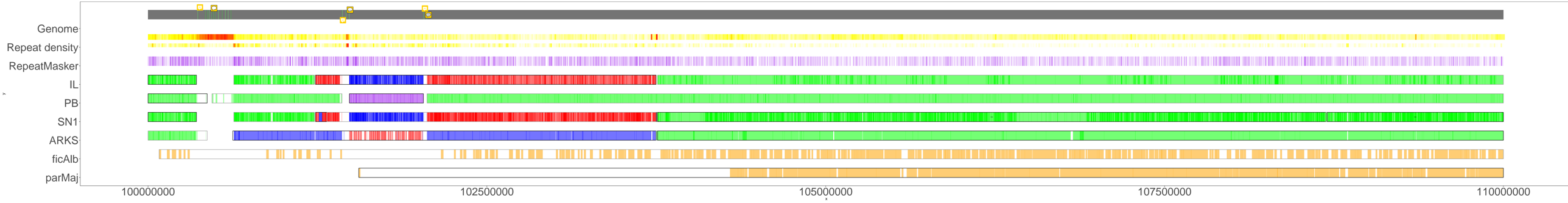

Supplement: Supplementary file 3 — Figure S8 [file MEN-21-263-s003.zip › PGA_scaffold0_plot_11.pdf]

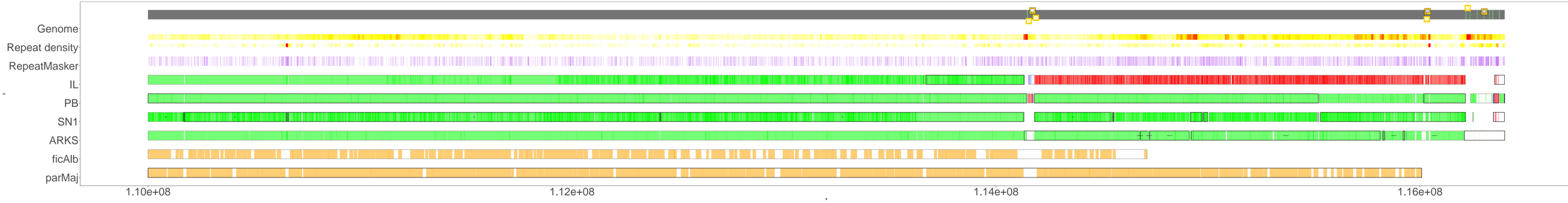

Supplement: Supplementary file 3 — Figure S8 [file MEN-21-263-s003.zip › PGA_scaffold0_plot_12.pdf]

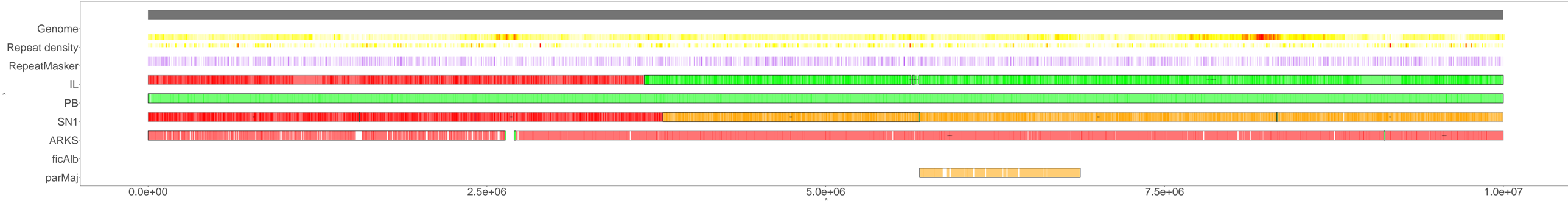

Supplement: Supplementary file 3 — Figure S8 [file MEN-21-263-s003.zip › PGA_scaffold1_plot_1.pdf]

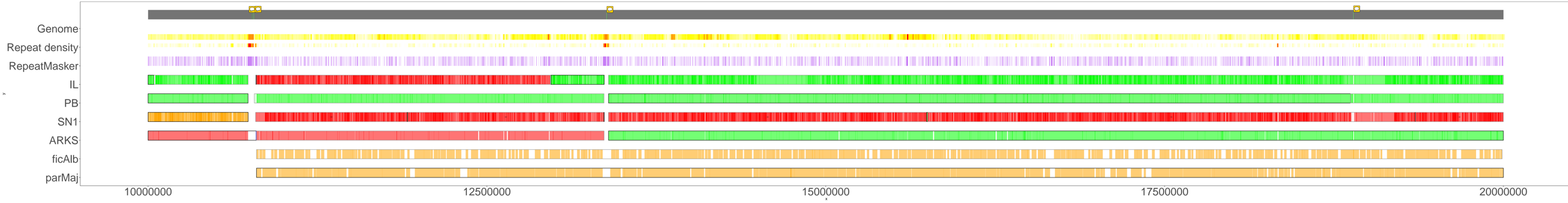

Supplement: Supplementary file 3 — Figure S8 [file MEN-21-263-s003.zip › PGA_scaffold1_plot_2.pdf]

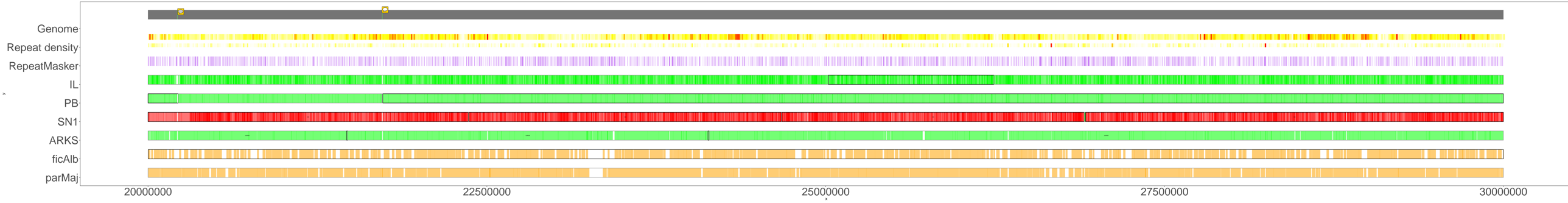

Supplement: Supplementary file 3 — Figure S8 [file MEN-21-263-s003.zip › PGA_scaffold1_plot_3.pdf]

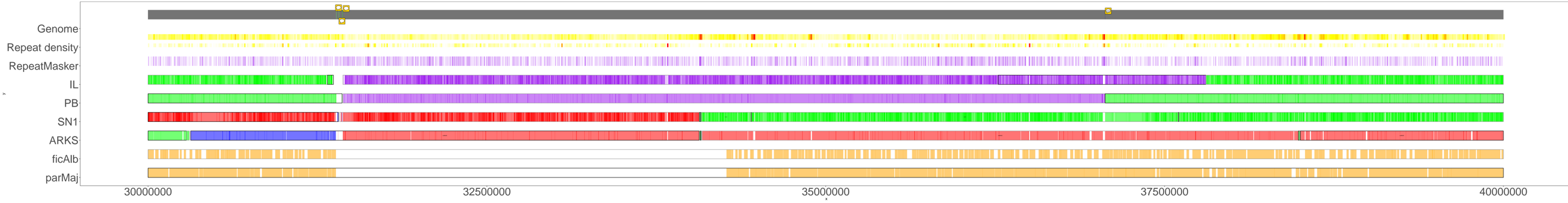

Supplement: Supplementary file 3 — Figure S8 [file MEN-21-263-s003.zip › PGA_scaffold1_plot_4.pdf]

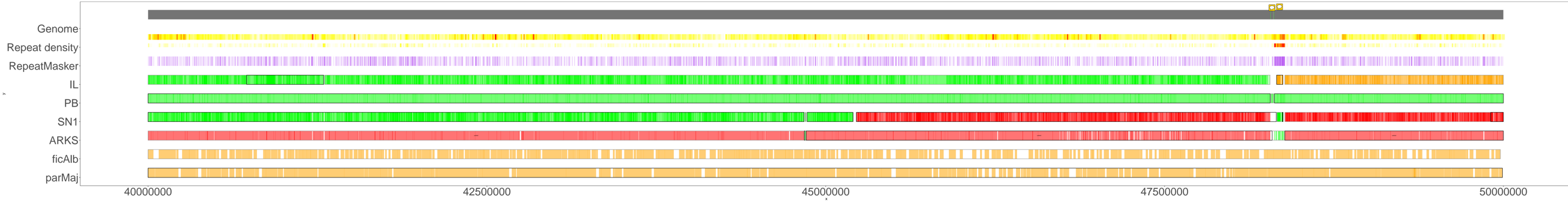

Supplement: Supplementary file 3 — Figure S8 [file MEN-21-263-s003.zip › PGA_scaffold1_plot_5.pdf]

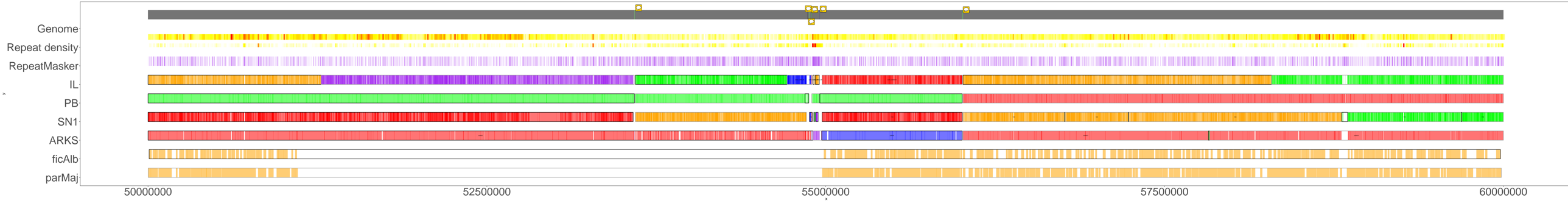

Supplement: Supplementary file 3 — Figure S8 [file MEN-21-263-s003.zip › PGA_scaffold1_plot_6.pdf]

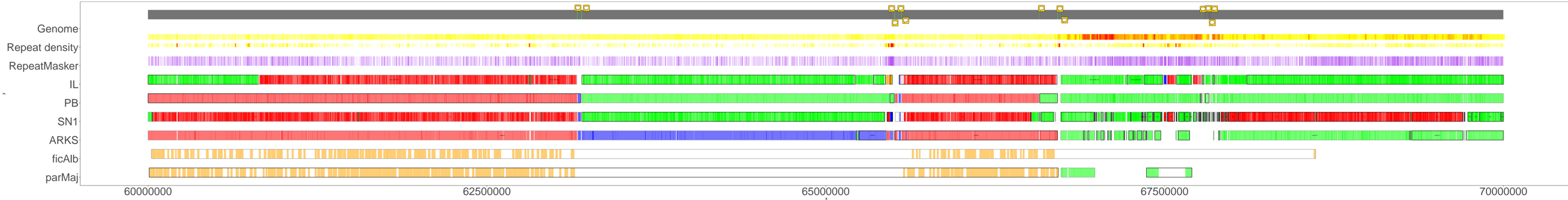

Supplement: Supplementary file 3 — Figure S8 [file MEN-21-263-s003.zip › PGA_scaffold1_plot_7.pdf]

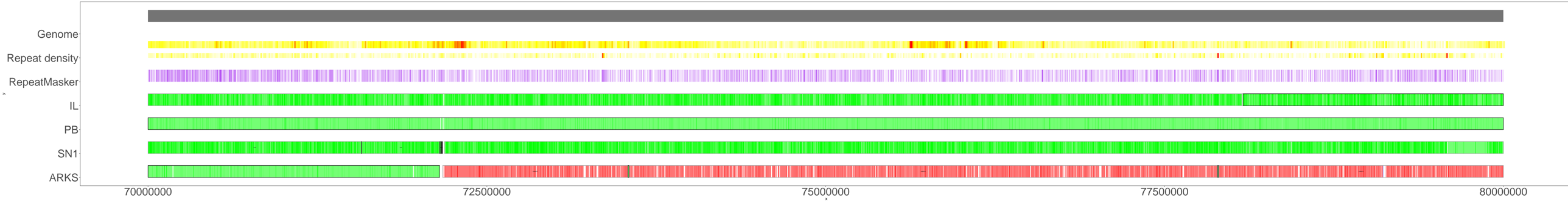

Supplement: Supplementary file 3 — Figure S8 [file MEN-21-263-s003.zip › PGA_scaffold1_plot_8.pdf]

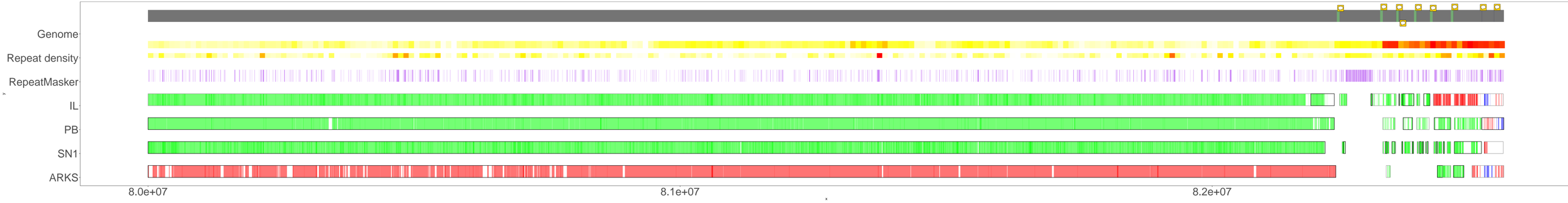

Supplement: Supplementary file 3 — Figure S8 [file MEN-21-263-s003.zip › PGA_scaffold1_plot_9.pdf]

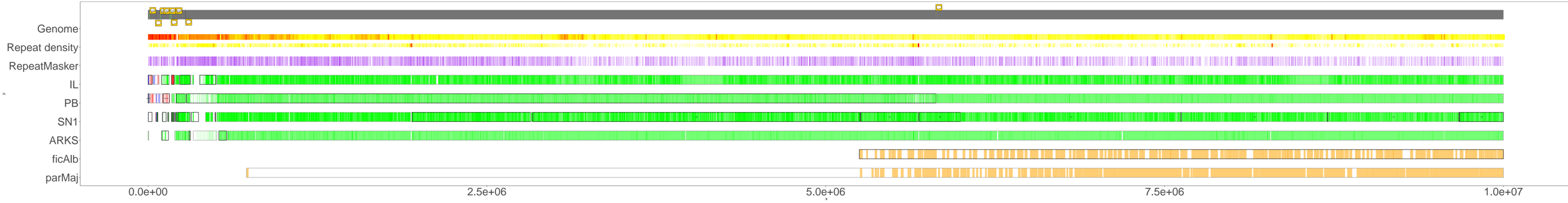

Supplement: Supplementary file 3 — Figure S8 [file MEN-21-263-s003.zip › PGA_scaffold2_plot_1.pdf]

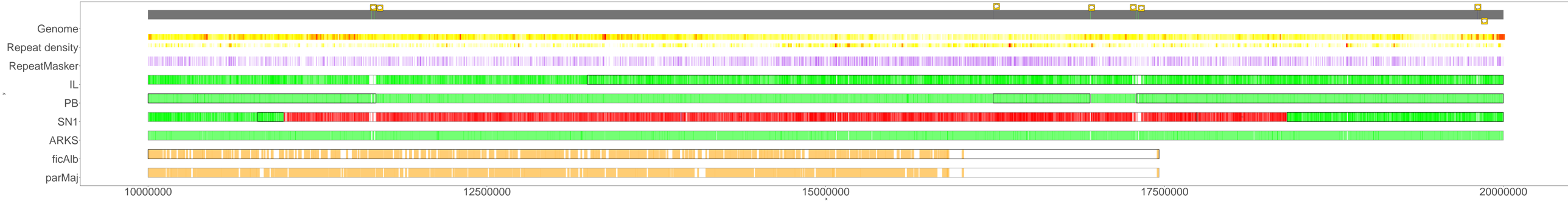

Supplement: Supplementary file 3 — Figure S8 [file MEN-21-263-s003.zip › PGA_scaffold2_plot_2.pdf]

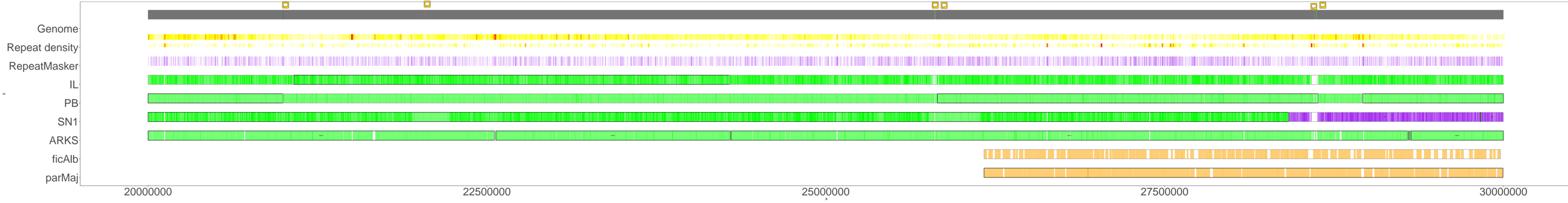

Supplement: Supplementary file 3 — Figure S8 [file MEN-21-263-s003.zip › PGA_scaffold2_plot_3.pdf]

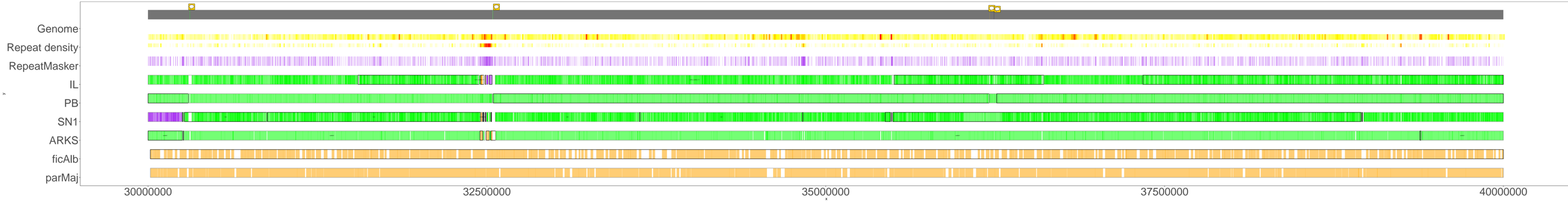

Supplement: Supplementary file 3 — Figure S8 [file MEN-21-263-s003.zip › PGA_scaffold2_plot_4.pdf]

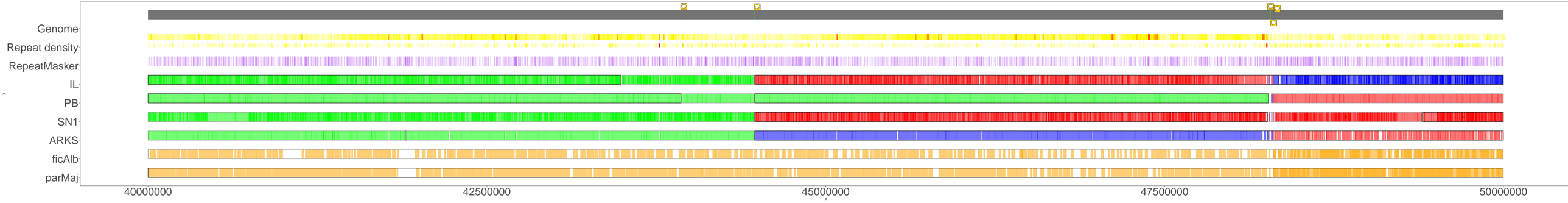

Supplement: Supplementary file 3 — Figure S8 [file MEN-21-263-s003.zip › PGA_scaffold2_plot_5.pdf]

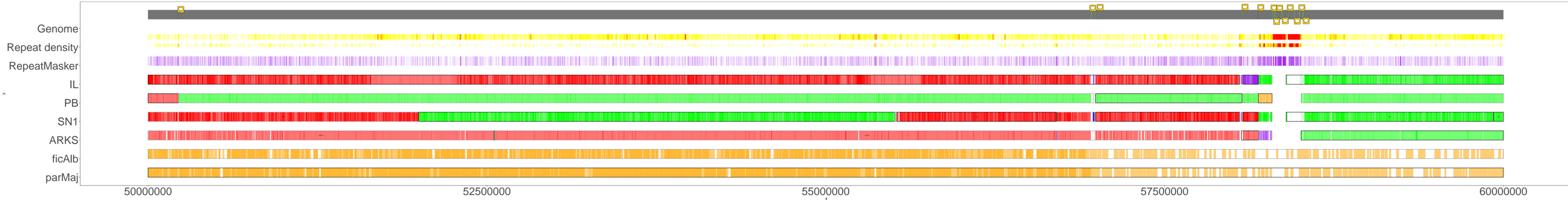

Supplement: Supplementary file 3 — Figure S8 [file MEN-21-263-s003.zip › PGA_scaffold2_plot_6.pdf]

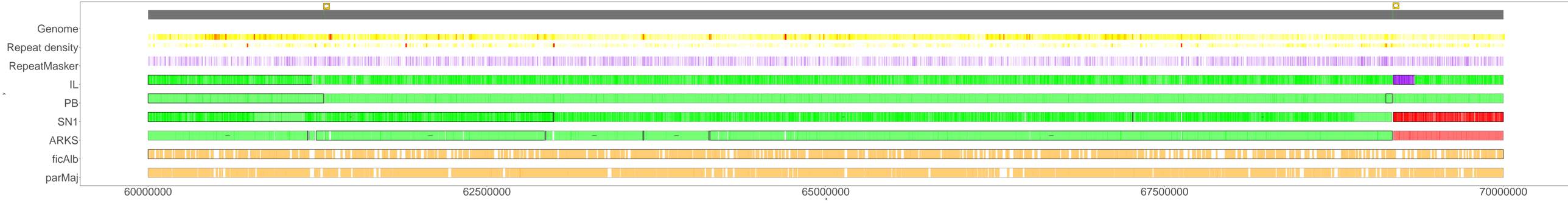

Supplement: Supplementary file 3 — Figure S8 [file MEN-21-263-s003.zip › PGA_scaffold2_plot_7.pdf]

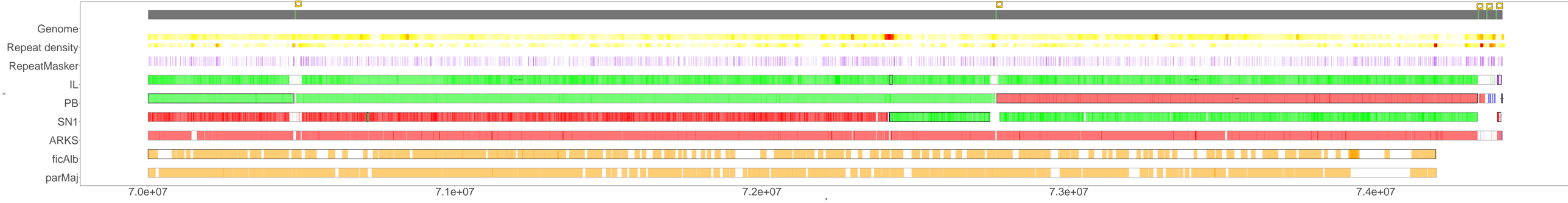

Supplement: Supplementary file 3 — Figure S8 [file MEN-21-263-s003.zip › PGA_scaffold2_plot_8.pdf]

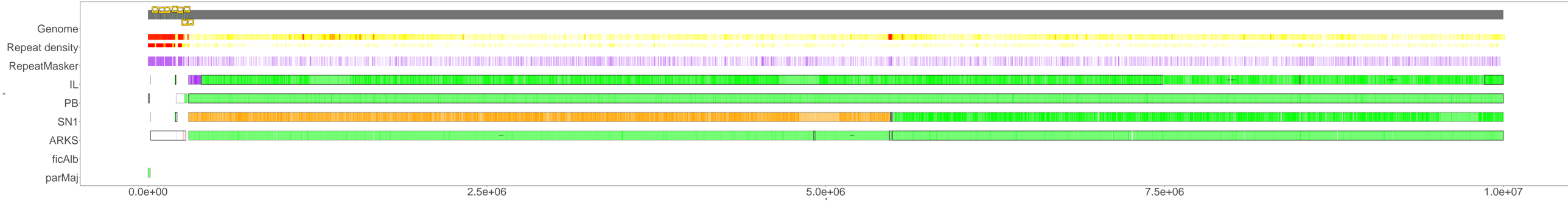

Supplement: Supplementary file 3 — Figure S8 [file MEN-21-263-s003.zip › PGA_scaffold3_plot_1.pdf]

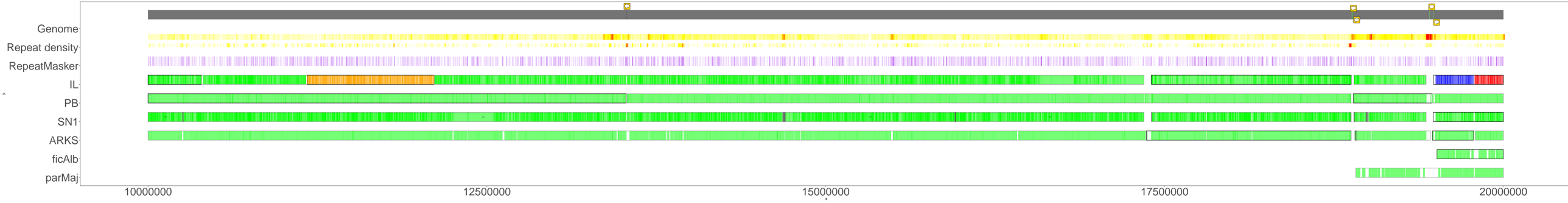

Supplement: Supplementary file 3 — Figure S8 [file MEN-21-263-s003.zip › PGA_scaffold3_plot_2.pdf]

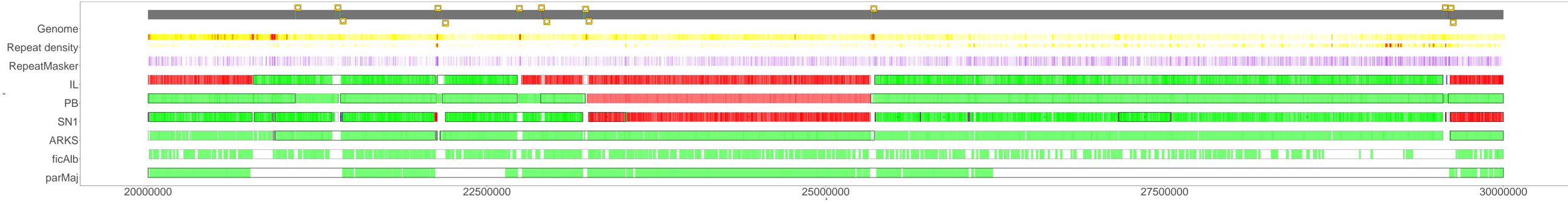

Supplement: Supplementary file 3 — Figure S8 [file MEN-21-263-s003.zip › PGA_scaffold3_plot_3.pdf]

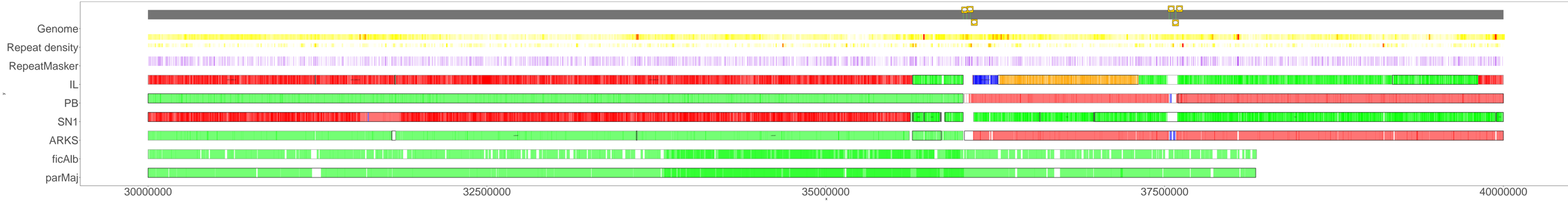

Supplement: Supplementary file 3 — Figure S8 [file MEN-21-263-s003.zip › PGA_scaffold3_plot_4.pdf]

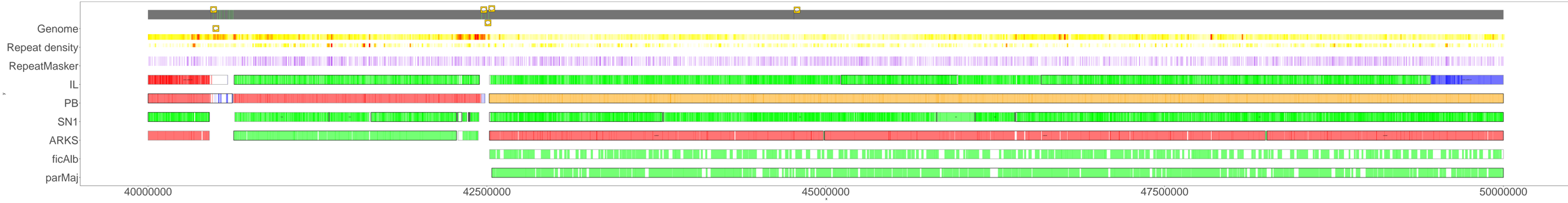

Supplement: Supplementary file 3 — Figure S8 [file MEN-21-263-s003.zip › PGA_scaffold3_plot_5.pdf]

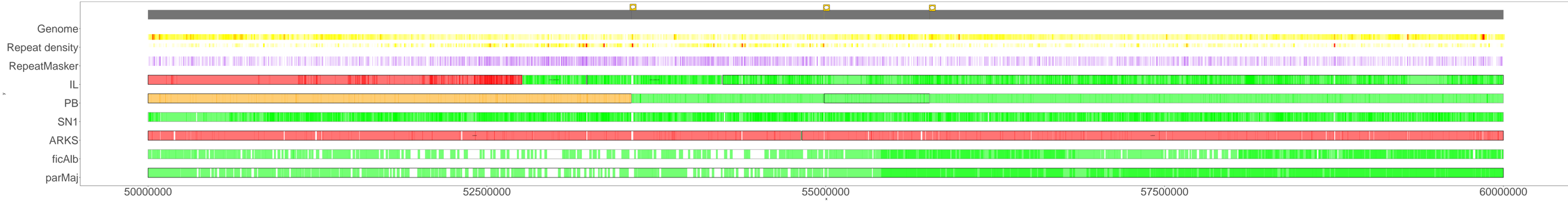

Supplement: Supplementary file 3 — Figure S8 [file MEN-21-263-s003.zip › PGA_scaffold3_plot_6.pdf]

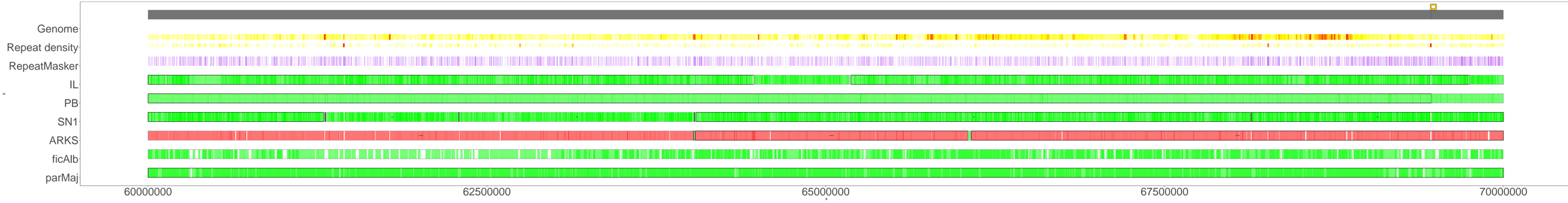

Supplement: Supplementary file 3 — Figure S8 [file MEN-21-263-s003.zip › PGA_scaffold3_plot_7.pdf]

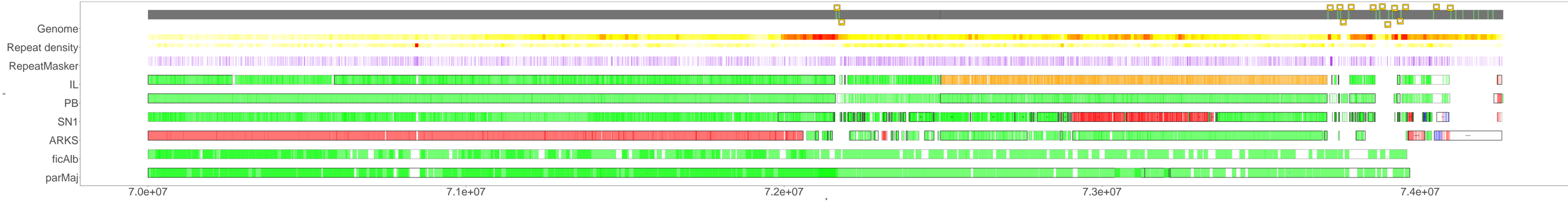

Supplement: Supplementary file 3 — Figure S8 [file MEN-21-263-s003.zip › PGA_scaffold3_plot_8.pdf]

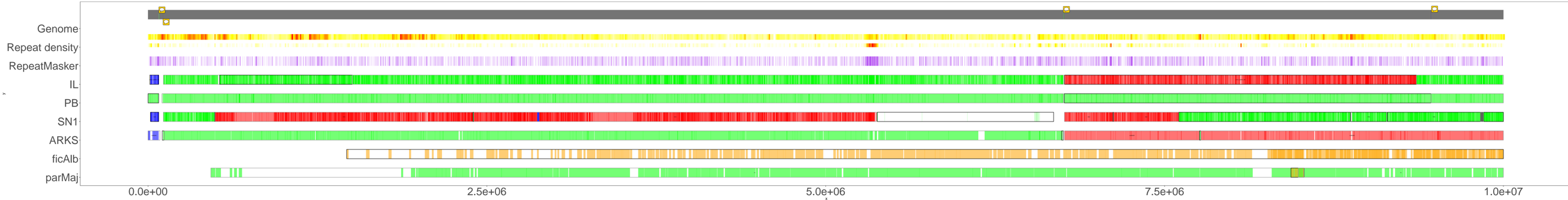

Supplement: Supplementary file 3 — Figure S8 [file MEN-21-263-s003.zip › PGA_scaffold4_plot_1.pdf]

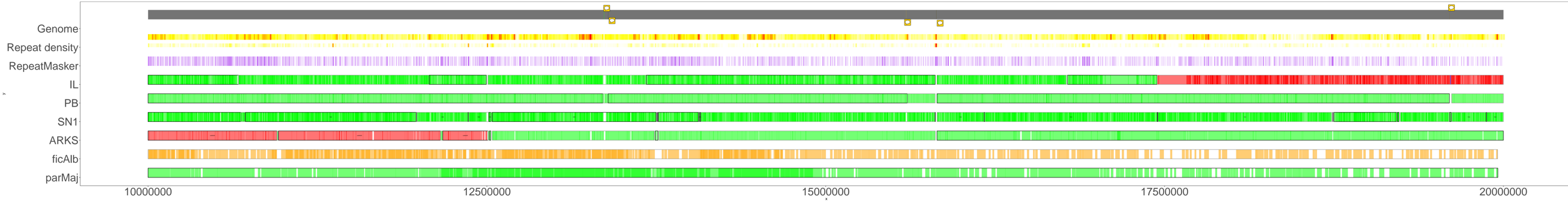

Supplement: Supplementary file 3 — Figure S8 [file MEN-21-263-s003.zip › PGA_scaffold4_plot_2.pdf]

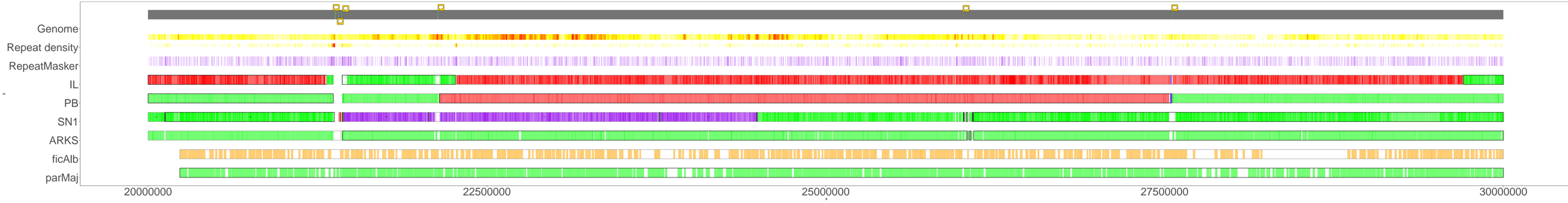

Supplement: Supplementary file 3 — Figure S8 [file MEN-21-263-s003.zip › PGA_scaffold4_plot_3.pdf]

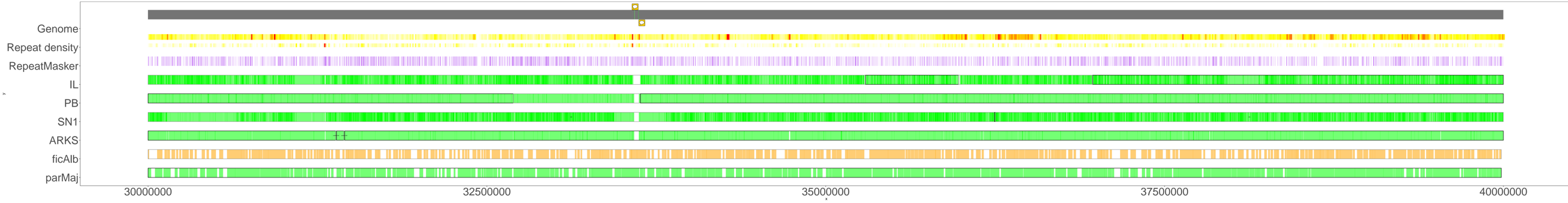

Supplement: Supplementary file 3 — Figure S8 [file MEN-21-263-s003.zip › PGA_scaffold4_plot_4.pdf]

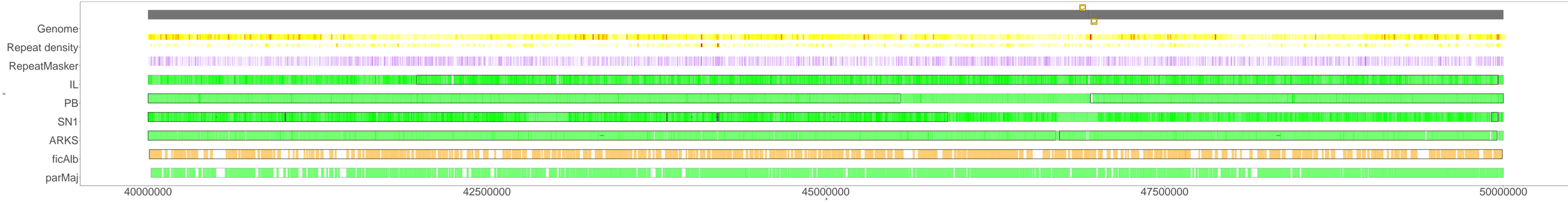

Supplement: Supplementary file 3 — Figure S8 [file MEN-21-263-s003.zip › PGA_scaffold4_plot_5.pdf]

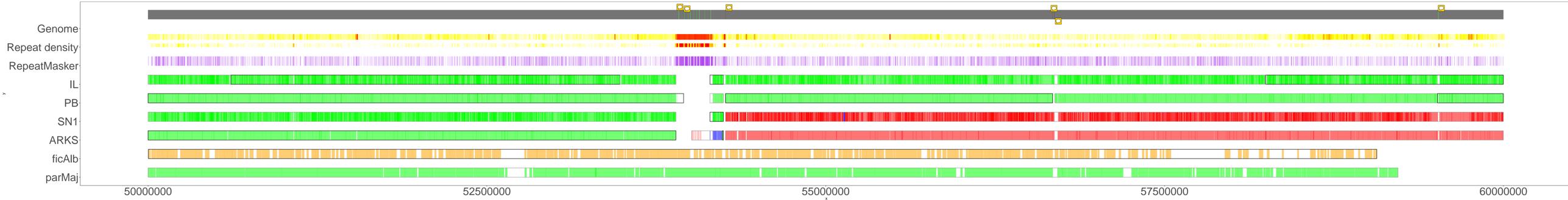

Supplement: Supplementary file 3 — Figure S8 [file MEN-21-263-s003.zip › PGA_scaffold4_plot_6.pdf]

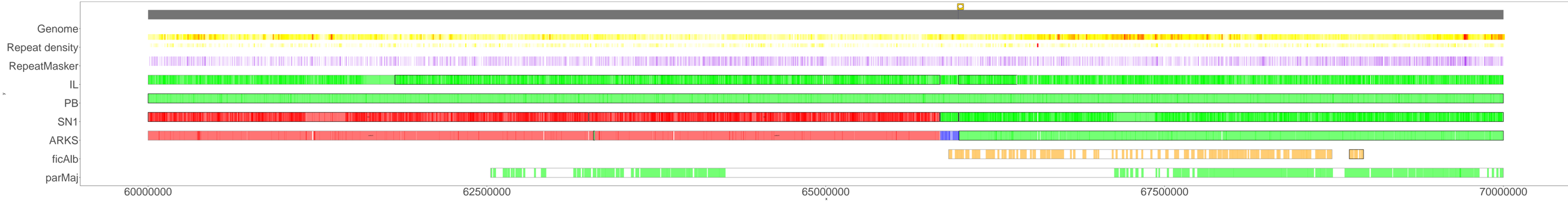

Supplement: Supplementary file 3 — Figure S8 [file MEN-21-263-s003.zip › PGA_scaffold4_plot_7.pdf]

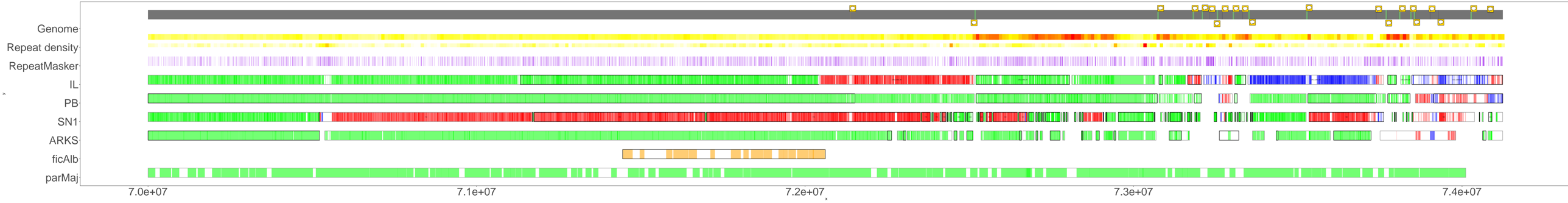

Supplement: Supplementary file 3 — Figure S8 [file MEN-21-263-s003.zip › PGA_scaffold4_plot_8.pdf]

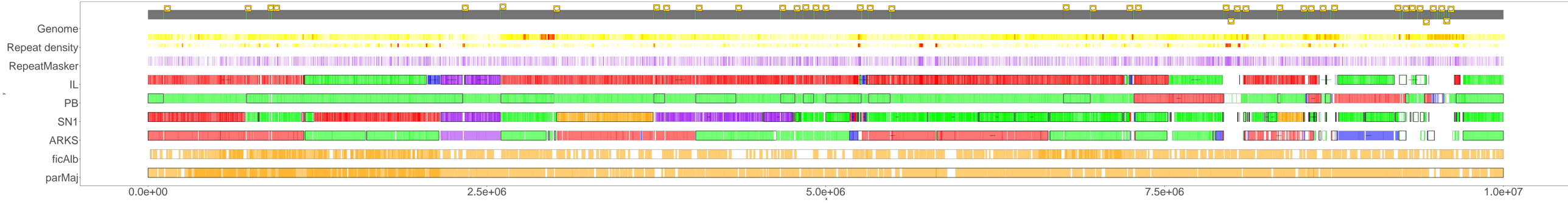

Supplement: Supplementary file 3 — Figure S8 [file MEN-21-263-s003.zip › PGA_scaffold5_plot_1.pdf]

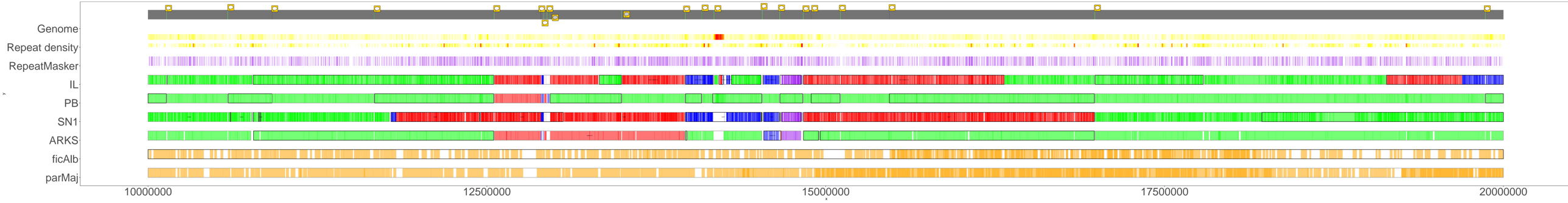

Supplement: Supplementary file 3 — Figure S8 [file MEN-21-263-s003.zip › PGA_scaffold5_plot_2.pdf]

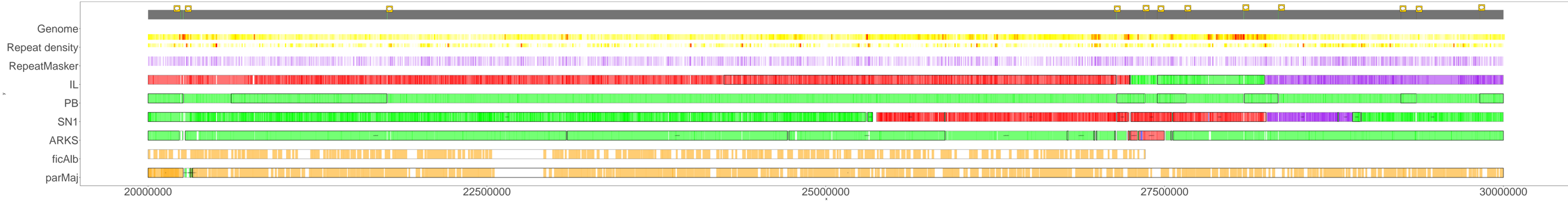

Supplement: Supplementary file 3 — Figure S8 [file MEN-21-263-s003.zip › PGA_scaffold5_plot_3.pdf]

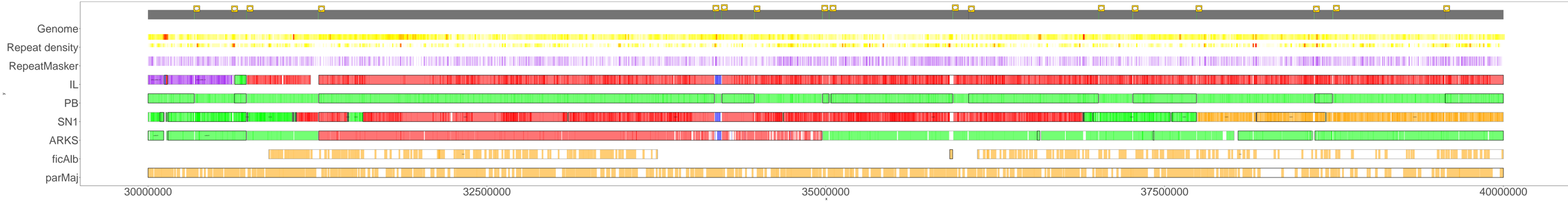

Supplement: Supplementary file 3 — Figure S8 [file MEN-21-263-s003.zip › PGA_scaffold5_plot_4.pdf]

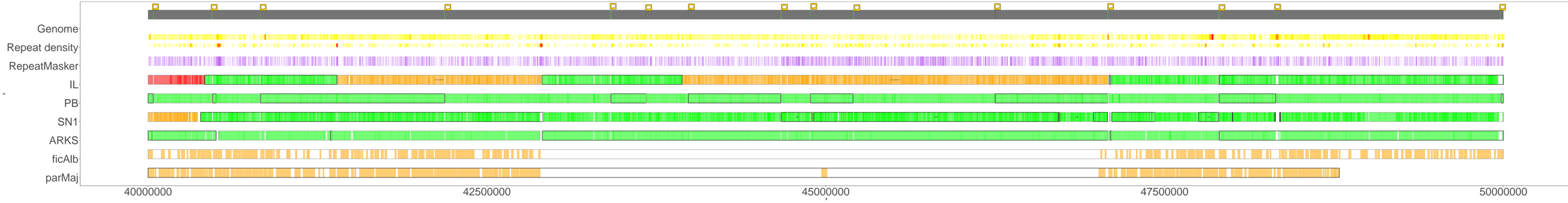

Supplement: Supplementary file 3 — Figure S8 [file MEN-21-263-s003.zip › PGA_scaffold5_plot_5.pdf]

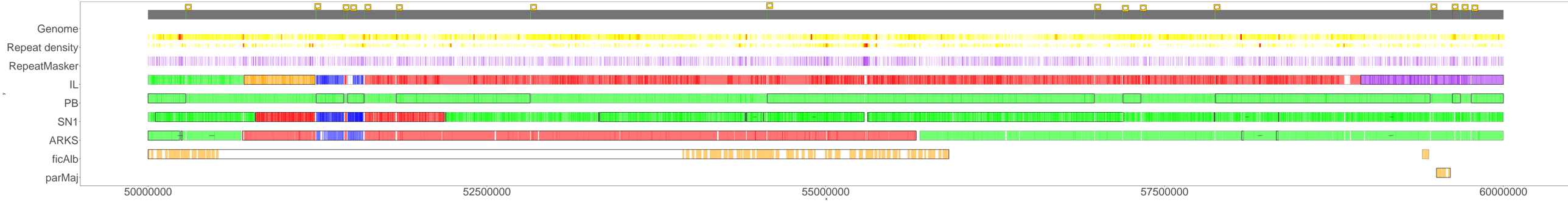

Supplement: Supplementary file 3 — Figure S8 [file MEN-21-263-s003.zip › PGA_scaffold5_plot_6.pdf]

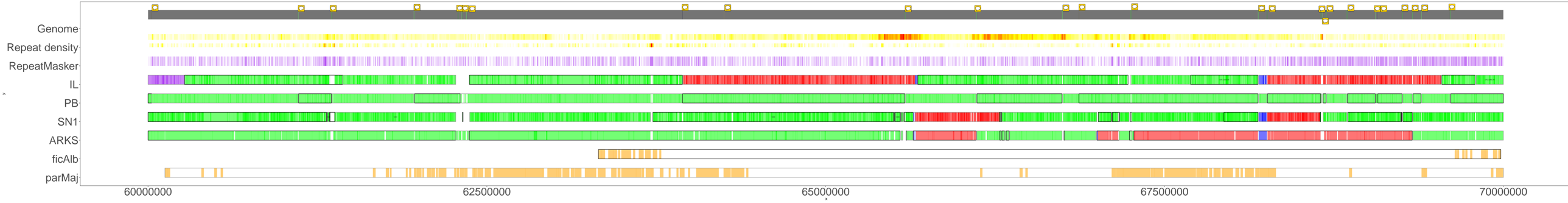

Supplement: Supplementary file 3 — Figure S8 [file MEN-21-263-s003.zip › PGA_scaffold5_plot_7.pdf]

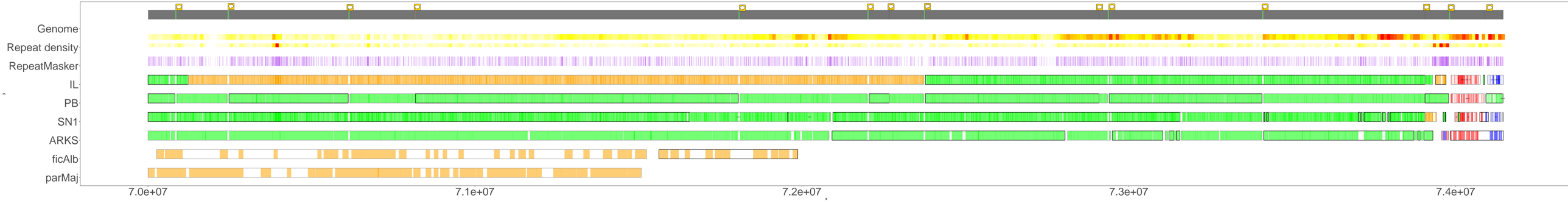

Supplement: Supplementary file 3 — Figure S8 [file MEN-21-263-s003.zip › PGA_scaffold5_plot_8.pdf]

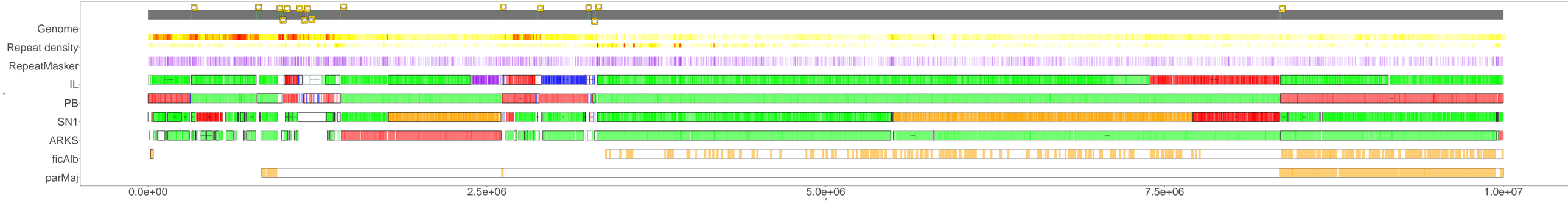

Supplement: Supplementary file 3 — Figure S8 [file MEN-21-263-s003.zip › PGA_scaffold6_plot_1.pdf]

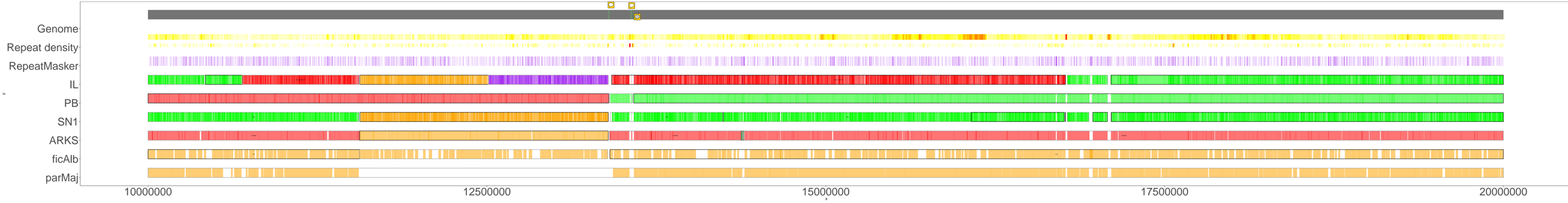

Supplement: Supplementary file 3 — Figure S8 [file MEN-21-263-s003.zip › PGA_scaffold6_plot_2.pdf]

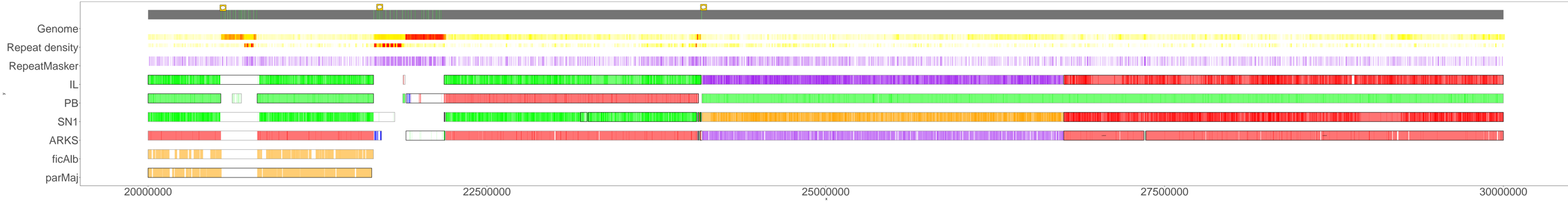

Supplement: Supplementary file 3 — Figure S8 [file MEN-21-263-s003.zip › PGA_scaffold6_plot_3.pdf]

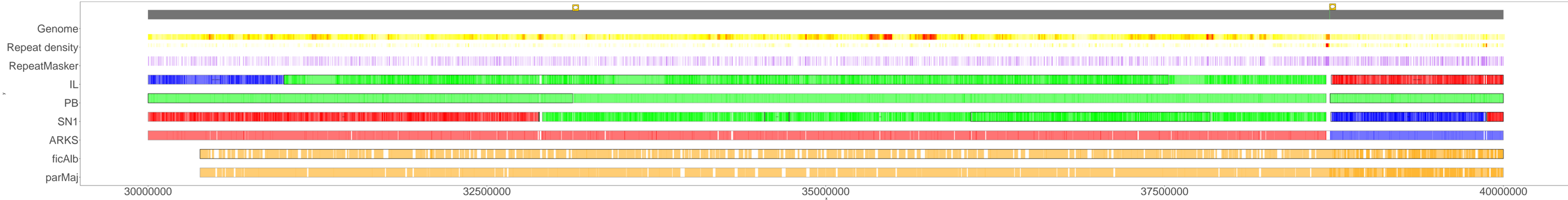

Supplement: Supplementary file 3 — Figure S8 [file MEN-21-263-s003.zip › PGA_scaffold6_plot_4.pdf]

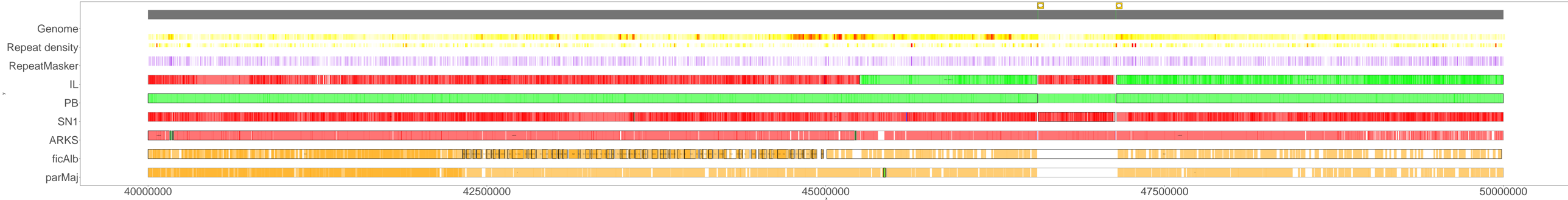

Supplement: Supplementary file 3 — Figure S8 [file MEN-21-263-s003.zip › PGA_scaffold6_plot_5.pdf]

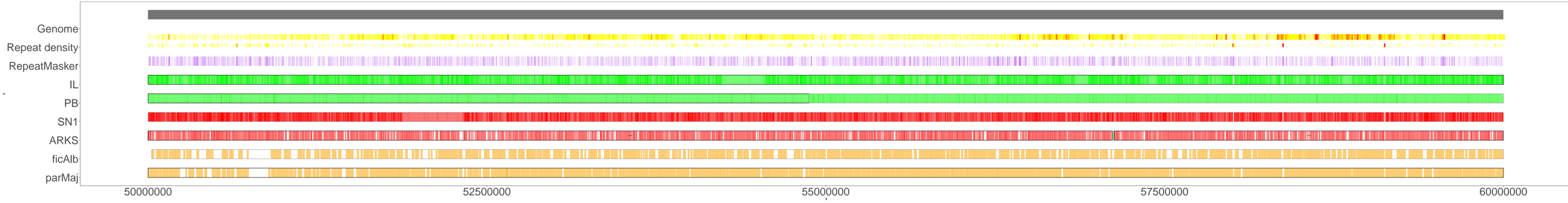

Supplement: Supplementary file 3 — Figure S8 [file MEN-21-263-s003.zip › PGA_scaffold6_plot_6.pdf]

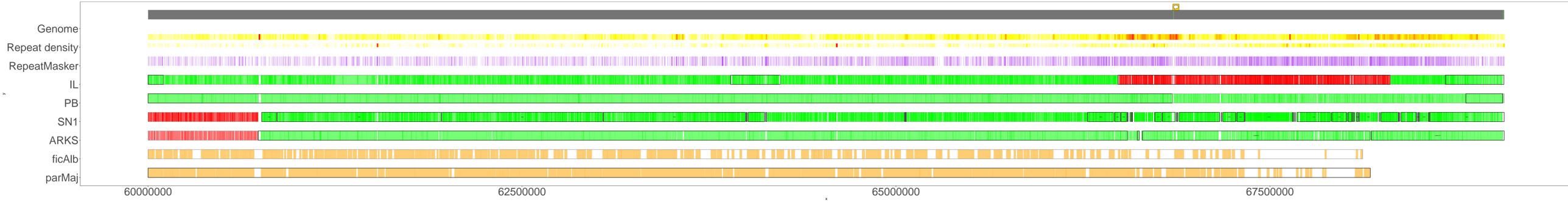

Supplement: Supplementary file 3 — Figure S8 [file MEN-21-263-s003.zip › PGA_scaffold6_plot_7.pdf]

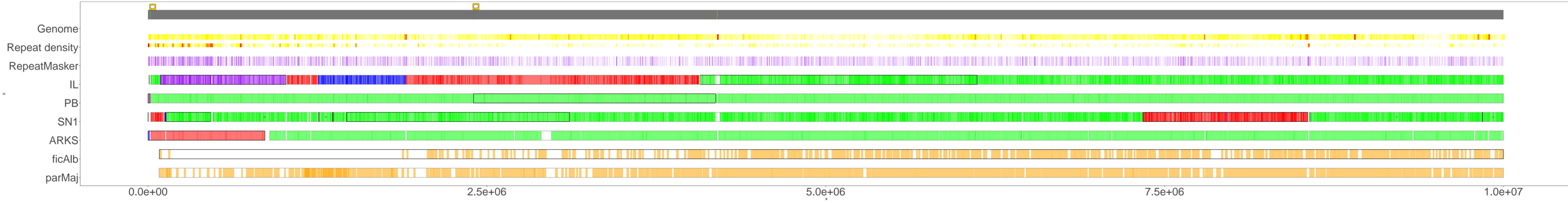

Supplement: Supplementary file 3 — Figure S8 [file MEN-21-263-s003.zip › PGA_scaffold7_plot_1.pdf]

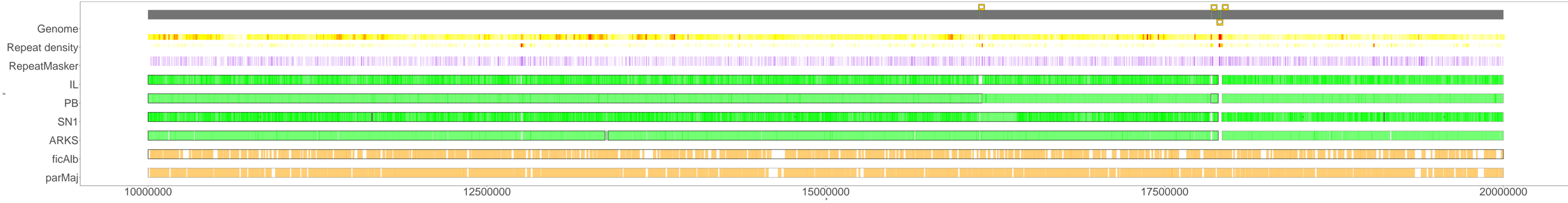

Supplement: Supplementary file 3 — Figure S8 [file MEN-21-263-s003.zip › PGA_scaffold7_plot_2.pdf]

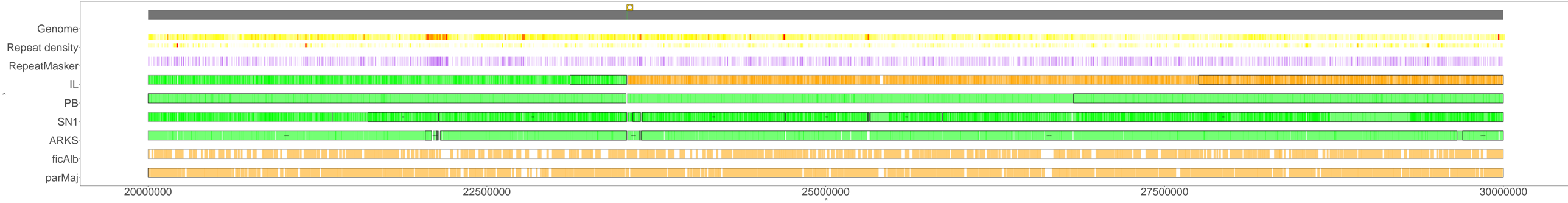

Supplement: Supplementary file 3 — Figure S8 [file MEN-21-263-s003.zip › PGA_scaffold7_plot_3.pdf]

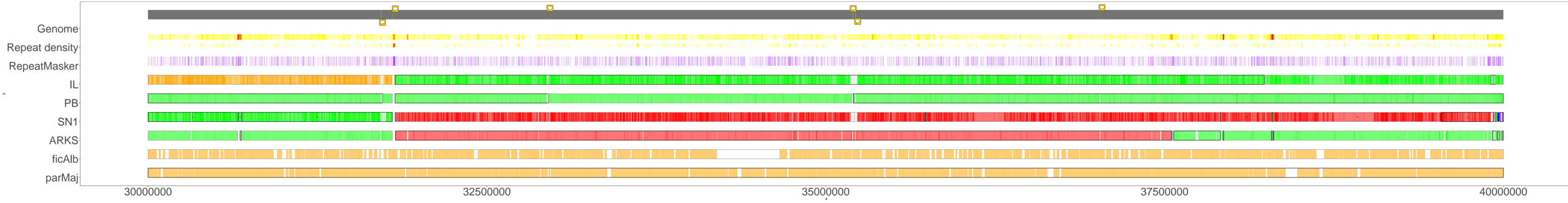

Supplement: Supplementary file 3 — Figure S8 [file MEN-21-263-s003.zip › PGA_scaffold7_plot_4.pdf]

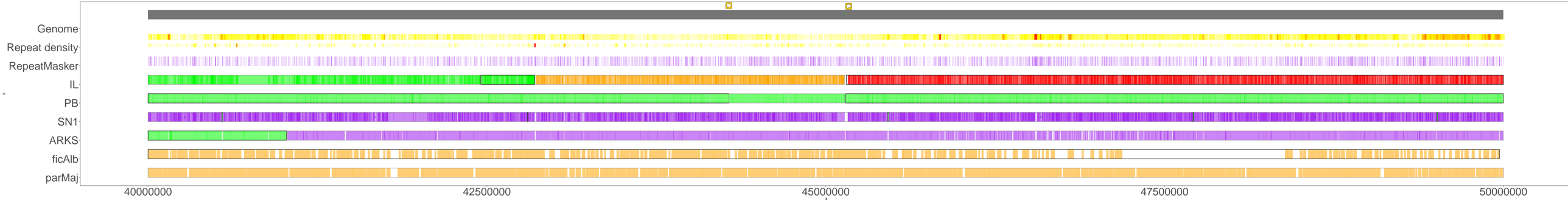

Supplement: Supplementary file 3 — Figure S8 [file MEN-21-263-s003.zip › PGA_scaffold7_plot_5.pdf]

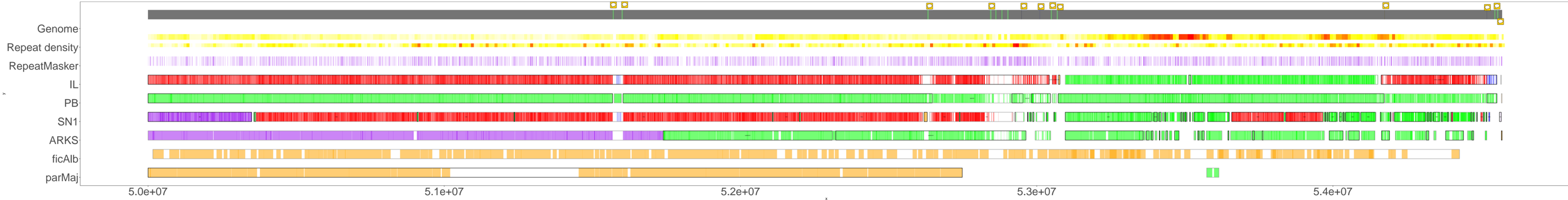

Supplement: Supplementary file 3 — Figure S8 [file MEN-21-263-s003.zip › PGA_scaffold7_plot_6.pdf]

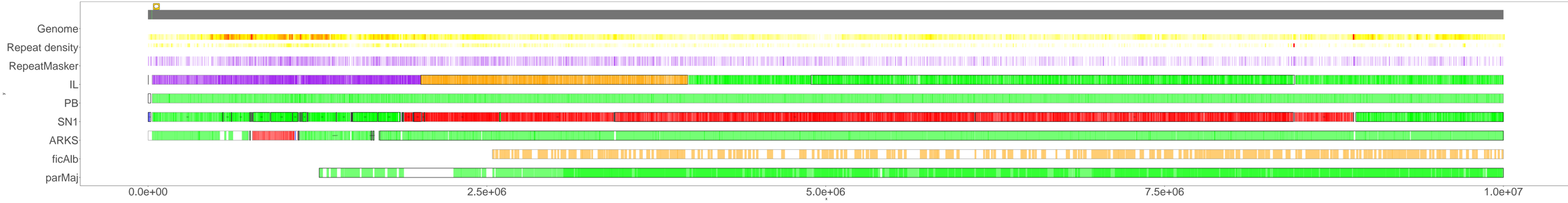

Supplement: Supplementary file 3 — Figure S8 [file MEN-21-263-s003.zip › PGA_scaffold8_plot_1.pdf]

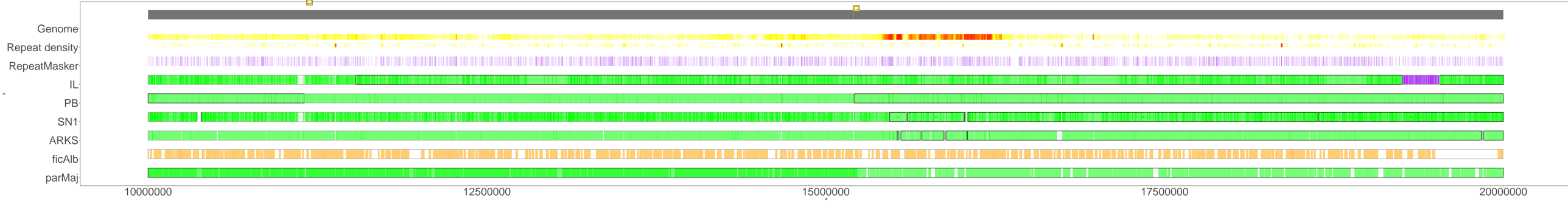

Supplement: Supplementary file 3 — Figure S8 [file MEN-21-263-s003.zip › PGA_scaffold8_plot_2.pdf]

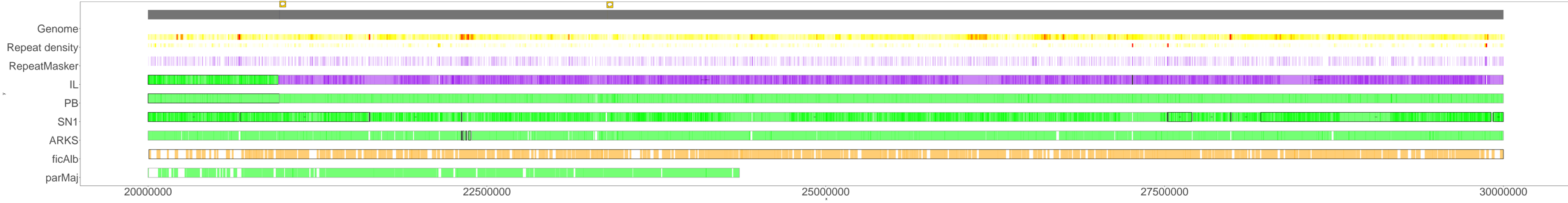

Supplement: Supplementary file 3 — Figure S8 [file MEN-21-263-s003.zip › PGA_scaffold8_plot_3.pdf]

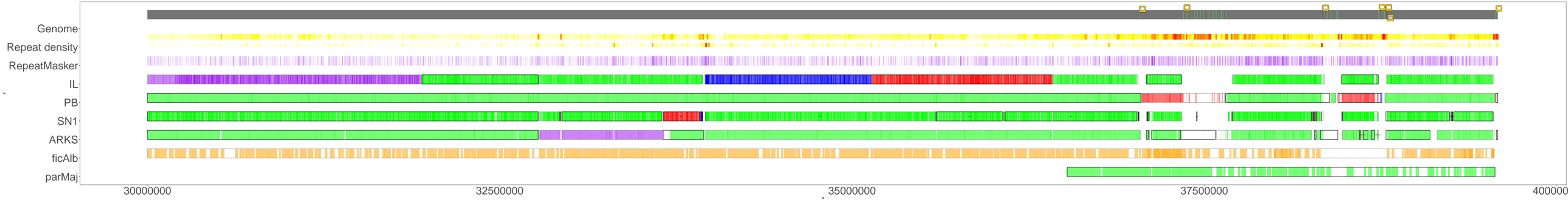

Supplement: Supplementary file 3 — Figure S8 [file MEN-21-263-s003.zip › PGA_scaffold8_plot_4.pdf]

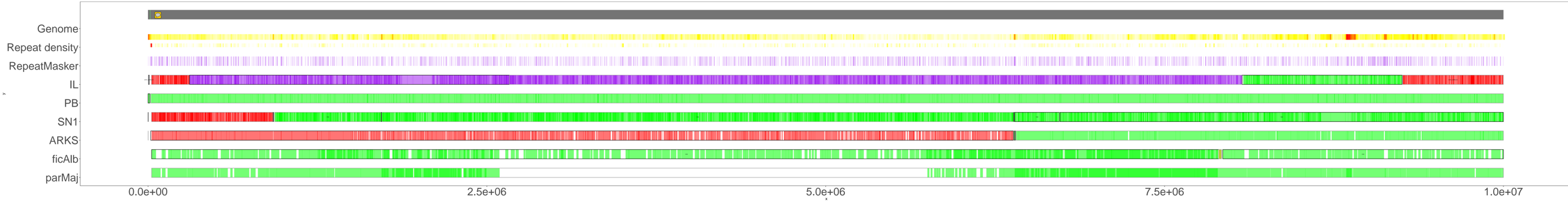

Supplement: Supplementary file 3 — Figure S8 [file MEN-21-263-s003.zip › PGA_scaffold9_plot_1.pdf]

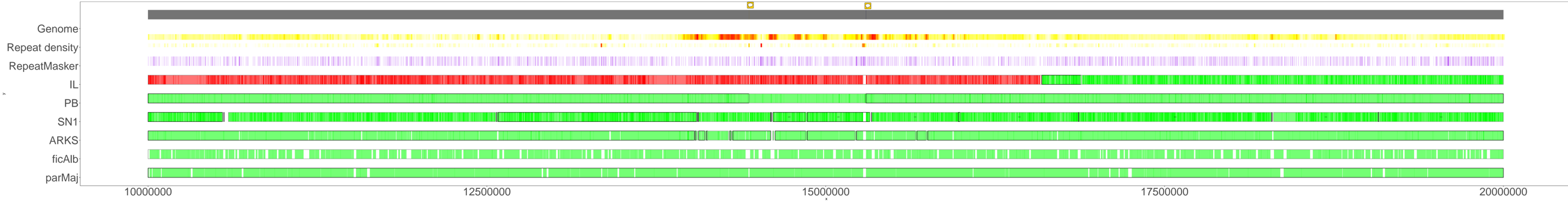

Supplement: Supplementary file 3 — Figure S8 [file MEN-21-263-s003.zip › PGA_scaffold9_plot_2.pdf]

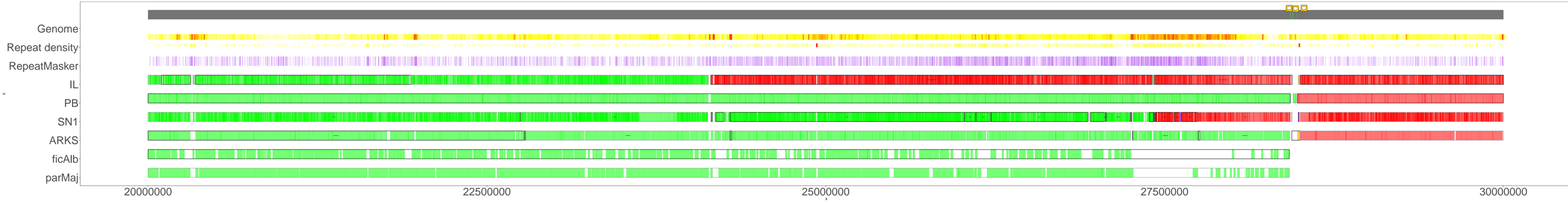

Supplement: Supplementary file 3 — Figure S8 [file MEN-21-263-s003.zip › PGA_scaffold9_plot_3.pdf]

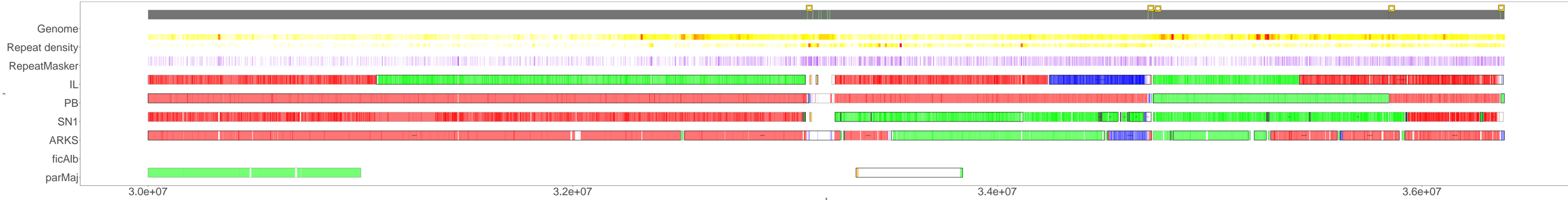

Supplement: Supplementary file 3 — Figure S8 [file MEN-21-263-s003.zip › PGA_scaffold9_plot_4.pdf]

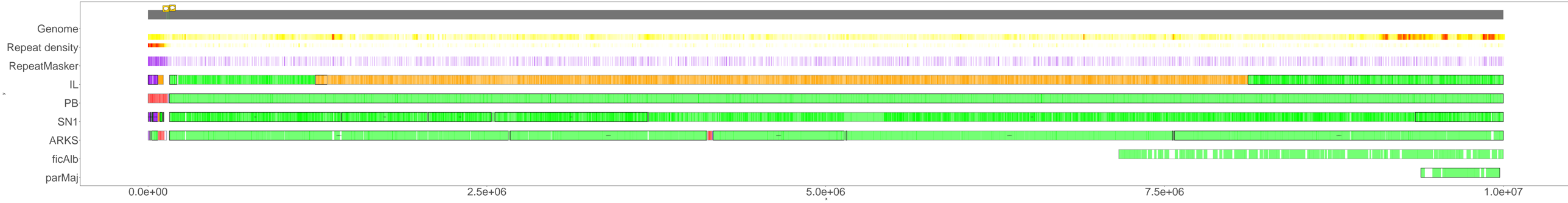

Supplement: Supplementary file 3 — Figure S8 [file MEN-21-263-s003.zip › PGA_scaffold10_plot_1.pdf]

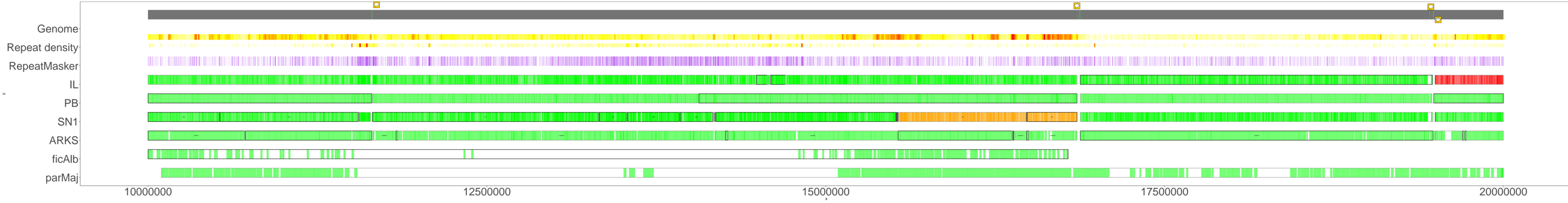

Supplement: Supplementary file 3 — Figure S8 [file MEN-21-263-s003.zip › PGA_scaffold10_plot_2.pdf]

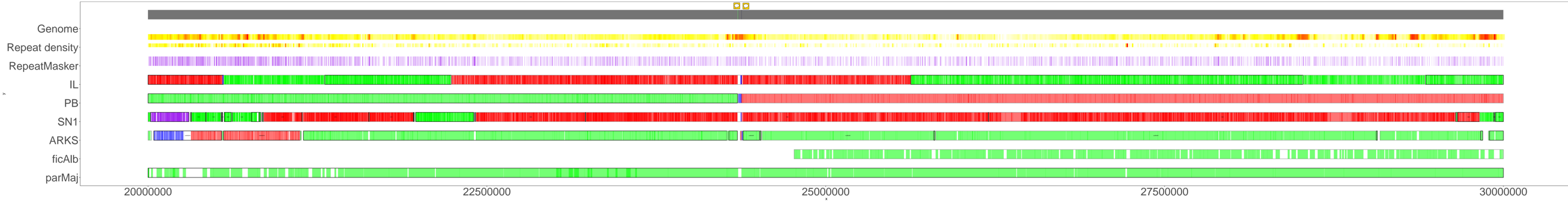

Supplement: Supplementary file 3 — Figure S8 [file MEN-21-263-s003.zip › PGA_scaffold10_plot_3.pdf]

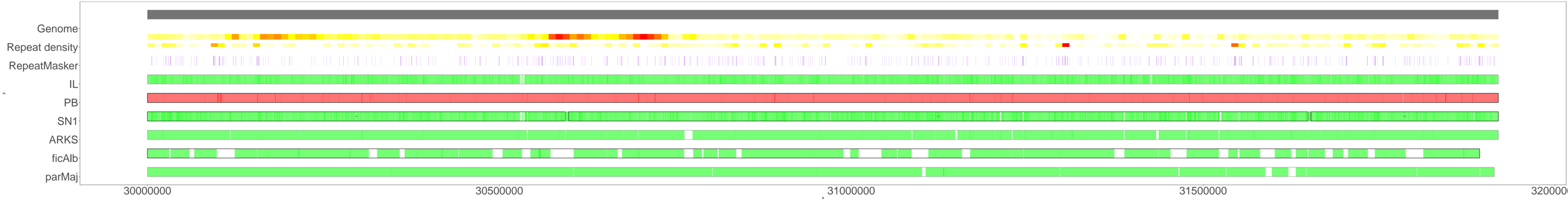

Supplement: Supplementary file 3 — Figure S8 [file MEN-21-263-s003.zip › PGA_scaffold10_plot_4.pdf]

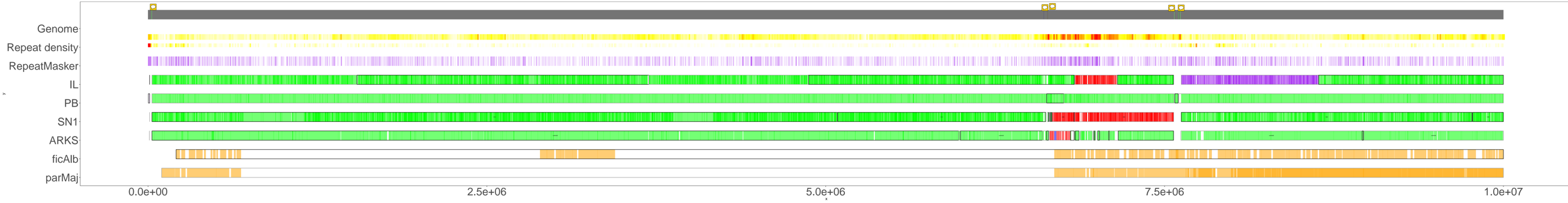

Supplement: Supplementary file 3 — Figure S8 [file MEN-21-263-s003.zip › PGA_scaffold11_plot_1.pdf]

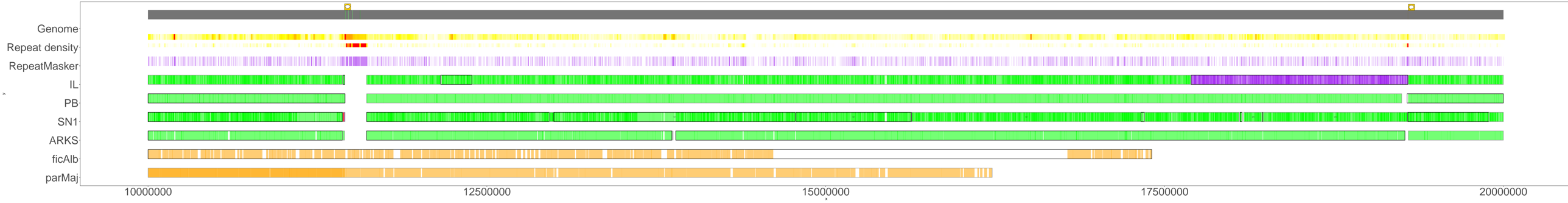

Supplement: Supplementary file 3 — Figure S8 [file MEN-21-263-s003.zip › PGA_scaffold11_plot_2.pdf]

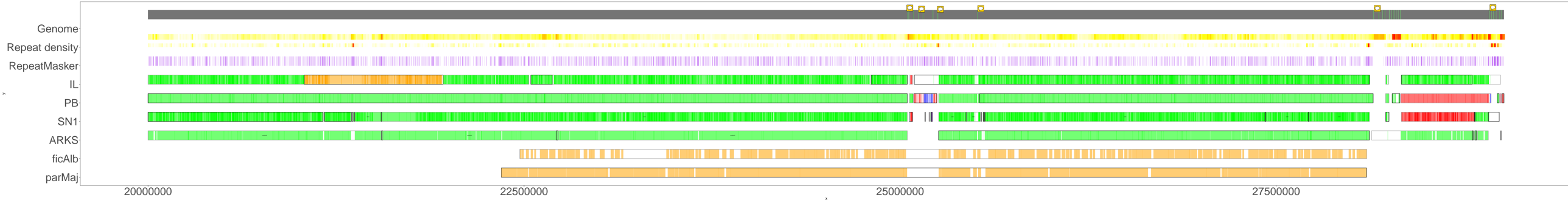

Supplement: Supplementary file 3 — Figure S8 [file MEN-21-263-s003.zip › PGA_scaffold11_plot_3.pdf]

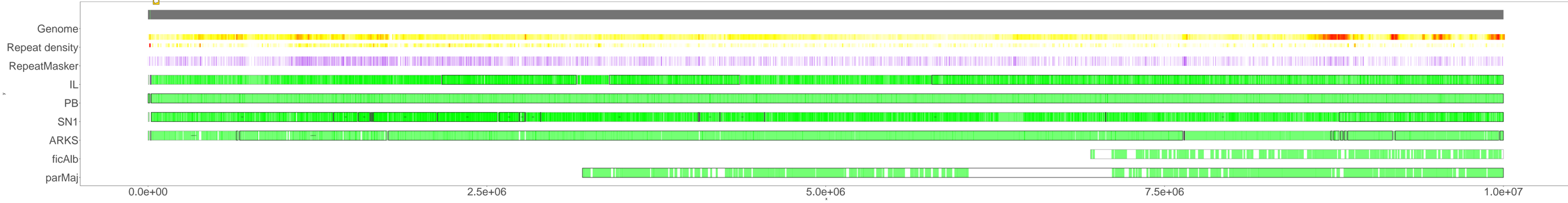

Supplement: Supplementary file 3 — Figure S8 [file MEN-21-263-s003.zip › PGA_scaffold12_plot_1.pdf]

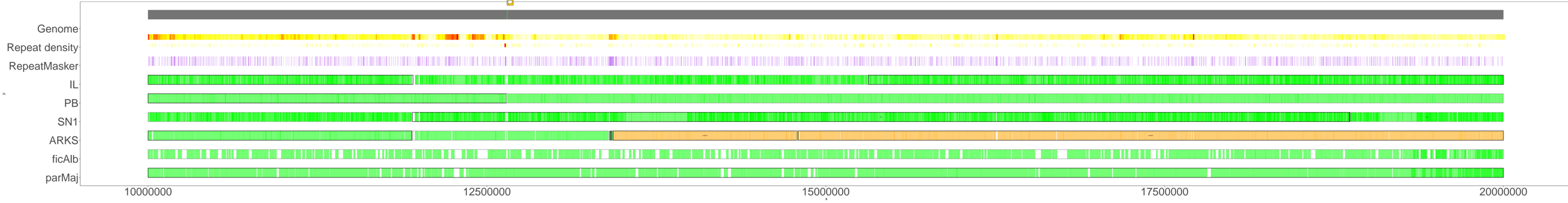

Supplement: Supplementary file 3 — Figure S8 [file MEN-21-263-s003.zip › PGA_scaffold12_plot_2.pdf]

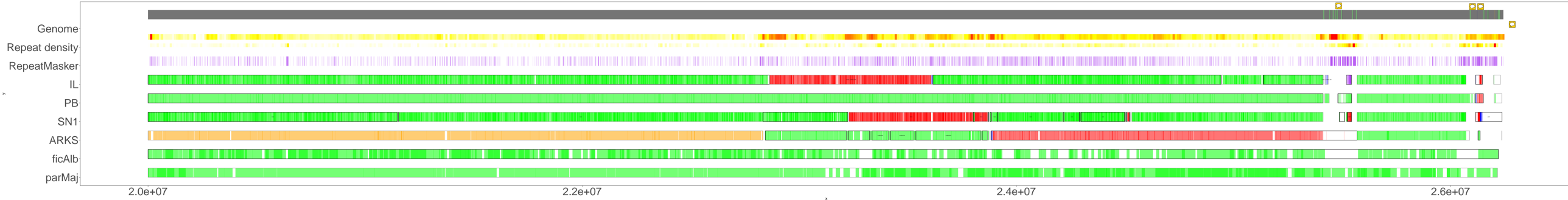

Supplement: Supplementary file 3 — Figure S8 [file MEN-21-263-s003.zip › PGA_scaffold12_plot_3.pdf]

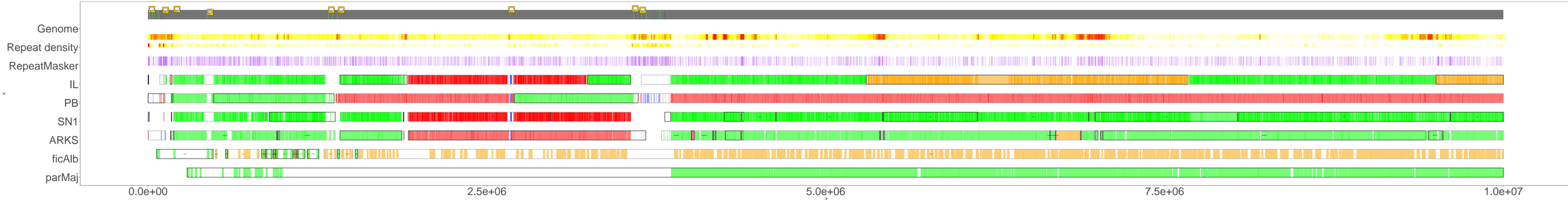

Supplement: Supplementary file 3 — Figure S8 [file MEN-21-263-s003.zip › PGA_scaffold13_plot_1.pdf]

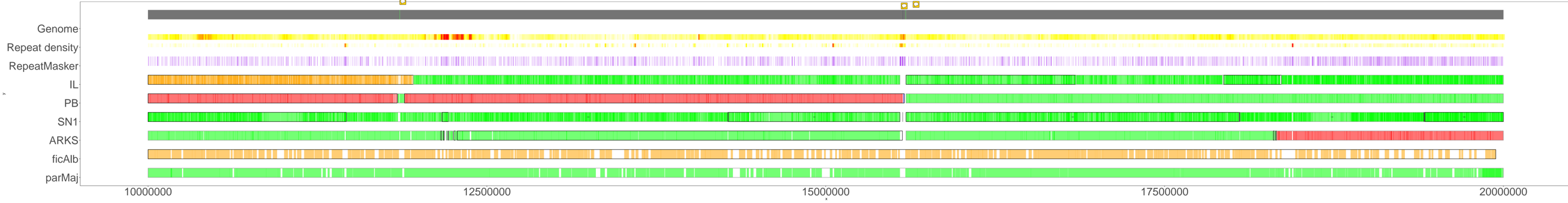

Supplement: Supplementary file 3 — Figure S8 [file MEN-21-263-s003.zip › PGA_scaffold13_plot_2.pdf]

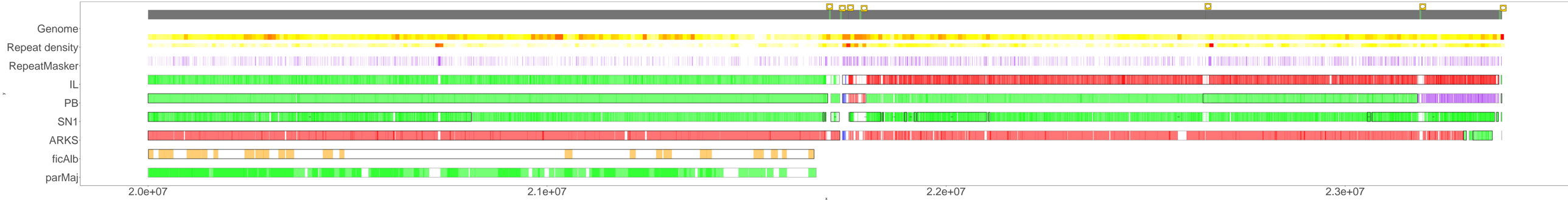

Supplement: Supplementary file 3 — Figure S8 [file MEN-21-263-s003.zip › PGA_scaffold13_plot_3.pdf]

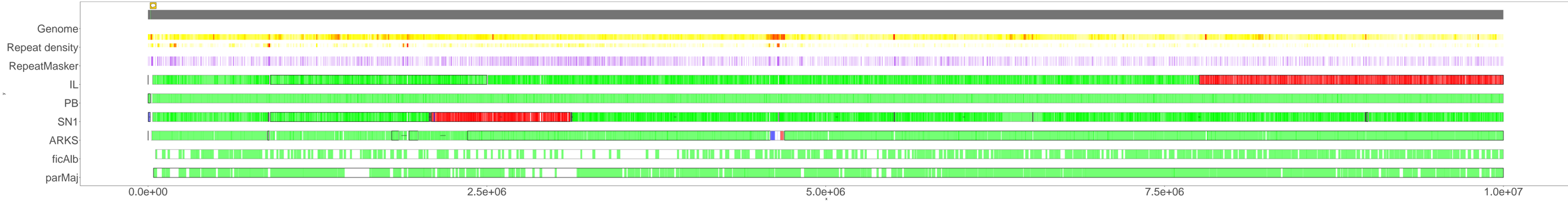

Supplement: Supplementary file 3 — Figure S8 [file MEN-21-263-s003.zip › PGA_scaffold14_plot_1.pdf]

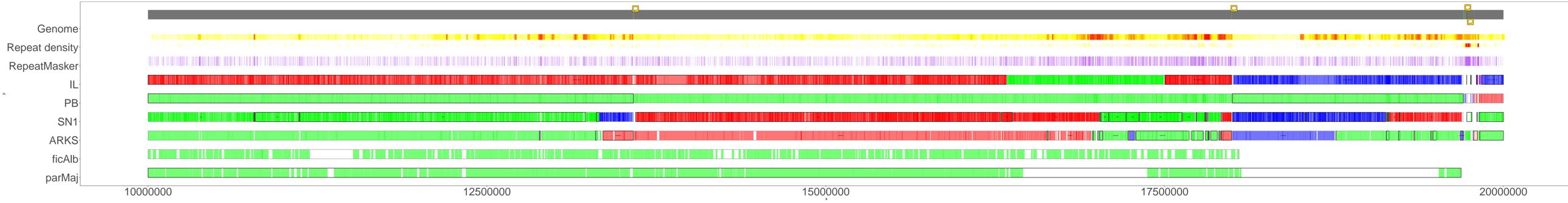

Supplement: Supplementary file 3 — Figure S8 [file MEN-21-263-s003.zip › PGA_scaffold14_plot_2.pdf]
