# Supplementary material for: Identifying the causes and consequences of assembly gaps using a multiplatform genome assembly of a bird‐of‐paradise
Source: Mol Ecol Resour. 2020 Oct 10;21(1):263–86. doi: 10.1111/1755-0998.13252 (PMC7757076; doi:10.1111/1755-0998.13252)
Supplement: Supplementary file 6 — Text S1 [file MEN-21-263-s006.pdf]

Identifying the causes and consequences of assembly gaps using a multiplatform genome assembly of a bird-of-paradise

Valentina Peona[1,2], Mozes P.K. Blom[3,4], Luohao Xu[5], Reto Burri[6], Shawn Sullivan[7], Ignas Bunikis[8], Ivan Liachko[7], Tri Haryoko[9], Knud A. Jønsson[10], Qi Zhou[5,11,12], Martin Irestedt[3], Alexander Suh[1,2,13]

Affiliation

[1] Department of Ecology and Genetics – Evolutionary Biology, Uppsala University, Science for Life Laboratories, Norbyvägen 18D, SE-752 36, Uppsala, Sweden
[2] Department of Organismal Biology – Systematic Biology, Uppsala University, Norbyvägen 18D, SE-752 36, Uppsala, Sweden
[3] Department of Bioinformatics and Genetics, Swedish Museum of Natural History, SE-104 05, Stockholm, Sweden
[4] Museum für Naturkunde, Leibniz Institut für Evolutions- und Biodiversitätsforschung, Berlin, Germany
[5] Department of Neurosciences and Developmental Biology, University of Vienna, Vienna, Austria
[6] Department of Population Ecology, Institute of Ecology and Evolution, Friedrich-Schiller-University Jena, Dornburger Strasse 159, D-07743 Jena, Germany
[7] Phase Genomics, Inc. 1617 8th Ave N, Seattle, WA 98109 USA
[8] Uppsala Genome Center, Science for Life Laboratory, Dept. of Immunology, Genetics and Pathology, Uppsala University, SE‑752 37, Uppsala, Sweden
[9] Museum Zoologicum Bogoriense, Research Centre for Biology, Indonesian Institute of Sciences (LIPI), Cibinong, Indonesia
[10] Natural History Museum of Denmark, University of Copenhagen, Universitetsparken 15, DK-2100 Copenhagen, Denmark
[11] MOE Laboratory of Biosystems Homeostasis & Protection, Life Sciences Institute, Zhejiang University, Hangzhou, China
[12] Center for Reproductive Medicine, The 2nd Affiliated Hospital, School of Medicine, Zhejiang University
[13] School of Biological Sciences – Organisms and the Environment, University of East Anglia, NR4 7TJ, Norwich, UK

Supplementary Text

Multiplatform assembly

The first step was to assemble the PacBio reads into a primary assembly with the Falcon software (Chin et al. (2016); **Figure 1a**). The primary contigs were corrected and scaffolded with the Dovetail CHiCAGO map generating lycPyr2 (**Figure 1b**) using the software HiRise (Putnam et al., 2016). lycPyr2 then was polished with long reads (two runs of Arrow; Chin et al. (2016)) and short reads (three runs of Pilon 1.22; Walker et al. (2014); **Figure 1c**). Since PacBio sequencing is prone to introduce short indels in the reads (Eid et al., 2009), we addressed specifically these sequencing problems with Pilon while we did not correct single nucleotide variants in this step. Furthermore, in order to not over-polish repetitive regions (i.e., homogenising them with short reads), we excluded Pilon corrections falling within repeats identified by RepeatMasker 4.0.7 using our custom repeat library.

We then scaffolded lycPyr2 using the long-range information given by 10X Genomics linked reads with the software ARCS 1.0.1 (Yeo, Coombe, Warren, Chu, and Birol (2017); parameters -s 95 - e 1000 -m 20-100000) and LINKS 1.8.5 (Warren et al. (2015); parameters -a 0.2) generating lycPyr3 (**Figure 1d**). The parameters for ARCS and LINKS were chosen after generating 15 assemblies with different values for -m -e -a (**Supplementary Table S17**). The optimal parameter combination was established by minimising a) the number of "private" scaffolds belonging only to one combination of parameters and b) the number of scaffolds containing putatively erroneous chromosomal translocations.

LycPyr3 was scaffolded into chromosome models (clusters of contigs and scaffolds) with the Phase Genomics Hi-C data and the Proximo Hi-C scaffolding pipeline (lycPyr4; **Figure 1e**). Hi-C data were generated using a Phase Genomics (Seattle, WA) Proximo Hi-C Animal Kit. Following the manufacturer's instructions for the kit, intact cells from two samples were crosslinked using a formaldehyde solution, digested using the *Sau*3AI restriction enzyme, and proximity-ligated with biotinylated nucleotides to create chimeric molecules composed of fragments from different regions of the genome that were physically proximal *in-vivo*, but not necessarily linearly proximal. Continuing with the manufacturer's protocol, molecules were pulled down with streptavidin beads and processed into an Illumina-compatible sequencing library.

Reads were aligned to the draft assembly lycPyr3 following the manufacturer's recommendations. Briefly, reads were aligned using BWA-MEM (Li and Durbin (2010); v. 0.7.15-r1144-dirty) with the -5SP and -t 8 options specified, while keeping the other parameters as default. SAMBLASTER (Faust and Hall 2014) was used to flag PCR duplicates, which were later excluded from analysis. Alignments were then filtered with samtools (Li et al. (2009); v1.5, with htslib 1.5) using the -F 2304 filtering flag to remove non-primary and secondary alignments, as well as read pairs in which one or more mates were unmapped. Phase Genomics' Proximo Hi-C genome scaffolding platform was used to create chromosome-scale scaffolds from the draft assembly as described in Bickhart et al. (2017). As in the LACHESIS method (Burton et al., 2013), this process computes a contact frequency matrix from the aligned Hi-C read pairs, normalised by the number of *Sau*3AI restriction sites (GATC) on each contig, and constructs scaffolds in such a way as to optimise expected contact frequency and other statistical patterns in Hi-C data. Approximately 286,000 separate

Proximo runs were performed to optimise the number of scaffolds and scaffold construction in order to make the scaffolds as concordant with the observed Hi-C data as possible.

Two chromosomes (2 and 3) appeared to be split into two different super-scaffolds (or clusters) respectively, thus they were manually put together following the orientation suggested by the Hi- C interaction heatmap (**Figure S7**). We then manually inspected the assembly lycPyr4 for misassemblies (**Figure 1f** and **Figure 2**) by aligning the four *de-novo* assemblies (lycPyrIL, lycPyrPB, lycPyrSN1 and lycPyrSN2) to it using Satsuma2 (Grabherr et al., 2010) and chromosome models from three songbird outgroups (*Ficedula albicollis*, *Taeniopygia guttata* and *Parus major*) using LASTZ 1.04.00 (Harris, 2007). We identified misassemblies by looking for regions in which the different *de-novo* assemblies were in conflict with the final assembly (schematically showed in **Figure 2**). We applied the majority rule for each scaffolding or orientation conflict found between lycPyr4 and the four draft assemblies. To make any decisions against the scaffold configuration in lycPyr4, three of the four *de-novo* assemblies needed to be in discordance with lycPyr4 and show the same pattern of discordance. In cases where only two *de- novo* assemblies showed the same pattern of discordance and the other were not informative, we used the information provided by the outgroups to decide whether to keep the lycPyr4 scaffold configuration or correct it. With this approach we were able to identify 45 intra-scaffold misassemblies at a fine scale, all of them being orientation issues of PacBio contigs within scaffolds.

Next, we gap-filled the assembly using PBJelly (PBSuite 15.8.24; English et al. (2012)) with the default options except for the parameter -min 10 in order to consider the gaps longer than 10 bp

(**Figure 1g**). After the gap-filling step that used the PacBio reads, we ultimately polished the genome with long reads using Arrow (one run; PacBio library) and with short reads using Pilon (two runs; Illumina library; **Figure 1h**).

The last step of assembly curation involved the generation of Hi-C heatmaps on lycPyr5 by mapping the Hi-C library to the assembly using Juicer 1.5 (Durand et al. (2016); **Figure 1i**). We manually inspected the Hi-C maps for misassemblies using Juicebox 1.9.8 (https://github.com/aidenlab/Juicebox) and corrected lycPyr5 accordingly (**Supplementary Figure S3**). This way, we manually solved remaining assembly issues regarding the orientation and order of some contigs or scaffolds within the chromosome models, as well as corrected erroneous chromosomal translocations.

References

Bickhart, D. M., Rosen, B. D., Koren, S., Sayre, B. L., Hastie, A. R., Chan, S., . . . Smith, T. P. L. (2017). Single-molecule sequencing and chromatin conformation capture enable de novo reference assembly of the domestic goat genome. *Nature Genetics, 49*, 643. doi:10.1038/ng.3802

Burton, J. N., Adey, A., Patwardhan, R. P., Qiu, R., Kitzman, J. O., & Shendure, J. (2013). Chromosome-scale scaffolding of de novo genome assemblies based on chromatin interactions. *Nature Biotechnology, 31*, 1119. doi:10.1038/nbt.2727

Chin, C.-S., Peluso, P., Sedlazeck, F. J., Nattestad, M., Concepcion, G. T., Clum, A., . . . Schatz, M. C. (2016). Phased diploid genome assembly with single-molecule real-time sequencing. *Nature Methods, 13*, 1050. doi:10.1038/nmeth.4035

Durand, N. C., Shamim, M. S., Machol, I., Rao, S. S. P., Huntley, M. H., Lander, E. S., & Aiden, E. L. (2016). Juicer Provides a One-Click System for Analyzing Loop-Resolution Hi-C Experiments. *Cell Systems, 3*(1), 95-98. doi:https://doi.org/10.1016/j.cels.2016.07.002

Eid, J., Fehr, A., Gray, J., Luong, K., Lyle, J., Otto, G., . . . Turner, S. (2009). Real-Time DNA Sequencing from Single Polymerase Molecules. *Science, 323*(5910), 133-138. doi:10.1126/science.1162986

English, A. C., Richards, S., Han, Y., Wang, M., Vee, V., Qu, J., . . . Gibbs, R. A. (2012). Mind the Gap: Upgrading Genomes with Pacific Biosciences RS Long-Read Sequencing Technology. *PLOS ONE, 7*(11), e47768. doi:10.1371/journal.pone.0047768

Grabherr, M. G., Russell, P., Meyer, M., Mauceli, E., Alföldi, J., Di Palma, F., & Lindblad-Toh, K. (2010). Genome-wide synteny through highly sensitive sequence alignment: Satsuma. *Bioinformatics (Oxford, England), 26*(9), 1145-1151. doi:10.1093/bioinformatics/btq102 Harris, R. S. (2007). Improved pairwise alignment of genomic DNA. *Ph.D. Thesis, The Pennsylvania State University.*

Li, H., & Durbin, R. (2010). Fast and accurate long-read alignment with Burrows–Wheeler transform. *Bioinformatics, 26*(5), 589-595. doi:10.1093/bioinformatics/btp698

Li, H., Genome Project Data Processing, S., Wysoker, A., Handsaker, B., Marth, G., Abecasis, G., . . . Fennell, T. (2009). The Sequence Alignment/Map format and SAMtools. *Bioinformatics, 25*(16), 2078-2079. doi:10.1093/bioinformatics/btp352

Putnam, N. H., O'Connell, B. L., Stites, J. C., Rice, B. J., Blanchette, M., Calef, R., . . . Green, R. E. (2016). Chromosome-scale shotgun assembly using an in vitro method for long-range linkage. *Genome Research, 26*(3), 342-350. doi:10.1101/gr.193474.115

Walker, B. J., Abeel, T., Shea, T., Priest, M., Abouelliel, A., Sakthikumar, S., . . . Earl, A. M. (2014). Pilon: An Integrated Tool for Comprehensive Microbial Variant Detection and Genome Assembly Improvement. *PLOS ONE, 9*(11), e112963. doi:10.1371/journal.pone.0112963

Warren, R. L., Yang, C., Vandervalk, B. P., Behsaz, B., Lagman, A., Jones, S. J. M., & Birol, I. (2015). LINKS: Scalable, alignment-free scaffolding of draft genomes with long reads. *GigaScience, 4*(1), 35. doi:10.1186/s13742-015-0076-3

Yeo, S., Coombe, L., Warren, R. L., Chu, J., & Birol, I. (2017). ARCS: scaffolding genome drafts with linked reads. *Bioinformatics, 34*(5), 725-731. doi:10.1093/bioinformatics/btx675
